# Association between sedentary behavior, physical activity, and cardiovascular disease-related outcomes in adults—A meta-analysis and systematic review

**DOI:** 10.3389/fpubh.2022.1018460

**Published:** 2022-10-19

**Authors:** Zhi-de Liang, Meng Zhang, Chuan-zhi Wang, Yang Yuan, Jing-hong Liang

**Affiliations:** ^1^Cancer Institute of The Affiliated Hospital of Qingdao University and Qingdao Cancer Institute, Qingdao, China; ^2^Postgraduate Department, Xi'an Physical Education University, Xi'an, China; ^3^Department of Physical Education, College of Physical Education, Qingdao University, Qingdao, China; ^4^Department of Maternal and Child Health, School of Public Health, Sun Yat-sen University, Guangzhou, China

**Keywords:** physical activity, meta-analysis, healthy adult, cardiovascular disease(s), sedentary behavior

## Abstract

**Background:**

Sedentary behavior (SB) and physical activity (PA) are modifiable risk factors for cardiovascular disease (CVD); however, previous research on the effects of PA and SB on CVD has been relatively homogeneous. Our study investigated the association between PA, SB, and CVD-related outcomes.

**Methods:**

A comprehensive search strategy was conducted in the MEDLINE, Embase, Cochrane Library, and Web of Science databases from their inception to September 2022. We identified eligible studies according to PICOS: the populations comprised healthy adults, the interventions or exposures were PA or SB, the outcomes were CVD-related outcomes, and the study designs were randomized controlled trials (RCTs) and longitudinal studies (LS). Outcomes were pooled using fixed or random effects models, and the quality of individual studies was assessed by the Cochrane Risk of Bias Instrument and the Newcastle Ottawa Scale.

**Results:**

A total of 148 RCTs and 36 LS were included, comprising a total of 75,075 participants. The study quality was rated as low to moderate. We found an increased hazard ratio (HR) for CVD in the population with SB (HR = 1.34; 95% confidence interval [CI]: 1.26 to 1.43; I^2^ = 52.3%; P_heterogeneity_ < 0.001, random model) and a decreased HR for CVD in those who performed long-term PA (HR = 0.71; 95% CI: 0.66 to 0.77; I^2^ = 78.0%, P_heterogeneity_ < 0.001, random model). Long-term PA improved the lipid profiles in healthy adults; participants in this group exhibited increased high-density lipoprotein (weighted mean difference [WMD] = 2.38; 95% CI: 1.00 to 3.76; I^2^ = 84.7%; P_heterogeneity_ < 0.001, random model), decreased triglycerides (WMD = −7.27; 95% CI: −9.68 to −4.87; I^2^ = 0%, P_heterogeneity_ = 0.670, fixed model), and lower total-cholesterol (WMD = −6.84; 95% CI: −9.15 to −4.52; I^2^ = 38.4%, P_heterogeneity_ < 0.001, random model).

**Conclusions:**

Long-term SB increases the risk of CVD in healthy adults, whereas PA reduces the risk of CVD and improves indicators associated with CVD. However, the ability of PA to improve blood lipids appeared to be limited. The detailed association of SB and PA on CVD needs to be further investigated in the future.

## Introduction

Cardiovascular disease (CVD) is a general term for a group of cardiovascular disorders, including coronary heart disease (CHD), heart failure (HF), aortic atherosclerosis, cerebrovascular disease, and peripheral artery disease (1). The advancement of medical technology and the dramatic improvement of the health environment have greatly reduced the mortality rate from infectious diseases and substantially increased life expectancy. In developed countries as well as some developing ones, chronic diseases, especially CVD, have become the leading cause of death (2). For example, in China, CVD accounts for 40% of deaths in the Chinese population (3), and the incidence of CVD increased by 14.7% between 1990 and 2016 (4).

The causes of CVD are multifactorial, and many CVD risk factors are not modifiable; these include age, gender, race, and family history of heart disease. However, other risk factors are modifiable; these include smoking, alcohol abuse, obesity, physical inactivity, a sedentary lifestyle, diet, hypertension, diabetes, and dyslipidemia (5, 6). Sedentary behavior (SB) is defined as spending waking hours in a sitting, reclined, or lying position and is characterized by low energy expenditure (≤1.5 metabolic equivalents for task [METs]) (5). Several studies have demonstrated that SB is a major modifiable risk factor for CVD (7–9), and sitting for more than 6 h per day is associated with an increased risk of 12 common chronic diseases. However, replacing sitting time with physical activity (PA) of varying intensities may reduce the risk of these chronic diseases (10). Therefore, PA has been proposed as an alternative strategy to improve quality of life and reduce the risk of CVD (11). The World Health Organization defines PA as “any physical movement produced by skeletal muscle that results in energy expenditure,” and PA can be categorized into different types depending on the exercise mode, intensity, frequency, and duration (12). A meta-analysis comparing the effects of five types of PA on cardiometabolic health in people who were obese or overweight found that all types of PA played a key role in improving cardiometabolic health (13). Another meta-analysis demonstrated that PA reduced fatigue in patients with cancer and those who had received hematopoietic stem cell transplants (14). Other studies have revealed that PA reduces mortality, improves the quality of life of patients with CVD, and protects against damage at the early stages of myocardial infarction (15, 16).

Although previous studies have shown that PA can significantly improve the quality of life of patients with CVD (17, 18), research in this area is still insufficient. First, the results of previous studies on the association between CVD and PA are limited because they have focused primarily on cardiovascular mortality (CVM); few studies have investigated other CVD-related outcomes (19, 20). Therefore, the present study examined the effects of PA on all CVD outcomes to help fill the knowledge gap resulting from the one-sidedness of previous studies, and it aimed to elucidate the different effects of PA on CVM and other CVD outcomes. Second, several studies have found that PA reduces the risk of cardiovascular disease, but they did not distinguish between the age, region, and economic status of the participants (21). Third, the size and diversity of populations investigated in previous studies have been limited, including those with underlying diseases, and further research is needed to ensure that these findings are generalizable to healthy people (22). Fourth, CVD is a chronic disease that requires evidence not only from randomized controlled trials, but also from longitudinal studies, but previous studies have focused on only one of these (23, 24). Therefore, combining these two types of evidence can better analyze the association between PA, SB and CVD. Moreover, the number of published studies on the effects of SB and PA on CVD has increased significantly in recent years (25, 26). However, because of the increased volume of data, updated reviews are needed to provide more reliable evidence. Therefore, we conducted a comprehensive systematic review and meta-analysis of the literature to characterize and quantify the association between PA, SB, and CVD-related outcomes.

## Methods

This meta-analysis was conducted following the recommendations of the Cochrane Collaboration Handbook (27) and the framework for meta-analysis of longitudinal studies in epidemiology (28). No ethical approval or patient consent was required because all analyses were performed using data from previous studies.

### Search strategies and study selection

To identify publications on PA and SB in healthy adults, an exhaustive, strategic literature search of the MEDLINE, Embase, Cochrane Library, and Web of Science databases was conducted from their inception until September 2, 2022. The search strategy did not include any restrictions and consisted mainly of medical subject headings associated with keywords, free words, and Boolean operators. The detailed search strategy is described in the Appendix (p. 1); in brief, the following search terms were used: “cardiovascular disease,” “sedentary behavior,” “adults,” “exercise,” “training,” “physical activity,” “aerobic exercise,” “triglyceride,” “glucose,” “lipoproteins,” and “randomized controlled trial.”

In addition, a recursive search for relevant publications was conducted by manually searching the bibliographic lists of similar reviews and large professional conferences. At least two investigators (ZM and LZ) performed the study selection and data extraction independently, and discrepancies that arose during the process were resolved by consulting a third investigator (YY). Title and abstract screening was used to eliminate duplicates, reviews, and irrelevant studies. Subsequently, potentially eligible studies were screened, and their full text was downloaded; those that could not be downloaded in full were excluded. The selected citations were independently cross-checked for completeness and accuracy by two investigators. All citations were managed and analyzed with EndNote X9 software (Thompson ISI Research Soft, Clarivate Analytics, Philadelphia, Philadelphia, USA).

### Inclusion and exclusion criteria and data abstraction

Studies that met the following eligibility criteria were included.

### Population

Participants were healthy adults aged 18 years or older; were physically independent; had no current cardiovascular or other significant medical conditions; had no history of medical conditions preventing them from participating in the exercise intervention; were not currently taking any medications; and had not engaged in regular PA in the past 1–2 years according to a physician report or self-report (obtained through a standard diagnostic interview).

### Interventions and exposure

Studies on PA interventions in healthy adults were included. All participants received the PA intervention at least twice a week for at least 8 weeks, as recommended by the American College of Sports Medicine Guidelines (29). PA was defined as any type of body activity that resulted in energy expenditure (12). The specific breakdown of PA types was as follows (30, 31): (1) Aerobic exercise (AE), defined as exercise to improve cardiovascular health, including walking, running, and cycling. (2) Resistance exercise (RE), defined as exercise to increase muscle strength, such as using elastic bands and dumbbells. (3) Multicomponent exercise (ME), defined as a combination of at least two exercise types, such as AE, RE, and balance training; and (4) Mind–body exercise (MBE), defined as exercise to improve participants' physical and mental coordination through awareness exercises, such as Tai chi, yoga, and dance. In the interventions described above, PA was performed with and without supervision.

In addition, longitudinal studies on SB and PA in healthy adults were included to investigate the association between PA, SB, and CVD events (fatal or non-fatal). SB was defined as waking behavior characterized by low energy expenditure (≤1.5 METs) (5), including sitting or reclining at leisure, at work, in traffic, and at home. SB and PA time was obtained by a standardized questionnaire or self-report. If more than two PA or SB levels were present in a study, they were categorized into two groups—high PA or SB and low PA or SB—and the low PA or SB group was considered the reference category.

### Comparators

The comparators included a non-exercise control group, a health education group, and a group that maintained their current lifestyle.

### Outcomes

The primary outcomes of randomized controlled trials (RCTs) were lipid levels (triglycerides [TGs], total cholesterol [TC], high-density lipoprotein [HDL], and low-density lipoprotein [LDL]), and various clinical measures were used to assess changes in these metrics from baseline to endpoint. The secondary outcomes were other indicators related to CVD risk factors, namely body mass index (BMI), systolic blood pressure (SBP), diastolic blood pressure (DBP), and blood glucose.

For longitudinal studies, any type of CVD incidence or mortality was selected as the primary outcome; this indicator was included in most studies and was considered an appropriate choice for acceptability.

### Study design

Our study included all types of randomized controlled trials (RCTs), and to further explore the effect of SB and PA on CVD incidence, population-based longitudinal studies (including any type of prospective cohort or retrospective cohort study) were also included; no restrictions were placed on publication date, race, or region. Studies were excluded if (1) the study type was a systematic review, meta-analysis, or protocol; (2) the full text of the data analysis or data details were not available; or (3) the intervention method was a combination of PA and other methods.

### Outcome measurement and quality assessment

The following key information was extracted: first author; year of publication; source; intervention or follow-up time; intervention dose; study type; sample size; and baseline characteristics of the participants, such as age and gender. If the baseline data were not available in the text, they were obtained by contacting the corresponding author.

The quality of the RCT was independently assessed by two reviewers (ZM and ZD) according to the Cochrane Risk of Bias tool (32), which consists of seven items: random sequence generation; allocation concealment; participant and personnel blinding; outcome assessment blinding; incomplete outcome data; selective reporting and other biases; and items judged as having high risk, low risk, and unclear risk.

All prospective and retrospective studies were evaluated by the Newcastle–Ottawa Scale (NOS), which consists of three main items: patient selection, comparability of the intervention and observation groups, and assessment of outcomes. Studies with NOS scores ≥7 were considered high-quality (33, 34). Two reviewers scored each study independently, and in cases of disagreement, a third reviewer reviewed their assessments.

### Statistical analyses

A traditional paired meta-analysis was conducted for the trials that satisfied the inclusion criteria (27). For the results of continuous variables, baseline and terminal mean differences and standard deviation (SD) were extracted; if they were not provided, different methods were used to convert the data to a standard format (35, 36). If the study was a multi-arm RCT, all PA and control group data were extracted. A quantitative pooled analysis was performed according to a fixed-effects model (inverse variance), and forest plots were constructed to derive the weighted mean difference (WMD) and 95% confidence interval (CI); if heterogeneity existed, a random effects model was used (D-L heterogeneity approach) (27). Hazard ratios (HRs) and their 95% CIs were included as pooled effects for the longitudinal studies. Because such variables must be symmetric and follow a normal distribution, logarithmic transformation was performed, and a random effects model was then used to explain the effects of between-study heterogeneity (37).

The presence of heterogeneity was determined by examining the forest plots and I^2^ statistics according to the latest version of the Cochrane Handbook (27). I^2^ statistics were used to assess statistical heterogeneity, with estimates of 25, 50, and 75% indicating mild, moderate, and high heterogeneity, respectively; a *P*-value of < 0.1 was considered statistically significant (38, 39). Sensitivity analyses and a series of subgroup analyses were performed to identify the sources of heterogeneity by statistical methods, and the literature-by-deletion method was employed to analyze sensitivity. Funnel plots were used to check for publication bias, which mainly arises from publication bias, selective reporting, or other sources. Finally, Egger's tests were carried out to quantitatively evaluate whether the studies had publication bias, and *P* < 0.05 indicated the absence of publication bias (40). To explore the sources of heterogeneity and the relationships of primary outcomes in different conditions, a subgroup analysis was performed; variables of interest included the region (the U.S. vs. other countries; developed vs. developing countries), the intervention time or follow-up time (≤12 weeks vs. >12 weeks or ≤10 years vs. >10 years, respectively), age ( ≤60 years vs. >60 years), quality of the literature (NOS <7 vs. NOS ≥7), gender distribution (male ≥ female vs. male < female), sample size (≤10000 vs. >10000), CVD outcomes (fatal vs. non-fatal), year of publication (≤2015 vs. >2015; ≤2016 vs. >2016), SB vs. no SB, and PA type (AE, RE, ME, and MBE). The above analyses were performed in STATA software version 15.1 (Stata, Corp, College Station, Texas, USA).

## Results

### Study selection and characteristics of the included studies

**Figure 1:**
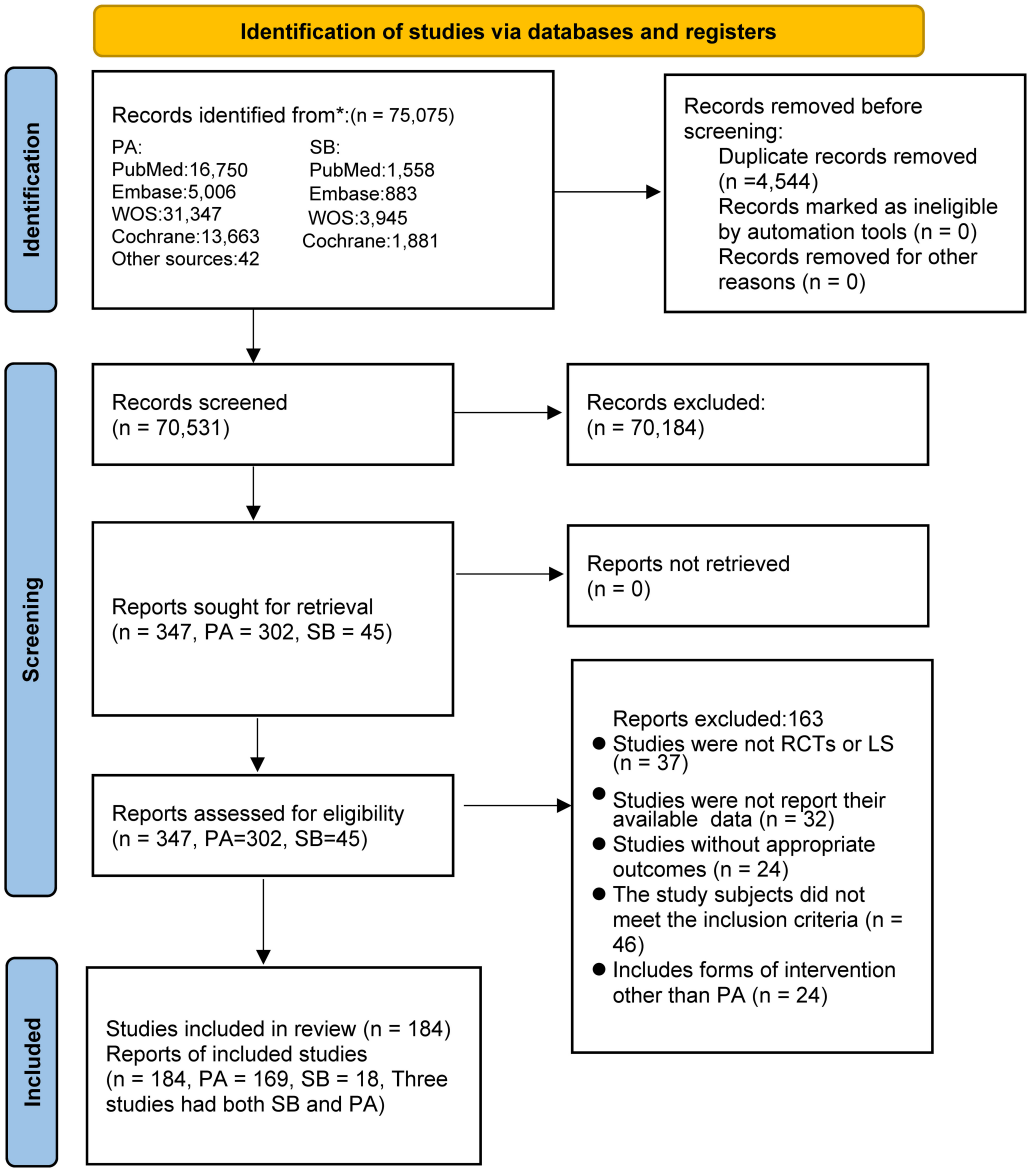
Literature review flowchart. LS, Longitudinal studies; PA, physical activity; RCT, randomized controlled trial; SB, sedentary behavior; WOS, web of science.

We obtained 75,075 studies from the database and identified 42 studies through other sources; of these, 4,544 studies were excluded because of duplication, and 70,184 were excluded on the basis of their titles and abstracts. After full-text screening of the remaining 347 studies, a total of 163 publications were excluded for the following reasons: 37 studies did not meet the study type criteria; 56 studies did not have appropriate outcomes or did not provide data that could be analyzed; 46 studies included participants that did not satisfy the inclusion and exclusion criteria (patients with underlying diseases, younger than 18 years, or currently taking medication); and 24 studies included interventions other than PA. Ultimately, we included 184 studies, of which 148 were RCTs and 36 were longitudinal studies (Supplementary Table S1). Three longitudinal studies assessed both PA and SB. The study selection process is shown in Figure 1.

A total of 1,011,473 participants aged between 18 and 100 years old were recruited in the 184 included studies; 8,166 and 1,003,307 participants were included in the RCTs and the longitudinal studies, respectively. The proportion of male participants was lower than that of female participants (males in RCTs: 2,686 [32.9%]; males in longitudinal studies: 412,519 [41.1%]). The intervention duration in the RCTs ranged between 8 weeks and 24 months, and the mean follow-up time in the longitudinal studies was 11.4 years. Participants were mainly recruited in Europe (*N* = 61, 33.2%) and North America (*N* = 55, 31.5%). We extracted several indicators related to CVD risk from the RCTs, including BMI (*N* = 89), blood pressure (SBP: *N* = 65, DBP: *N* = 63), blood lipids (TGs: *N* = 76, TC: *N* = 79, HDL: *N* = 84, LDL: *N* = 78), and blood glucose (*N* = 65). The demographic characteristics of the included studies are summarized in Supplementary Table S1. Subgroup analyses of the main outcomes are shown in Table 1.

**Table 1:** Outcomes and subgroup analysis based on indicators related to CVD.

**Type of research and** **meta–analyses variables**		**Number of studies** **(included multi–arm** **study)**	**Number of participants**		**Weighted mean difference**	**Heterogeneity**	
			**Intervention**	**CG**		**I^2^(%)**	**P**
**RCT**							
**Primary**							
Lipids	TG	76	2,386	2,272	−7.27 (−9.68 to −4.87)	0	0.670
	TC	79	2,298	2,214	−6.84 (−9.15 to −4.52)	38.4	<0.001
	HDL	84	2,568	2,450	2.38 (1.00 to 3.76)	84.7	<0.001
	LDL	78	2,435	2,322	−5.80 (−8.04 to −3.57)	65.5	<0.001
**Secondary outcomes**							
Others	BMI	89	3,204	3,016	−0.36 (−0.43 to −0.30)	0	0.998
	SBP	65	1,820	1,680	−3.63 (−4.62 to −2.64)	58.0	<0.001
	DBP	63	1,759	1,614	−2.25 (−2.94 to −1.56)	51.3	<0.001
	GlU	65	1,743	1,670	−4.40 (−5.44 to −3.36)	86.5	<0.001
**Subgroup analysis based on TG**							
Region	Overall	76	2,386	2,272	−7.27 (−9.68 to −4.87)	0	0.670
	Developing countries	13	346	345	−13.83 (−19.88 to −7.79)	0	0.665
	Developed countries	63	2,040	1,927	−6.04 (−8.66 to −3.41)	0	0.727
Intervention time	Overall	76	2,386	2,272	−7.27 (−9.68 to −4.87)	0	0.670
	≤12weeks	41	1,026	944	−7.74 (−11.11 to −4.37)	12.1	0.228
	>12weeks	35	1,360	1,328	−6.79 (−10.23 to −3.35)	0	0.929
Published year	Overall	76	2,386	2,272	−7.27 (−9.68 to −4.87)	0	0.670
	≤2015	54	1,651	1,582	−9.30 (−12.46 to −6.15)	0	0.522
	>2015	22	735	690	−4.34 (−8.16 to −0.71)	0	0.884
Age	Overall	75	2,366	2,224	−7.31 (−9.72 to −4.90)	0	0.653
	≤60	54	1,782	1,596	−6.27 (−9.24 to −3.30)	0	0.928
	>60	21	584	628	−9.33 (−13.45 to −5.20)	30.4	0.076
Male to female ratio	Overall	72	2,264	1,982	−7.26 (−9.744 to −4.77)	0	0.555
	Female<male	51	1,685	1,483	−6.34 (−9.40 to −3.28)	0	0.495
	Male≥female	21	579	499	−9.03 (−13.29 to −4.78)	0	0.574
Sedentary	Overall	76	2,386	2,272	−7.27 (−9.68 to −4.87)	0	0.670
	Yes	45	1,472	1,438	−7.18 (−10.31 to −4.04)	0	0.918
	No/NR	31	914	834	−7.41 (−11.16 to −3.65)	17.8	0.163
Exercise type	Overall	76	2,386	2,272	−7.27 (−9.68 to −4.87)	0	0.670
	AE	55	1,550	1,455	−6.99 (−10.04 to −3.94)	0	0.525
	RE	16	314	303	−10.02 (−16.36 to −3.68)	0	0.823
	ME	16	499	491	−6.80 (−12.15 to −1.45)	10.6	0.332
	MBE	2	23	23	−3.40 (−16.84 to −10.04)	10.0	0.292
**Subgroup analysis based on TC**							
Region	Overall	79	2,298	2,214	−6.84 (−9.15 to −4.52)	38.4	<0.001
	Developing countries	15	385	382	−11.03 (−18.47 to −3.60)	64.0	<0.001
	Developed countries	64	1,913	1,832	−5.93 (−8.08 to −3.78)	19.3	<0.1
Intervention time	Overall	79	2,298	2,214	−6.84 (−9.15 to −4.52)	38.4	<0.001
	≤12weeks	43	1,054	971	−7.72 (−11.56 to −3.89)	45.7	<0.001
	>12weeks	36	1,244	1,243	−6.06 (−8.79 to −3.33)	27.0	<0.1
Published year	Overall	79	2,298	2,214	−6.84 (−9.15 to −4.52)	38.4	<0.001
	≤2015	56	1,720	1,681	−6.82 (−9.54 to −4.09)	38.0	<0.01
	>2015	33	578	533	−6.84 (−11.24 to −2.45)	39.2	<0.1
Age	Overall	77	2,269	2,188	−6.54 (−8.83 to −4.26)	36.8	<0.001
	≤60	54	1,641	1,509	−6.81 (−9.40 to −4.22)	31.9	<0.01
	>60	23	628	679	−6.56 (−11.41 to −1.70)	48.2	<0.01
Male to female ratio	Overall	75	2,067	1,915	−6.99 (−9.37 to −4.60)	37.6	<0.001
	Female<male	51	1,557	1,465	−6.52 (−9.49 to −3.55)	43.5	<0.001
	Male≥female	24	510	450	−8.47 (−12.38 to −4.56)	20.4	0.164
Sedentary	Overall	79	2,298	2,214	−6.84 (−9.15 to −4.52)	38.4	<0.001
	Yes	48	1,462	1,423	−5.05 (−7.92 to −2.19)	31.1	0.011
	No/NR	31	836	791	−9.31 (−12.96 to −5.67)	41.5	0.004
Exercise type	Overall	79	2,298	2,214	−6.84 (−9.15 to −4.52)	38.4	<0.001
	AE	58	1503	1417	−5.70 (−8.53 to −2.88)	35.4	<0.01
	RE	16	306	296	−8.77 (−16.51 to −1.04)	53.1	<0.01
	ME	17	466	478	−8.58 (−13.32 to −3.84)	34.5	<0.1
	MBE	2	23	23	−14.39 (−34.10 to 5.32)	62.4	0.103
**Subgroup analysis based on HDL**							
Region	Overall	84	2,568	2,450	2.38 (1.00 to 3.76)	84.7	<0.001
	Developing countries	18	406	406	2.34 (0.77 to 3.90)	27.3	0.117
	Developed countries	76	2,162	2,044	2.35 (0.68 to 4.01)	87.2	<0.001
Intervention time	Overall	84	2,568	2,450	2.38 (1.00 to 3.76)	84.7	<0.001
	≤12weeks	45	1,150	1,058	2.28 (−0.16 to 4.72)	89.3	<0.001
	>12weeks	39	1,418	1,392	2.43 (1.04 to 3.83)	70.4	<0.001
Published year	Overall	84	2,568	2,450	2.38 (1.00 to 3.76)	84.7	<0.001
	≤2015	60	1,790	1,715	2.83 (0.95 to 4.71)	88.0	<0.001
	>2015	24	778	735	0.95 (−0.18 to 2.08)	26.0	0.101
Age	Overall	83	2,548	2,433	2.34 (0.96 to 3.72)	84.6	<0.001
	≤60	59	1,898	1,731	2.07 (1.05 to 3.10)	56.6	<0.001
	>60	24	650	702	2.90 (−1.50 to 7.31)	94.7	<0.001
Male to female ratio	Overall	82	2,346	2,160	2.63(1.17 to 4.08)	85.2	<0.001
	Female<male	58	1,722	1,620	2.43 (0.37 to 4.49)	89.0	<0.001
	Male≥female	24	624	540	2.76 (1.53 to 3.98)	32.9	<0.100
Sedentary	Overall	84	2,568	2,450	2.38 (1.00 to 3.76)	84.7	<0.001
	Yes	47	1,515	1,474	1.17 (−0.75 to 3.09)	85.5	<0.001
	No/NR	37	1,053	976	3.84 (1.90 to 5.80)	82.1	<0.001
Exercise type	Overall	84	2,568	2,450	2.38 (1.00 to 3.76)	84.7	<0.001
	AE	61	1715	1590	2.71 (0.79 to 4.64)	88.6	<0.001
	RE	19	362	353	3.37 (1.09 to 5.66)	43.4	<0.1
	ME	18	468	484	0.49 (−2.06 to 3.04)	75.8	<0.001
	MBE	2	23	23	0.45 (−8.15 to 9.04)	55.9	0.132
**Subgroup analysis based on LDL**							
Region	Overall	78	2,435	2,322	−5.80 (−8.04 to −3.57)	65.5	<0.001
	Developing countries	14	354	357	−7.68 (−12.85 to −2.52)	58.8	<0.1
	Developed countries	64	2,081	1,965	−5.38 (−7.87 to −2.88)	66.9	<0.001
Intervention time	Overall	78	2,435	2,322	−5.80 (−8.04 to −3.57)	65.5	<0.001
	≤12weeks	39	1,003	921	−7.14 (−11.20 to −3.08)	77.1	<0.001
	>12weeks	39	1,432	1,401	−3.58 (−5.68 to −1.47)	31.6	<0.1
Published year	Overall	78	2,435	2,322	−5.80 (−8.04 to −3.57)	65.5	<0.001
	≤2015	57	1,868	1,798	−4.75 (−7.33 to −2.16)	66.9	<0.001
	>2015	21	567	524	−8.94 (−13.47 to −4.41)	61.0	<0.001
Age	Overall	77	2,405	2,305	−5.72 (−7.95 to −3.49)	65.5	<0.001
	≤60	55	1,801	1,641	−3.71 (−5.44 to −1.98)	17.7	0.103
	>60	22	614	664	−8.25 (−14.06 to −2.44)	86.9	<0.001
Male to female ratio	Overall	74	2,213	2,032	−6.32 (−8.65 to −3.98)	67.1	<0.001
	Female<male	51	1,626	1,527	−6.44 (−9.37 to −3.51)	72.5	<0.001
	Male≥female	23	587	505	−5.73 (−9.43 to −2.02)	44.4	<0.01
Sedentary	Overall	78	2,435	2,322	−5.80 (−8.04 to −3.57)	65.5	<0.001
	Yes	47	1,518	1,475	−2.80 (−5.09 to −0.52)	35.1	<0.01
	No/NR	31	917	847	−9.06 (−12.81 to −5.31)	74.6	<0.001
Exercise type	Overall	78	2,435	2,322	−5.80 (−8.04 to −3.57)	65.5	<0.001
	AE	55	1,503	1,412	−3.93 (−6.31 to −1.54)	50.6	<0.001
	RE	17	330	320	−10.87 (−19.49 to −2.25)	83.2	<0.001
	ME	20	579	567	−4.47 (−7.04 to −1.90)	3.5	0.413
	MBE	2	23	23	−9.76 (−21.79 to 2.26)	0	0.336
**Type of research and** **meta–analyses variables**		**Number of studies** **(included multi–arm** **study)**	**Number of** **participants**		**Hazard ratio**	**Heterogeneity**	
			**Exposure**	**REF**		**I** ^2^ **(%)**	**P**
**LS**							
SB	HR	18	166,816	216,796	1.34 (1.26 to 1.43)	52.3	<0.01
PA	HR	21	239,479	380,216	0.71 (0.66 to 0.77)	78.0	<0.001
Subgroup analysis based on SB							
Region	Overall	18	166,816	216,796	1.34 (1.26 to 1.43)	52.3	<0.01
	United States	11	152,605	184,233	1.33 (1.25 to 1.42)	52.4	<0.01
	Others	7	14,211	32,563	1.42 (1.11 to 1.81)	60.0	<0.1
Published year	Overall	18	166,816	216,796	1.34 (1.26 to 1.43)	52.3	<0.01
	≤2016	13	135,478	187,115	1.32 (1.23 to 1.42)	59.8	<0.01
	>2016	5	31,338	29,681	1.44 (1.23 to1.68)	23.0	0.261
Follow–up time	Overall	18	166,816	216,796	1.34 (1.26 to 1.43)	52.3	<0.01
	≤10 years	12	125,900	173,235	1.32 (1.23 to 1.42)	47.4	<0.1
	>10 years	6	40,916	43,561	1.39 (1.23 to 1.59)	64.1	<0.1
Number of follow–up participants	Overall	17	166,816	216,796	1.34 (1.26 to 1.43)	55.1	<0.01
	≤10,000	9	9,817	11,497	1.56 (1.37 to 1.77)	21.2	0.255
	>10,000	8	156,999	205,299	1.28 (1.20 to 1.36)	49.1	<0.1
Disease type	Overall	18	166,816	216,796	1.34 (1.26 to 1.43)	52.3	<0.01
	CVM	7	90,730	94,255	1.32 (1.22 to 1.44)	36.5	0.126
	CVD	12	76,086	122,541	1.36 (1.24 to 1.50)	70.9	<0.01
Male to female ratio	Overall	16	165,944	215,954	1.34 (1.26 to 1.43)	55.1	<0.01
	Female<male	11	99,358	116,547	1.33 (1.22 to 1.44)	61.7	0.001
	Male≥female	5	66,586	99,407	1.37 (1.28 to 1.46)	0	0.575
Quality of literature	Overall	17	166,816	216,796	1.34 (1.26 to 1.43)	52.3	<0.01
	<7	9	128,139	175,653	1.39 (1.29 to 1.49)	8.4	0.365
	≥7	8	38,677	44,143	1.30 (1.20 to 1.42)	58.9	<0.01
Subgroup analysis based on PA							
Region	Overall	21	239,479	380,216	0.71 (0.66 to 0.77)	78.0	<0.001
	United States	8	180,416	262,215	0.67 (0.58 to 0.78)	82.0	<0.001
	Others	13	59,063	118,001	0.75 (0.70 to 0.81)	55.0	<0.01
Published year	Overall	21	239,479	380,216	0.71 (0.66 to 0.77)	78.0	<0.001
	≤2016	10	56,980	72,557	0.70 (0.65 to 0.76)	0	0.683
	>2016	11	182,499	307,659	0.73 (0.65 to 0.82)	88.0	<0.001
Follow–up time	Overall	21	239,479	380,216	0.71 (0.66 to 0.77)	78.0	<0.001
	≤10 years	11	65,724	159,415	0.74 (0.68 to 0.80)	56.2	<0.1
	>10 years	10	173,755	220,801	0.69 (0.61 to 0.79)	83.2	<0.001
Number of follow–up participants	Overall	19	239,479	380,216	0.71 (0.66 to 0.77)	78.0	<0.001
	≤10,000	8	14,011	178,75	0.66 (0.56 to 0.79)	75.9	<0.001
	>10,000	11	225,468	362,341	0.78 (0.73 to 0.83)	51.9	<0.1
Disease type	Overall	21	239,479	380,216	0.71 (0.66 to 0.77)	78.0	<0.001
	CVM	11	188,625	266,336	0.72 (0.65 to 0.80)	54.4	<0.1
	CVD	10	50,854	76,256	0.71 (0.63 to 0.80)	85.8	<0.001
Male to female ratio	Overall	21	239,479	380,216	0.71 (0.66 to 0.77)	78.0	<0.001
	Female<male	13	210,071	231,408	0.71 (0.64 to 0.78)	81.7	<0.001
	Male≥female	4	29,408	38,799	0.81 (0.69 to 0.96)	61.7	<0.1
Quality of literature	Overall	21	239,479	380,216	0.71 (0.66 to 0.77)	78.0	<0.001
	<7	7	29,513	83,560	0.77 (0.70 to 0.86)	22.5	0.243
	≥7	14	209,966	296,656	0.70 (0.63 to 0.77)	84.1	<0.001

If studies reported with more than two categories of different SB, PA levels, they were converted into two groups, namely high-level SB, PA and low-level SB, PA while the later group was used as the reference category. Pool effect size: pooled WMDs (95% Confidence interval [CI]); pooled HRs(95% CI). BMI, Body mass index; CVD, Cardiovascular disease; CVM, Cardiovascular mortality; CG, Control Group; DBP, Diastolic blood pressure; HDL, High density lipoprotein; HR, Hazard Ratio; GlU, Glucose; LS, Longitudinal study; PA, Physical activity; REF, Reference; RCT, Randomized controlled trial; SBP, Systolic blood pressure; TG, Triglyceride; TC, Total cholesterol; LDL, Low density lipoprotein.

And the forest plots and funnel plots of all outcomes will be presented in the Appendix (p. 23–37).

### Quality of the included studies

Of the 148 RCTs, 36 had a low risk of bias in random sequence generation, 17 had a low risk of selection bias, 18 had a low risk of performance bias, and 27 had a low risk of detection bias. Only six studies had a high risk of abrasion bias, and most studies had a low risk of reporting bias (143/148, 97.3%). The detailed assessment process is shown in Supplementary Figures S1, S2.

Of the 36 longitudinal studies, 29 received a score of 7 and were considered high-quality according to the NOS criteria; the detailed assessment process is shown in Supplementary Figure S3.

### Primary outcomes

#### RCTs

Of the RCTs, 76 studies (4,658 participants) explored the effects of long-term PA on TGs, 79 studies (4,512 participants) described the effects of PA on TC, 84 studies (5,018 participants) investigated the effect of PA on HDL, and 78 studies (4,757 participants) described the effects of PA on LDL (Table 1). Most studies reported fasting lipids; only two studies (41, 42) reported non-fasting lipids. We pooled the WMD effect sizes according to a fixed model or a random model. The results showed that long-term PA significantly increased HDL levels (*P* < 0.001) and decreased TGs (*P* < 0.001), TC (*P* < 0.001), and LDL (*P* < 0.001). The pooled results for TG levels were as follows: WMD = −7.27; 95% CI: −9.68 to −4.87; I^2^ = 0%, P_heterogeneity_ = 0.670 (fixed model); TC: (WMD = −6.84; 95% CI: −9.15 to −4.52; I^2^ = 38.4%, P_heterogeneity_ < 0.001, random model); HDL: (WMD = 2.38; 95% CI: 1.00 to 3.76; I^2^ = 84.7%; P_heterogeneity_ < 0.001, random model); LDL: (WMD = −5.80; 95% CI: −8.04 to −3.57; I^2^ = 65.5%, P_heterogeneity_ < 0.001, random model). Among the four lipid indices, only the funnel plot for TC showed symmetry; the rest of the indices did not show symmetry (Supplementary Figures S17–S20). The Egger's test results were as follows: TGs, *P* < 0.05; TC, *P* = 0.937; HDL, *P* = 0.240; and LDL, *P* < 0.05.

To explore possible sources of heterogeneity, subgroup analyses of lipid outcomes were performed, grouping participants according to intervention time, region, age of participants, year of publication, male/female ratio, previous SB or no previous SB, and PA type. Some of the subgroup analyses were consistent; however, when studies were divided according to their male/female ratios, less heterogeneity was observed in the combined effect of long-term PA on TC in the studies with more males (WMD = −8.47; 95% CI: −2.38 to −4.56; I^2^ = 20.4%, *P* = 0.164, random model). Similarly, when studies were divided according to the type of PA, less heterogeneity was observed in studies on the effect of ME on LDL (WMD = −4.47; 95% CI: −7.04 to −1.90; I^2^ = 20.4%, *P* = 0.164, random model). In addition, PA was associated with a significant improvement in HDL only if participants were younger than 60 years old (*P* < 0.001), the intervention duration was longer than 12 weeks (*P* < 0.01), the study was conducted in people without previous SB habits, or the study assessed AE (*P* < 0.01) or RE (*P* < 0.01). The detailed subgroup analyses are presented in Table 1.

### Longitudinal studies

Eighteen studies analyzed the effect of SB on CVD (Table 1). The results showed that people with SB had a significantly increased risk of CVD (*P* < 0.001), both fatal and non-fatal, with a pooled effect size (HR) of 1.34 (95% CI: 1.26 to 1.43) and relatively high heterogeneity (I^2^ = 52.3%, P_heterogeneity_ < 0.001, random model). The funnel plot was asymmetric, and the publication bias was high (P^Egger′s test^ < 0.05) (Supplementary Figure S22).

Twenty-one studies explored the effect of PA on CVD (Table 1). The results showed that long-term PA was associated with a significantly lower risk of CVD compared with low levels of PA (*P* < 0.001), with a pooled effect size (HR) of 0.71 (95% CI: 0.66 to 0.77) and high heterogeneity (I^2^ = 78.0%, P_heterogeneity_ < 0.001, random model). The funnel plot was also asymmetric, showing potential publication bias (P^Egger′s test^ = 0.605) (Supplementary Figure S23).

We performed subgroup analyses by dividing the studies according to several variables of interest (region, year of publication, male/female ratio, number of participants, follow-up time, quality of literature, and type of CVD), and some subsets of these subgroup analyses revealed sources of heterogeneity. For example, in the SB and CVD studies, heterogeneity was significantly lower in studies with population sizes under 10,000 (HR = 1.56; 95% CI: 1.37 to 1.77; I^2^ = 21.2%, *P* = 0.255, random model) than in studies with population sizes greater than 10,000 (HR = 0.28; 95% CI:1.20 to 1.36; I^2^ = 49.1%, *P* = 0.033, random model). Other sources of heterogeneity are detailed in Table 1.

### Secondary outcomes

#### RCTs

##### BMI

According to the pooled combined effects of BMI using a fixed effects model, 89 RCTs (6,220 participants) revealed a significant effect regarding BMI reduction (WMD = −0.36; 95% CI: −0.43 to −0.30; I^2^ = 0%, P_heterogeneity_ = 0.998, fixed model) (Table 1); however, the asymmetry of the funnel plot of BMI indicators suggested potential publication bias (P^Egger′s test^ = 0.051) (Supplementary Figure S14).

### Blood pressure

A total of 65 RCTs (3,500 participants) explored the effects of PA on SBP, and 63 (3,373 participants) investigated the effects of PA on DBP (Table 1). The resting blood pressure in both sitting and supine positions was included. The combined effect showed a significant reduction in SBP (WMD = −3.63; 95% CI: −4.62 to −2.64; I^2^ = 58.0%, P_heterogeneity_ < 0.001, random model) with some heterogeneity. DBP also decreased significantly by a small margin (WMD = −2.25; 95% CI: −2.94 to −1.56; I^2^ = 51.3%, P_heterogeneity_ < 0.001, random model), and heterogeneity was observed. The forest plots for both indicators were asymmetric, and publication bias was detected (SBP: *P* = 0.261, DBP: *P* < 0.001) (Supplementary Figures S15, S16).

### Blood glucose

A random effects model was used to pool glucose from 65 RCTs (3,413 participants) (Table 1), all of which required participants to fast for 8–14 h; the model revealed that PA was associated with a significant improvement in fasting glucose (*P* < 0.001) with high heterogeneity (WMD = −4.40; 95% CI: −5.44 to −3.36; I^2^ = 86.5%; P_heterogeneity_ < 0.001, random model). The asymmetry of the forest plot suggested high publication bias (P^Egger′s test^ = 0.838) (Supplementary Figure S21).

### Sensitivity analyses

Sensitivity analysis showed that all outcomes were stable for PA and SB.

## Discussion

This is the first meta-analysis to combine randomized controlled trials and longitudinal studies to assess the association between SB, PA, and CVD. The analysis of 148 RCTs and 36 longitudinal studies indicated that long-term PA improved indicators related to CVD risk in healthy adults and directly reduced the risk of CVD. Conversely, long-term SB increased the risk of CVD in healthy people, regardless of the study region, the gender of participants, and follow-up time. In addition, long-term PA was not associated with a large improvement in HDL in studies on older adult populations, studies on previously sedentary populations, studies with short-term interventions, or studies that assessed ME.

SB has been identified as a risk factor for CVD in several previous studies (43, 44). Our meta-analysis confirmed the association between SB and CVD (HR:1.34; 95% CI: 1.26 to 1.43; I^2^ = 52.3, *P* < 0.01; random model), suggesting a 34% increase in the risk of CVD (including non-fatal CHD, HF, and fatal myocardial infarction events) in people with frequent SB compared with those without SB. Evidence suggests that of the factors associated with all causes of CVD mortality, SB has the strongest association (8), and several other studies have reported strong, consistent results supporting this association. A prospective cohort study on 134,596 Americans found that the risk of death from CVD rose with increased sitting time, particularly while viewing television, and this finding was consistent across people of different weights, genders, and races; similar results were found in our study (45). A lack of PA due to SB leads to the decreased turnover of endogenous energy stores, myogenic glycogen, and intracellular lipids; in turn, these changes lead to skeletal muscle insulin resistance. When hyperinsulinism occurs, adipogenesis increases, promoting the production of very low-density lipoproteins and lower HDL levels in the liver. This results in the development of metabolic syndrome, which may contribute to the development of CVD. Similarly, steatosis may cause hyperglycemia, which not only causes diabetes but also potentially increases the risk of CVD (46, 47). In conclusion, the mechanisms through which SB causes CVD are complex and multifaceted.

We investigated the effects of long-term PA on CVD and several CVD risk indicators and found that long-term PA not only reduced the HR of CVD events (HR = 0.71; 95%CI: 0.66 to 0.77; I^2^ = 78.0%, P_heterogeneity_ < 0.001, random model) but also improved several CVD risk indicators. The cardioprotective effects of long-term PA have been confirmed by several studies. For example, an American cohort study that analyzed the effects of long-term PA in 88,140 adults found that the risk of CVD-specific death was 37 and 33% lower in participants who performed PA for 150–299 min and 1,500 min or more per week, respectively, compared with those who were inactive (48). Because of its large sample size, this study provides strong evidence supporting our meta-analysis results. For the mechanism by which PA reduces cardiovascular risk, one study suggests that PA improves cardiovascular health through the efficient use of energy-releasing raw materials (e.g., oxygen, fat, and glucose) and other resources, combined with autonomous skeletal muscle contraction that enhances metabolism and neural coordination (44). Furthermore, a large meta-analysis of RCTs showed that long-term PA significantly improved cardiopulmonary function and several CVD biomarkers (e.g., lipids) in healthy adults, which in turn reduced the incidence of CVD and improved cardiovascular health. To further explore the reasons for PA's effects on the risk of CVD, our study began by analyzing lipids because abnormalities in the lipoprotein–lipid profile account for 50% of the total risk of CVD (46); moreover, the assessment of lipid levels is the most common method to identify individuals at high risk of CVD (49). According to our study, long-term PA significantly reduced total TC, TGs, and LDL levels and significantly improved HDL levels. Other studies have shown that adults who engage in long-term PA have lower TC and LDL levels compared with those who are inactive (50, 51), these findings are consistent with our meta-analysis results. Furthermore, two longitudinal studies have demonstrated that TG levels are lower and HDL levels are higher in endurance athletes and in those with long-term aerobic training (52, 53). The more favorable lipid profiles in these populations may be due to an increase in lipoprotein lipase (LPL) mRNA, LPL mass, total LPL activity, and heparin-releasable LPL activity in the skeletal muscle after long-term PA (54). When the LPL concentration increases, the composition of lipoproteins changes *in vivo*, and apolipoprotein-E is redistributed. This change decreases plasma TG levels, LDL levels, and TC/HDL ratio and increases HDL concentrations (55, 56), thereby reducing the risk of CVD.

Furthermore, our study revealed significant improvements in BMI, blood pressure, and blood glucose in healthy adults who performed long-term PA, and these results have been widely confirmed. Several large RCTs have found that after more than 8 weeks of PA intervention, all participants had lower weight, blood pressure, and fasting glucose, and these effects were consistent for participants with different genders and weights as well as different types and intensities of PA (57–60). These improvements may be related to omentin-1, an adipokine that promotes insulin sensitivity; studies have shown that after long-term PA intervention, participants showed improvements in BMI, waist circumference, body fat, and blood glucose with a corresponding increase in their omentin-1 concentrations (61). Omentin-1 was present at lower concentrations in the obese population and was negatively correlated with BMI, fasting glucose, and blood pressure (62, 63). The concentration of omentin-1 in the body may have increased after the PA intervention, improving CVD-related outcomes.

In the subgroup analysis, we found that PA only significantly improved HDL when the intervention duration was longer than 12 weeks or the participants who were under 60 years of age. Shorter interventions may be insufficient to achieve observable improvements; moreover, the PA intensity may not reach the level needed for improvement in older adults because of their low exercise levels. In a previous study, a significant improvement in HDL was reported only after PA reached a certain intensity (64). We also observed a significant effect on HDL when people chose to perform AE or RE. Evidence shows that AE or RE alone can increase HDL levels (65), and the combination of AE and RE in ME can have the same effect (66); however, ME was not associated with significant changes in our study. This is likely because ME was mixed with other forms of exercise, such as balance exercises, and less evidence supports improvement in HDL with these forms of exercise; thus, the overall results may have been affected. Moreover, no significant differences were observed in terms of the effect of PA on HDL in the group with SB. This suggests that the beneficial effects of PA may be limited in people with SB; furthermore, a previous study showed that the effects of PA and SB were independent of each other (9). These findings warrant further investigation of the details of the interaction between PA and SB in future studies. In all other subgroup analyses, we obtained consistent results, indicating that the overall improvement in lipids levels was superior in participants who performed PA.

Our study has several implications in clinical practice and can provide guidance for practitioners. First, our findings support the American Heart Association (AHA) guidelines recommending long-term PA as a first-line treatment for improving dyslipidemia and reducing the risk of CVD (67). Therefore, 40 min of moderate to vigorous intensity aerobic exercise for three to four times per week is recommended to lower LDL and increase HDL (68). Second, our study found that PA also improved blood pressure levels, and according to the AHA guidelines, performing aerobic exercise three to four times a week for an average of 40 min per session for at least 12 weeks is a typical recommendation for improving blood pressure in adults (68). Third, according to our subgroup analysis, resistance training can also reduce the risk of CVD when performed at a recommended weekly training intensity of 500–1000 METs (69). In summary, patients should regularly engage in PA, and to maximize health benefits, they should perform at least 150 minutes of moderate-intensity aerobic exercise or 75 min of high-intensity aerobic exercise as well as two resistance training sessions per week (30).

## Strengths and limitations

This study is the first to combine RCTs and longitudinal studies to examine the association between PA, SB, and CVD, contributing new medical evidence to the literature. We not only distinguished the population but also considered factors such as economic status, year of publication, and the number of participants, making this a more in-depth study than previous work. Because the volume of literature included in the previous meta-analysis was not very large, we collected studies from larger databases, resulting in a larger sample size meeting the inclusion criteria. The results of this study may provide more treatment options to policymakers, clinicians, and caregivers, and the findings may help guide decision-making and facilitate more in-depth research in the future.

The present study also has several limitations. First, the uneven quality of the literature on RCTs may have impacted the overall quality of the studies, and the high risk of outcome assessment blinding created some heterogeneity and imprecision in the study results. In addition, the inclusion of longitudinal studies and RCTs may have resulted in inconsistent results. Furthermore, we did not analyze certain confounding factors, such as diet, smoking, and obesity, because of inconsistencies in implementation criteria, dosing criteria, and rubrics, which may have contributed to limitations in the outcomes. Considering the above limitations, the study results should be interpreted with caution.

## Conclusions

In conclusion, long-term SB increased the risk of CVD in healthy adults, whereas regular PA reduced the risk of CVD and improved indicators associated with CVD risk. However, improvement was limited in patients with SB, select ME and older age groups. Therefore, patients should be encouraged to limit their daily sitting time and increase their PA levels to reduce their risk of CVD. More detailed studies are needed to demonstrate and clarify the effects of PA and SB on CVD.

## Data availability statement

The original contributions presented in the study are included in the article/Supplementary material, further inquiries can be directed to the corresponding authors.

## Author contributions

Z-dL served as principal author and had full access to all the data in the study, takes responsibility for the accuracy of the data analysis, and the integrity of the data. Z-dL and MZ contributed to the conception, design, and drafting the manuscript. Z-dL, MZ, and C-zW contributed to data acquisition and interpretation. YY and J-hL contributed to revise of the article and final approval. All authors contributed to the article and approved the submitted version.

## Funding

The work was supported by the National Natural Science Youth Foundation of China (No. 32000830) and the Special funding in China Postdoctoral Fund Station (No. 2021T140356).

## Conflict of interest

The authors declare that the research was conducted in the absence of any commercial or financial relationships that could be construed as a potential conflict of interest.

## Publisher's note

All claims expressed in this article are solely those of the authors and do not necessarily represent those of their affiliated organizations, or those of the publisher, the editors and the reviewers. Any product that may be evaluated in this article, or claim that may be made by its manufacturer, is not guaranteed or endorsed by the publisher.

## References

[1] Olvera Lopez E Ballard BD Jan A. Cardiovascular Disease. StatPearls. Treasure Island (FL): StatPearls Publishing Copyright © 2022, StatPearls Publishing LLC. (2022).

[2] Townsend N Kazakiewicz D Lucy Wright F Timmis A Huculeci R Torbica A . Epidemiology of cardiovascular disease in Europe. Nature Rev Cardiol. (2022) 19:133–43. 10.1038/s41569-021-00607-3 34497402

[3] Zhou M Wang H Zhu J Chen W Wang L Liu S . Cause-specific mortality for 240 causes in China during 1990–2013: a systematic subnational analysis for the Global Burden of Disease Study 2013. Lancet. (2016) 387:251–72. 10.1016/S0140-6736(15)00551-6 26510778

[4] Liu S Li Y Zeng X Wang H Yin P Wang L . Burden of cardiovascular diseases in China, 1990-2016: findings from the 2016 Global Burden of Disease Study. JAMA Cardiol. (2019) 4:342–52. 10.1001/jamacardio.2019.0295 30865215 PMC6484795

[5] Lavie CJ Ozemek C Carbone S Katzmarzyk PT Blair SN. Sedentary behavior, exercise, and cardiovascular health. Circ Res. (2019) 124:799–815. 10.1161/CIRCRESAHA.118.312669 30817262

[6] Reiner Z Catapano AL De Backer G Graham I Taskinen MR Wiklund O . ESC/EAS Guidelines for the management of dyslipidaemias: the Task Force for the management of dyslipidaemias of the European Society of Cardiology (ESC) and the European Atherosclerosis Society (EAS). Eur Heart J. (2011) 32:1769–818. 10.1093/eurheartj/ehr158 21712404

[7] Fletcher GF Landolfo C Niebauer J Ozemek C Arena R Lavie CJ. Promoting physical activity and exercise: JACC health promotion series. J Am Coll Cardiol. (2018) 72:1622–39. 10.1016/j.jacc.2018.08.2141 30261965

[8] Katzmarzyk PT Powell KE Jakicic JM Troiano RP Piercy K Tennant B. Sedentary behavior and health: update from the 2018 physical activity guidelines advisory committee. Med Sci Sports Exerc. (2019) 51:1227–41. 10.1249/MSS.0000000000001935 31095080 PMC6527341

[9] Katzmarzyk PT Church TS Craig CL Bouchard C. Sitting time and mortality from all causes, cardiovascular disease, and cancer. Med Sci Sports Exerc. (2009) 41:998–1005. 10.1249/MSS.0b013e3181930355 19346988

[10] Cao Z Xu C Zhang P Wang Y. Associations of sedentary time and physical activity with adverse health conditions: outcome-wide analyses using isotemporal substitution model. EClinicalMed. (2022) 48:101424. 10.1016/j.eclinm.2022.101424 35516443 PMC9065298

[11] Aune D Schlesinger S Leitzmann MF Tonstad S Norat T Riboli E . Physical activity and the risk of heart failure: a systematic review and dose-response meta-analysis of prospective studies. Eur J Epidemiol. (2021) 36:367–81. 10.1007/s10654-020-00693-6 33331992 PMC8076120

[12] Caspersen CJ Powell KE Christenson GM. Physical activity, exercise, and physical fitness: definitions and distinctions for health-related research. Public Health Rep. (1985) 100:126–31. 3920711 PMC1424733

[13] Batrakoulis A Jamurtas AZ Metsios GS Perivoliotis K Liguori G Feito Y . Comparative efficacy of 5 exercise types on cardiometabolic health in overweight and obese adults: a systematic review and network meta-analysis of 81 randomized controlled trials. Circ Cardiovasc Qual Outcomes. (2022) 15:e008243. 10.1161/CIRCOUTCOMES.121.008243 35477256

[14] Oberoi S Robinson PD Cataudella D Culos-Reed SN Davis H Duong N . Physical activity reduces fatigue in patients with cancer and hematopoietic stem cell transplant recipients: a systematic review and meta-analysis of randomized trials. Crit Rev Oncol Hematol. (2018) 122:52–9. 10.1016/j.critrevonc.2017.12.011 29458789

[15] Nordengen S Andersen LB Solbraa AK Riiser A. Cycling is associated with a lower incidence of cardiovascular diseases and death: part 1 - systematic review of cohort studies with meta-analysis. Br J Sports Med. (2019) 53:870–8. 10.1136/bjsports-2018-099099 31151937

[16] Zhuo C Zhao J Chen M Lu Y. Physical activity and risks of cardiovascular diseases: a mendelian randomization study. Front Cardiovasc Med. (2021) 8:722154. 10.3389/fcvm.2021.722154 34660723 PMC8511639

[17] Caterini JE Campisi ES Cifra B. Physical activity promotion in pediatric congenital heart disease: are we running late? Can J Cardiol. (2020) 36:1406–16. 10.1016/j.cjca.2020.07.003 32673643

[18] Recchioni R Marcheselli F Antonicelli R Mensà E Lazzarini R Procopio AD . Epigenetic effects of physical activity in elderly patients with cardiovascular disease. Exp Gerontol. (2017) 100:17–27. 10.1016/j.exger.2017.10.016 29074290

[19] Cheng W Zhang Z Cheng W Yang C Diao L Liu W. Associations of leisure-time physical activity with cardiovascular mortality: a systematic review and meta-analysis of 44 prospective cohort studies. Eur J Prev Cardiol. (2018) 25:1864–72. 10.1177/2047487318795194 30157685

[20] Ekelund U Steene-Johannessen J Brown WJ Fagerland MW Owen N Powell KE . Does physical activity attenuate, or even eliminate, the detrimental association of sitting time with mortality? A harmonised meta-analysis of data from more than 1 million men and women. Lancet. (2016) 388:1302–10. 10.1016/S0140-6736(16)30370-1 27475271

[21] Wahid A Manek N Nichols M Kelly P Foster C Webster P . Quantifying the association between physical activity and cardiovascular disease and diabetes: a systematic review and meta-analysis. J Am Heart Assoc. (2016) 5:e2495 10.1161/JAHA.115.002495 27628572 PMC5079002

[22] Lin X Zhang X Guo J Roberts CK McKenzie S Wu WC . Effects of exercise training on cardiorespiratory fitness and biomarkers of cardiometabolic health: a systematic review and meta-analysis of randomized controlled trials. J Am Heart Assoc. (2015) 4:e2014 10.1161/JAHA.115.002014 26116691 PMC4608087

[23] Patterson R McNamara E Tainio M de Sá TH Smith AD Sharp SJ . Sedentary behaviour and risk of all-cause, cardiovascular and cancer mortality, and incident type 2 diabetes: a systematic review and dose response meta-analysis. Eur J Epidemiol. (2018) 33:811–29. 10.1007/s10654-018-0380-1 29589226 PMC6133005

[24] Oja P Kelly P Murtagh EM Murphy MH Foster C Titze S. Effects of frequency, intensity, duration and volume of walking interventions on CVD risk factors: a systematic review and meta-regression analysis of randomised controlled trials among inactive healthy adults. Br J Sports Med. (2018) 52:769–75. 10.1136/bjsports-2017-098558 29858464

[25] Lee YK Cho SY Roh HT. Effects of 16 weeks of taekwondo training on the cerebral blood flow velocity, circulating neurotransmitters, and subjective well-being of obese postmenopausal women. Int J Environ Res Public Health. (2021) 18:e82010789. 10.3390/ijerph182010789 34682534 PMC8535195

[26] Mu X Yu K Long P Niu R Li W Chen H . Leisure-time physical activity and risk of incident cardiovascular disease in Chinese retired adults. Sci Rep. (2021) 11:24202. 10.1038/s41598-021-03475-6 34921190 PMC8683485

[27] Higgins JP Thomas J Chandler J Cumpston M Li T Page MJ . Cochrane Handbook for Systematic Reviews of Interventions. New York: John Wiley & Sons (2019). 10.1002/14651858.ED000142 PMC10284251 31643080

[28] Stroup DF Berlin JA Morton SC Olkin I Williamson GD Rennie D . Meta-analysis of observational studies in epidemiology: a proposal for reporting. Meta-analysis Of Observational Studies in Epidemiology (MOOSE) group. JAMA. (2000) 283:2008–12. 10.1001/jama.283.15.2008 10789670

[29] Garber CE Blissmer B Deschenes MR Franklin BA Lamonte MJ Lee IM . American College of Sports Medicine position stand. Quantity and quality of exercise for developing and maintaining cardiorespiratory, musculoskeletal, and neuromotor fitness in apparently healthy adults: guidance for prescribing exercise. Med Sci Sports Exerc. (2011) 43:1334–59. 10.1249/MSS.0b013e318213fefb 21694556

[30] Services USDoHaH. Physical Activity Guidelines for Americans. 2nd edition. (2018). Available online at: https://health.gov/sites/default/files/2019-09/Physical_Activity_Guidelines_2nd_edition.pdf (accessed September, 2019).

[31] Wang S Yin H Wang X Jia Y Wang C Wang L . Efficacy of different types of exercises on global cognition in adults with mild cognitive impairment: a network meta-analysis. Aging Clin Exp Res. (2019) 31:1391–400. 10.1007/s40520-019-01142-5 30739298

[32] Higgins JP. Cochrane Handbook for Systematic Reviews of Interventions Version 5.0. 2. (2009). The Cochrane Collaboration. Available online at: https://training.cochrane.org/handbook/archive/v5.0.2/.

[33] Stang A. Critical evaluation of the Newcastle-Ottawa scale for the assessment of the quality of nonrandomized studies in meta-analyses. Eur J Epidemiol. (2010) 25:603–5. 10.1007/s10654-010-9491-z 20652370

[34] Abou-Setta AM Mousavi SS Spooner C Schouten JR Pasichnyk D Armijo-Olivo S . Newcastle-Ottawa Scale Assessment of Cohort Studies: Newcastle-Ottawa Scale Assessment of Cohort Studies. Agency for Healthcare Research and Quality (US). (2012). 23035275

[35] Hedges LV. Distribution theory for Glass's estimator of effect size and related estimators. J Educat Statis. (1981) 6:107–28. 10.3102/10769986006002107

[36] Wan X Wang W Liu J Tong T. Estimating the sample mean and standard deviation from the sample size, median, range and/or interquartile range. BMC Med Res Methodol. (2014) 14:135. 10.1186/1471-2288-14-135 25524443 PMC4383202

[37] Borenstein M Hedges LV Higgins JP Rothstein HR A. basic introduction to fixed-effect and random-effects models for meta-analysis. Res Synth Methods. (2010) 1:97–111. 10.1002/jrsm.12 26061376

[38] Higgins JP Thompson SG. Quantifying heterogeneity in a meta-analysis. Stat Med. (2002) 21:1539–58. 10.1002/sim.1186 12111919

[39] Higgins JP Thompson SG Deeks JJ Altman DG. Measuring inconsistency in meta-analyses. Bmj. (2003) 327:557–60. 10.1136/bmj.327.7414.557 12958120 PMC192859

[40] Egger M Juni P Bartlett C Holenstein F Sterne J. How important are comprehensive literature searches and the assessment of trial quality in systematic reviews? Empirical study. Health Technol Assess. (2003) 7:1–76. 10.3310/hta7010 12583822

[41] Korshøj M Ravn MH Holtermann A Hansen Å M Krustrup P. Aerobic exercise reduces biomarkers related to cardiovascular risk among cleaners: effects of a worksite intervention RCT. Int Arch Occup Environ Health. (2016) 89:239–49. 10.1007/s00420-015-1067-5 26139093 PMC4724374

[42] Dagistan Akgöz A Gözüm S. Effectiveness of a nurse-led physical activity intervention to decrease cardiovascular disease risk in middle-aged adults: a pilot randomized controlled study. J Vascular Nurs. (2020) 38:140–8. 10.1016/j.jvn.2020.05.002 32950115

[43] Young DR Hivert MF Alhassan S Camhi SM Ferguson JF Katzmarzyk PT . Sedentary behavior and cardiovascular morbidity and mortality: a science advisory from the American Heart Association. Circulation. (2016) 134:e262–79. 10.1161/CIR.0000000000000440 27528691

[44] Powell KE Paluch AE Blair SN. Physical activity for health: What kind? How much? How intense? On top of what? Annual Rev Public Health. (2011) 32:349–65. 10.1146/annurev-publhealth-031210-101151 21128761

[45] Kim Y Wilkens LR Park SY Goodman MT Monroe KR Kolonel LN. Association between various sedentary behaviours and all-cause, cardiovascular disease and cancer mortality: the Multiethnic Cohort Study. Int J Epidemiol. (2013) 42:1040–56. 10.1093/ije/dyt108 24062293 PMC3781003

[46] Booth FW Laye MJ Lees SJ Rector RS Thyfault JP. Reduced physical activity and risk of chronic disease: the biology behind the consequences. Eur J Appl Physiol. (2008) 102:381–90. 10.1007/s00421-007-0606-5 17987311

[47] Leiter LA. From hyperglycemia to the risk of cardiovascular disease. Rev Cardiovasc Med. (2006) 7 (Suppl 2):S3–9. Available online at: https://www.imrpress.com/journal/RCM/7/S2/pii/1561344059777-518357461?utm_source=TrendMD&utm_medium=cpc&utm_campaign=Reviews_in_Cardiovascular_Medicine_TrendMD_0 17224875

[48] Zhao M Veeranki SP Li S Steffen LM Xi B. Beneficial associations of low and large doses of leisure time physical activity with all-cause, cardiovascular disease and cancer mortality: a national cohort study of 88,140 US adults. Br J Sports Med. (2019) 53:1405–11. 10.1136/bjsports-2018-099254 30890520

[49] Arsenault BJ Boekholdt SM Kastelein JJ. Lipid parameters for measuring risk of cardiovascular disease. Nature Rev Cardiol. (2011) 8:197–206. 10.1038/nrcardio.2010.223 21283149

[50] Hartung GH Foreyt JP Mitchell RE Vlasek I Gotto AM. Jr.. Relation of diet to high-density-lipoprotein cholesterol in middle-aged marathon runners, joggers, and inactive me. New Eng J Med. (1980) 302:357–61. 10.1056/NEJM198002143020701 7351926

[51] Nagay B Zielinski T Lawczynski L. [Dermal calcinosis treated surgically]. Wiadomosci lekarskie. (1977) 30:493–4. 848025

[52] Driskell JA Wolinsky I. Energy-Yielding Macronutrients and Energy Metabolism In Sports Nutrition. Florida: CRC Press. (1999).

[53] Durstine JL Haskell WL. Effects of exercise training on plasma lipids and lipoproteins. Exerc Sport Sci Rev. (1994) 22:477–521. 10.1249/00003677-199401000-00017 7925552

[54] Hamilton MT Etienne J McClure WC Pavey BS Holloway AK. Role of local contractile activity and muscle fiber type on LPL regulation during exercise. Am J Physiol. (1998) 275:E1016–22. 10.1152/ajpendo.1998.275.6.E1016 9843744

[55] Liu MS Jirik FR LeBoeuf RC Henderson H Castellani LW Lusis AJ . Alteration of lipid profiles in plasma of transgenic mice expressing human lipoprotein lipase. J Biol Chem. (1994) 269:11417–24. 10.1016/S0021-9258(19)78140-X 8157673

[56] Oscai LB Tsika RW Essig DA. Exercise training has a heparin-like effect on lipoprotein lipase activity in muscle. Can J Physiol Pharmacol. (1992) 70:905–9. 10.1139/y92-121 1423034

[57] Trajković N Sporiš G Krističević T Bogataj Š. Effects of small-sided recreational volleyball on health markers and physical fitness in middle-aged men. Int J Environ Res Public Health. (2020) 17:93021 10.3390/ijerph17093021 32349223 PMC7246439

[58] Connolly LJ Scott S Morencos CM Fulford J Jones AM Knapp K . Impact of a novel home-based exercise intervention on health indicators in inactive premenopausal women: a 12-week randomised controlled trial. Eur J Appl Physiol. (2020) 120:771–82. 10.1007/s00421-020-04315-7 32193660

[59] Chen N Xia X Qin L Luo L Han S Wang G . Effects of 8-week hatha yoga training on metabolic and inflammatory markers in healthy, female chinese subjects: a randomized clinical trial. Biomed Res Int. (2016) 2016:5387258. 10.1155/2016/5387258 27563670 PMC4987461

[60] Dalleck LC Allen BA Hanson BA Borresen EC Erickson ME De Lap SL. Dose-response relationship between moderate-intensity exercise duration and coronary heart disease risk factors in postmenopausal women. J Women's Health. (2002). (2009) 18:105–13 10.1089/jwh.2008.0790 19132882

[61] Saremi A Asghari M Ghorbani A. Effects of aerobic training on serum omentin-1 and cardiometabolic risk factors in overweight and obese men. J Sports Sci. (2010) 28:993–8. 10.1080/02640414.2010.484070 20544489

[62] de Souza Batista CM Yang RZ Lee MJ Glynn NM Yu DZ Pray J . Omentin plasma levels and gene expression are decreased in obesity. Diabetes. (2007) 56:1655–61. 10.2337/db06-1506 17329619

[63] Tan BK Adya R Farhatullah S Lewandowski KC O'Hare P Lehnert H . Omentin-1, a novel adipokine, is decreased in overweight insulin-resistant women with polycystic ovary syndrome: ex vivo and in vivo regulation of omentin-1 by insulin and glucose. Diabetes. (2008) 57:801–8. 10.2337/db07-0990 18174521

[64] Kokkinos PF Holland JC Narayan P Colleran JA Dotson CO Papademetriou V. Miles run per week and high-density lipoprotein cholesterol levels in healthy, middle-aged men: a dose-response relationship. Arch Intern Med. (1995) 155:415–20. 10.1001/archinte.1995.00430040091011 7848025

[65] Potteiger JA Claytor RP Hulver MW Hughes MR Carper MJ Richmond S . Resistance exercise and aerobic exercise when paired with dietary energy restriction both reduce the clinical components of metabolic syndrome in previously physically inactive males. Eur J Appl Physiol. (2012) 112:2035–44. 10.1007/s00421-011-2174-y 21947428

[66] Woudberg NJ Mendham AE Katz AA Goedecke JH Lecour S. Exercise intervention alters HDL subclass distribution and function in obese women. Lipids Health Dis. (2018) 17:232. 10.1186/s12944-018-0879-1 30301473 PMC6178267

[67] Grundy SM Stone NJ Bailey AL Beam C Birtcher KK Blumenthal RS . 2018 AHA/ACC/AACVPR/AAPA/ABC/ACPM/ADA/AGS/APhA/ASPC/NLA/PCNA Guideline on the Management of Blood Cholesterol: A Report of the American College of Cardiology/American Heart Association Task Force on Clinical Practice Guidelines. J Am Coll Cardiol. (2019) 73:e285–350. 10.1016/j.jacc.2018.11.003 30423393

[68] Eckel RH Jakicic JM Ard JD de Jesus JM Houston Miller N Hubbard VS . 2013 AHA/ACC guideline on lifestyle management to reduce cardiovascular risk: a report of the American College of Cardiology/American Heart Association Task Force on Practice Guidelines. J Am Coll Cardiol. (2014) 63:2960–84 10.1161/01.cir.0000437740.48606.d1 24239922

[69] Jeong SW Kim SH Kang SH Kim HJ Yoon CH Youn TJ . Mortality reduction with physical activity in patients with and without cardiovascular disease. Eur Heart J. (2019) 40:3547–55. 10.1093/eurheartj/ehz564 31504416 PMC6855138

