# Supplementary material for: Association between sedentary behavior, physical activity, and cardiovascular disease-related outcomes in adults—A meta-analysis and systematic review
Source: Front Public Health. 2022 Oct 19;10:1018460. doi: 10.3389/fpubh.2022.1018460 (PMC9632849; doi:10.3389/fpubh.2022.1018460)
Supplement: Supplementary file 1 [file Data_Sheet_1.DOCX]

**Association between sedentary behavior, physical activity and cardiovascular disease-related outcomes in adults —— A meta-analysis and systematic review**

**Appendix**

Contents

[Search strategies 1](#_Toc113487005)

[PubMed 1](#_Toc113487006)

[Embase 10](#_Toc113487007)

[Cochrane 17](#_Toc113487008)

[Web of Science 18](#_Toc113487009)

[Literature quality assessment 20](#_Toc113487010)

[Randomized controlled trials 20](#_Toc113487011)

[Longitudinal studies 22](#_Toc113487012)

[Forest plot 23](#_Toc113487013)

[Randomized controlled trials 23](#_Toc113487014)

[Longitudinal studies 31](#_Toc113487015)

[Funnel plot 33](#_Toc113487016)

[Randomized controlled trials 33](#_Toc113487017)

[Longitudinal studies 37](#_Toc113487018)

[PRISMA 2020 Checklist 38](#_Toc113487019)

[PRISMA 2020 for Abstracts checklist 41](#_Toc113487020)

[List of literature included in the Meta-analysis 43](#_Toc113487021)

# Search strategies

## PubMed

#1 "Cardiovascular Diseases"[Mesh]

#2 ((Cardiovascular Disease[Title/Abstract]) OR (Disease, Cardiovascular[Title/Abstract])) OR (Diseases, Cardiovascular[Title/Abstract])

#3 ("Cardiovascular Diseases"[Mesh]) OR (((Cardiovascular Disease[Title/Abstract]) OR (Disease, Cardiovascular[Title/Abstract])) OR (Diseases, Cardiovascular[Title/Abstract]))

#4 "Exercise"[Mesh]

#5 (((((((((((((((((((Physical Activity[Title/Abstract]) OR (Activities, Physical[Title/Abstract])) OR (Activity, Physical[Title/Abstract])) OR (Physical Activities[Title/Abstract])) OR (Exercise, Physical[Title/Abstract])) OR (Exercises, Physical[Title/Abstract])) OR (Physical Exercise[Title/Abstract])) OR (Physical Exercises[Title/Abstract])) OR (Exercise, Isometric[Title/Abstract])) OR (Exercises, Isometric[Title/Abstract])) OR (Isometric Exercises[Title/Abstract])) OR (Isometric Exercise[Title/Abstract])) OR (Exercise, Aerobic[Title/Abstract])) OR (Aerobic Exercise[Title/Abstract])) OR (Aerobic Exercises[Title/Abstract])) OR (Exercises, Aerobic[Title/Abstract])) OR (Exercise Training[Title/Abstract])) OR (Exercise Trainings[Title/Abstract])) OR (Training, Exercise[Title/Abstract])) OR (Trainings, Exercise[Title/Abstract])

#6 ("Exercise"[Mesh]) OR ((((((((((((((((((((Physical Activity[Title/Abstract]) OR (Activities, Physical[Title/Abstract])) OR (Activity, Physical[Title/Abstract])) OR (Physical Activities[Title/Abstract])) OR (Exercise, Physical[Title/Abstract])) OR (Exercises, Physical[Title/Abstract])) OR (Physical Exercise[Title/Abstract])) OR (Physical Exercises[Title/Abstract])) OR (Exercise, Isometric[Title/Abstract])) OR (Exercises, Isometric[Title/Abstract])) OR (Isometric Exercises[Title/Abstract])) OR (Isometric Exercise[Title/Abstract])) OR (Exercise, Aerobic[Title/Abstract])) OR (Aerobic Exercise[Title/Abstract])) OR (Aerobic Exercises[Title/Abstract])) OR (Exercises, Aerobic[Title/Abstract])) OR (Exercise Training[Title/Abstract])) OR (Exercise Trainings[Title/Abstract])) OR (Training, Exercise[Title/Abstract])) OR (Trainings, Exercise[Title/Abstract]))

#7 "Sedentary Behavior"[Mesh]

#8 (((((((((Behavior, Sedentary[Title/Abstract]) OR (Sedentary Behaviors[Title/Abstract])) OR (Sedentary Lifestyle[Title/Abstract])) OR (Lifestyle, Sedentary[Title/Abstract])) OR (Physical Inactivity[Title/Abstract])) OR (Inactivity, Physical[Title/Abstract])) OR (Lack of Physical Activity[Title/Abstract])) OR (Sedentary Time[Title/Abstract])) OR (Sedentary Times[Title/Abstract])) OR (Time, Sedentary[Title/Abstract])

#9 ("Sedentary Behavior"[Mesh]) OR ((((((((((Behavior, Sedentary[Title/Abstract]) OR (Sedentary Behaviors[Title/Abstract])) OR (Sedentary Lifestyle[Title/Abstract])) OR (Lifestyle, Sedentary[Title/Abstract])) OR (Physical Inactivity[Title/Abstract])) OR (Inactivity, Physical[Title/Abstract])) OR (Lack of Physical Activity[Title/Abstract])) OR (Sedentary Time[Title/Abstract])) OR (Sedentary Times[Title/Abstract])) OR (Time, Sedentary[Title/Abstract]))

#10 cohort studies[mesh:noexp] OR longitudinal studies[mesh:noexp] OR follow-up studies[mesh:noexp] OR prospective studies[mesh:noexp] OR retrospective studies[mesh:noexp] OR cohort[TIAB] OR longitudinal[TIAB] OR prospective[TIAB] OR retrospective[TIAB]

#11 "Adult"[Mesh]

#12 adults[Title/Abstract]

#13 ("Adult"[Mesh]) OR (adults[Title/Abstract])

#14 (((("Cardiovascular Diseases"[Mesh]) OR (((Cardiovascular Disease[Title/Abstract]) OR (Disease, Cardiovascular[Title/Abstract])) OR (Diseases, Cardiovascular[Title/Abstract]))) AND (("Exercise"[Mesh]) OR ((((((((((((((((((((Physical Activity[Title/Abstract]) OR (Activities, Physical[Title/Abstract])) OR (Activity, Physical[Title/Abstract])) OR (Physical Activities[Title/Abstract])) OR (Exercise, Physical[Title/Abstract])) OR (Exercises, Physical[Title/Abstract])) OR (Physical Exercise[Title/Abstract])) OR (Physical Exercises[Title/Abstract])) OR (Exercise, Isometric[Title/Abstract])) OR (Exercises, Isometric[Title/Abstract])) OR (Isometric Exercises[Title/Abstract])) OR (Isometric Exercise[Title/Abstract])) OR (Exercise, Aerobic[Title/Abstract])) OR (Aerobic Exercise[Title/Abstract])) OR (Aerobic Exercises[Title/Abstract])) OR (Exercises, Aerobic[Title/Abstract])) OR (Exercise Training[Title/Abstract])) OR (Exercise Trainings[Title/Abstract])) OR (Training, Exercise[Title/Abstract])) OR (Trainings, Exercise[Title/Abstract])))) AND (cohort studies[mesh:noexp] OR longitudinal studies[mesh:noexp] OR follow-up studies[mesh:noexp] OR prospective studies[mesh:noexp] OR retrospective studies[mesh:noexp] OR cohort[TIAB] OR longitudinal[TIAB] OR prospective[TIAB] OR retrospective[TIAB])) AND (("Adult"[Mesh]) OR (adults[Title/Abstract]))

#15 (((("Cardiovascular Diseases"[Mesh]) OR (((Cardiovascular Disease[Title/Abstract]) OR (Disease, Cardiovascular[Title/Abstract])) OR (Diseases, Cardiovascular[Title/Abstract]))) AND (("Sedentary Behavior"[Mesh]) OR ((((((((((Behavior, Sedentary[Title/Abstract]) OR (Sedentary Behaviors[Title/Abstract])) OR (Sedentary Lifestyle[Title/Abstract])) OR (Lifestyle, Sedentary[Title/Abstract])) OR (Physical Inactivity[Title/Abstract])) OR (Inactivity, Physical[Title/Abstract])) OR (Lack of Physical Activity[Title/Abstract])) OR (Sedentary Time[Title/Abstract])) OR (Sedentary Times[Title/Abstract])) OR (Time, Sedentary[Title/Abstract])))) AND (cohort studies[mesh:noexp] OR longitudinal studies[mesh:noexp] OR follow-up studies[mesh:noexp] OR prospective studies[mesh:noexp] OR retrospective studies[mesh:noexp] OR cohort[TIAB] OR longitudinal[TIAB] OR prospective[TIAB] OR retrospective[TIAB])) AND (("Adult"[Mesh]) OR (adults[Title/Abstract]))

#16 "Body Mass Index"[Mesh]

#17 ((((Index, Body Mass[Title/Abstract]) OR (Quetelet Index[Title/Abstract])) OR (Index, Quetelet[Title/Abstract])) OR (Quetelet's Index[Title/Abstract])) OR (Quetelets Index[Title/Abstract])

#18 ("Body Mass Index"[Mesh]) OR (((((Index, Body Mass[Title/Abstract]) OR (Quetelet Index[Title/Abstract])) OR (Index, Quetelet[Title/Abstract])) OR (Quetelet's Index[Title/Abstract])) OR (Quetelets Index[Title/Abstract]))

#19 "Blood Pressure"[Mesh]

#20 (((((((Pressure, Blood[Title/Abstract]) OR (Diastolic Pressure[Title/Abstract])) OR (Pressure, Diastolic[Title/Abstract])) OR (Pulse Pressure[Title/Abstract])) OR (Pressure, Pulse[Title/Abstract])) OR (Systolic Pressure[Title/Abstract])) OR (Pressure, Systolic[Title/Abstract])) OR (Pressures, Systolic[Title/Abstract])#21

#21 ("Blood Pressure"[Mesh]) OR ((((((((Pressure, Blood[Title/Abstract]) OR (Diastolic Pressure[Title/Abstract])) OR (Pressure, Diastolic[Title/Abstract])) OR (Pulse Pressure[Title/Abstract])) OR (Pressure, Pulse[Title/Abstract])) OR (Systolic Pressure[Title/Abstract])) OR (Pressure, Systolic[Title/Abstract])) OR (Pressures, Systolic[Title/Abstract]))

#22 "Blood Glucose"[Mesh]

#23 ((Blood Sugar[Title/Abstract]) OR (Sugar, Blood[Title/Abstract])) OR (Glucose, Blood[Title/Abstract])

#24 ("Blood Glucose"[Mesh]) OR (((Blood Sugar[Title/Abstract]) OR (Sugar, Blood[Title/Abstract])) OR (Glucose, Blood[Title/Abstract]))

#25 "Triglycerides"[Mesh]

#26 ((Triacylglycerols[Title/Abstract]) OR (Triacylglycerol[Title/Abstract])) OR (Triglyceride[Title/Abstract])

#27 ("Triglycerides"[Mesh]) OR (((Triacylglycerols[Title/Abstract]) OR (Triacylglycerol[Title/Abstract])) OR (Triglyceride[Title/Abstract]))

#28 "Cholesterol"[Mesh]

#29 Epicholesterol[Title/Abstract]

#30 ("Cholesterol"[Mesh]) OR (Epicholesterol[Title/Abstract])

#31 "Lipoproteins, HDL"[Mesh]

#32 (((((((((((((((HDL Lipoproteins[Title/Abstract]) OR (High-Density Lipoprotein[Title/Abstract])) OR (Lipoprotein, High-Density[Title/Abstract])) OR (High-Density Lipoproteins[Title/Abstract])) OR (High Density Lipoproteins[Title/Abstract])) OR (Lipoproteins, High-Density[Title/Abstract])) OR (alpha-Lipoproteins[Title/Abstract])) OR (alpha Lipoproteins[Title/Abstract])) OR (Heavy Lipoproteins[Title/Abstract])) OR (Lipoproteins, Heavy[Title/Abstract])) OR (High Density Lipoprotein[Title/Abstract])) OR (Density Lipoprotein, High[Title/Abstract])) OR (Lipoprotein, High Density[Title/Abstract])) OR (alpha-Lipoprotein[Title/Abstract])) OR (alpha Lipoprotein[Title/Abstract])) OR (alpha-1 Lipoprotein[Title/Abstract])

#33 ("Lipoproteins, HDL"[Mesh]) OR ((((((((((((((((HDL Lipoproteins[Title/Abstract]) OR (High-Density Lipoprotein[Title/Abstract])) OR (Lipoprotein, High-Density[Title/Abstract])) OR (High-Density Lipoproteins[Title/Abstract])) OR (High Density Lipoproteins[Title/Abstract])) OR (Lipoproteins, High-Density[Title/Abstract])) OR (alpha-Lipoproteins[Title/Abstract])) OR (alpha Lipoproteins[Title/Abstract])) OR (Heavy Lipoproteins[Title/Abstract])) OR (Lipoproteins, Heavy[Title/Abstract])) OR (High Density Lipoprotein[Title/Abstract])) OR (Density Lipoprotein, High[Title/Abstract])) OR (Lipoprotein, High Density[Title/Abstract])) OR (alpha-Lipoprotein[Title/Abstract])) OR (alpha Lipoprotein[Title/Abstract])) OR (alpha-1 Lipoprotein[Title/Abstract]))

#34 "Lipoproteins, LDL"[Mesh]

#35 ((((((((((((((((((LDL Lipoproteins[Title/Abstract]) OR (beta-Lipoprotein[Title/Abstract])) OR (beta Lipoprotein[Title/Abstract])) OR (Low-Density Lipoproteins[Title/Abstract])) OR (Lipoproteins, Low-Density[Title/Abstract])) OR (Low Density Lipoproteins[Title/Abstract])) OR (beta-Lipoproteins[Title/Abstract])) OR (beta Lipoproteins[Title/Abstract])) OR (Low-Density Lipoprotein[Title/Abstract])) OR (Lipoprotein, Low-Density[Title/Abstract])) OR (Low Density Lipoprotein[Title/Abstract])) OR (LDL-2[Title/Abstract])) OR (LDL2[Title/Abstract])) OR (Low-Density Lipoprotein 2[Title/Abstract])) OR (Low Density Lipoprotein 2[Title/Abstract])) OR (LDL-1[Title/Abstract])) OR (LDL1[Title/Abstract])) OR (Low-Density Lipoprotein 1[Title/Abstract])) OR (Low Density Lipoprotein 1[Title/Abstract])

#36 ("Lipoproteins, LDL"[Mesh]) OR (((((((((((((((((((LDL Lipoproteins[Title/Abstract]) OR (beta-Lipoprotein[Title/Abstract])) OR (beta Lipoprotein[Title/Abstract])) OR (Low-Density Lipoproteins[Title/Abstract])) OR (Lipoproteins, Low-Density[Title/Abstract])) OR (Low Density Lipoproteins[Title/Abstract])) OR (beta-Lipoproteins[Title/Abstract])) OR (beta Lipoproteins[Title/Abstract])) OR (Low-Density Lipoprotein[Title/Abstract])) OR (Lipoprotein, Low-Density[Title/Abstract])) OR (Low Density Lipoprotein[Title/Abstract])) OR (LDL-2[Title/Abstract])) OR (LDL2[Title/Abstract])) OR (Low-Density Lipoprotein 2[Title/Abstract])) OR (Low Density Lipoprotein 2[Title/Abstract])) OR (LDL-1[Title/Abstract])) OR (LDL1[Title/Abstract])) OR (Low-Density Lipoprotein 1[Title/Abstract])) OR (Low Density Lipoprotein 1[Title/Abstract]))

#37 ((((((("Body Mass Index"[Mesh]) OR (((((Index, Body Mass[Title/Abstract]) OR (Quetelet Index[Title/Abstract])) OR (Index, Quetelet[Title/Abstract])) OR (Quetelet's Index[Title/Abstract])) OR (Quetelets Index[Title/Abstract]))) OR (("Blood Pressure"[Mesh]) OR ((((((((Pressure, Blood[Title/Abstract]) OR (Diastolic Pressure[Title/Abstract])) OR (Pressure, Diastolic[Title/Abstract])) OR (Pulse Pressure[Title/Abstract])) OR (Pressure, Pulse[Title/Abstract])) OR (Systolic Pressure[Title/Abstract])) OR (Pressure, Systolic[Title/Abstract])) OR (Pressures, Systolic[Title/Abstract])))) OR (("Blood Glucose"[Mesh]) OR (((Blood Sugar[Title/Abstract]) OR (Sugar, Blood[Title/Abstract])) OR (Glucose, Blood[Title/Abstract])))) OR (("Triglycerides"[Mesh]) OR (((Triacylglycerols[Title/Abstract]) OR (Triacylglycerol[Title/Abstract])) OR (Triglyceride[Title/Abstract])))) OR (("Cholesterol"[Mesh]) OR (Epicholesterol[Title/Abstract]))) OR (("Lipoproteins, HDL"[Mesh]) OR ((((((((((((((((HDL Lipoproteins[Title/Abstract]) OR (High-Density Lipoprotein[Title/Abstract])) OR (Lipoprotein, High-Density[Title/Abstract])) OR (High-Density Lipoproteins[Title/Abstract])) OR (High Density Lipoproteins[Title/Abstract])) OR (Lipoproteins, High-Density[Title/Abstract])) OR (alpha-Lipoproteins[Title/Abstract])) OR (alpha Lipoproteins[Title/Abstract])) OR (Heavy Lipoproteins[Title/Abstract])) OR (Lipoproteins, Heavy[Title/Abstract])) OR (High Density Lipoprotein[Title/Abstract])) OR (Density Lipoprotein, High[Title/Abstract])) OR (Lipoprotein, High Density[Title/Abstract])) OR (alpha-Lipoprotein[Title/Abstract])) OR (alpha Lipoprotein[Title/Abstract])) OR (alpha-1 Lipoprotein[Title/Abstract])))) OR (("Lipoproteins, LDL"[Mesh]) OR (((((((((((((((((((LDL Lipoproteins[Title/Abstract]) OR (beta-Lipoprotein[Title/Abstract])) OR (beta Lipoprotein[Title/Abstract])) OR (Low-Density Lipoproteins[Title/Abstract])) OR (Lipoproteins, Low-Density[Title/Abstract])) OR (Low Density Lipoproteins[Title/Abstract])) OR (beta-Lipoproteins[Title/Abstract])) OR (beta Lipoproteins[Title/Abstract])) OR (Low-Density Lipoprotein[Title/Abstract])) OR (Lipoprotein, Low-Density[Title/Abstract])) OR (Low Density Lipoprotein[Title/Abstract])) OR (LDL-2[Title/Abstract])) OR (LDL2[Title/Abstract])) OR (Low-Density Lipoprotein 2[Title/Abstract])) OR (Low Density Lipoprotein 2[Title/Abstract])) OR (LDL-1[Title/Abstract])) OR (LDL1[Title/Abstract])) OR (Low-Density Lipoprotein 1[Title/Abstract])) OR (Low Density Lipoprotein 1[Title/Abstract])))

#38 randomized controlled trial[Publication Type] OR randomized[Title/Abstract] OR placebo[Title/Abstract]

#39 (((((((((("Body Mass Index"[Mesh]) OR (((((Index, Body Mass[Title/Abstract]) OR (Quetelet Index[Title/Abstract])) OR (Index, Quetelet[Title/Abstract])) OR (Quetelet's Index[Title/Abstract])) OR (Quetelets Index[Title/Abstract]))) OR (("Blood Pressure"[Mesh]) OR ((((((((Pressure, Blood[Title/Abstract]) OR (Diastolic Pressure[Title/Abstract])) OR (Pressure, Diastolic[Title/Abstract])) OR (Pulse Pressure[Title/Abstract])) OR (Pressure, Pulse[Title/Abstract])) OR (Systolic Pressure[Title/Abstract])) OR (Pressure, Systolic[Title/Abstract])) OR (Pressures, Systolic[Title/Abstract])))) OR (("Blood Glucose"[Mesh]) OR (((Blood Sugar[Title/Abstract]) OR (Sugar, Blood[Title/Abstract])) OR (Glucose, Blood[Title/Abstract])))) OR (("Triglycerides"[Mesh]) OR (((Triacylglycerols[Title/Abstract]) OR (Triacylglycerol[Title/Abstract])) OR (Triglyceride[Title/Abstract])))) OR (("Cholesterol"[Mesh]) OR (Epicholesterol[Title/Abstract]))) OR (("Lipoproteins, HDL"[Mesh]) OR ((((((((((((((((HDL Lipoproteins[Title/Abstract]) OR (High-Density Lipoprotein[Title/Abstract])) OR (Lipoprotein, High-Density[Title/Abstract])) OR (High-Density Lipoproteins[Title/Abstract])) OR (High Density Lipoproteins[Title/Abstract])) OR (Lipoproteins, High-Density[Title/Abstract])) OR (alpha-Lipoproteins[Title/Abstract])) OR (alpha Lipoproteins[Title/Abstract])) OR (Heavy Lipoproteins[Title/Abstract])) OR (Lipoproteins, Heavy[Title/Abstract])) OR (High Density Lipoprotein[Title/Abstract])) OR (Density Lipoprotein, High[Title/Abstract])) OR (Lipoprotein, High Density[Title/Abstract])) OR (alpha-Lipoprotein[Title/Abstract])) OR (alpha Lipoprotein[Title/Abstract])) OR (alpha-1 Lipoprotein[Title/Abstract])))) OR (("Lipoproteins, LDL"[Mesh]) OR (((((((((((((((((((LDL Lipoproteins[Title/Abstract]) OR (beta-Lipoprotein[Title/Abstract])) OR (beta Lipoprotein[Title/Abstract])) OR (Low-Density Lipoproteins[Title/Abstract])) OR (Lipoproteins, Low-Density[Title/Abstract])) OR (Low Density Lipoproteins[Title/Abstract])) OR (beta-Lipoproteins[Title/Abstract])) OR (beta Lipoproteins[Title/Abstract])) OR (Low-Density Lipoprotein[Title/Abstract])) OR (Lipoprotein, Low-Density[Title/Abstract])) OR (Low Density Lipoprotein[Title/Abstract])) OR (LDL-2[Title/Abstract])) OR (LDL2[Title/Abstract])) OR (Low-Density Lipoprotein 2[Title/Abstract])) OR (Low Density Lipoprotein 2[Title/Abstract])) OR (LDL-1[Title/Abstract])) OR (LDL1[Title/Abstract])) OR (Low-Density Lipoprotein 1[Title/Abstract])) OR (Low Density Lipoprotein 1[Title/Abstract])))) AND (("Exercise"[Mesh]) OR ((((((((((((((((((((Physical Activity[Title/Abstract]) OR (Activities, Physical[Title/Abstract])) OR (Activity, Physical[Title/Abstract])) OR (Physical Activities[Title/Abstract])) OR (Exercise, Physical[Title/Abstract])) OR (Exercises, Physical[Title/Abstract])) OR (Physical Exercise[Title/Abstract])) OR (Physical Exercises[Title/Abstract])) OR (Exercise, Isometric[Title/Abstract])) OR (Exercises, Isometric[Title/Abstract])) OR (Isometric Exercises[Title/Abstract])) OR (Isometric Exercise[Title/Abstract])) OR (Exercise, Aerobic[Title/Abstract])) OR (Aerobic Exercise[Title/Abstract])) OR (Aerobic Exercises[Title/Abstract])) OR (Exercises, Aerobic[Title/Abstract])) OR (Exercise Training[Title/Abstract])) OR (Exercise Trainings[Title/Abstract])) OR (Training, Exercise[Title/Abstract])) OR (Trainings, Exercise[Title/Abstract])))) AND (randomized controlled trial[Publication Type] OR randomized[Title/Abstract] OR placebo[Title/Abstract])) AND (("Adult"[Mesh]) OR (adults[Title/Abstract]))

#40 (((((((((("Body Mass Index"[Mesh]) OR (((((Index, Body Mass[Title/Abstract]) OR (Quetelet Index[Title/Abstract])) OR (Index, Quetelet[Title/Abstract])) OR (Quetelet's Index[Title/Abstract])) OR (Quetelets Index[Title/Abstract]))) OR (("Blood Pressure"[Mesh]) OR ((((((((Pressure, Blood[Title/Abstract]) OR (Diastolic Pressure[Title/Abstract])) OR (Pressure, Diastolic[Title/Abstract])) OR (Pulse Pressure[Title/Abstract])) OR (Pressure, Pulse[Title/Abstract])) OR (Systolic Pressure[Title/Abstract])) OR (Pressure, Systolic[Title/Abstract])) OR (Pressures, Systolic[Title/Abstract])))) OR (("Blood Glucose"[Mesh]) OR (((Blood Sugar[Title/Abstract]) OR (Sugar, Blood[Title/Abstract])) OR (Glucose, Blood[Title/Abstract])))) OR (("Triglycerides"[Mesh]) OR (((Triacylglycerols[Title/Abstract]) OR (Triacylglycerol[Title/Abstract])) OR (Triglyceride[Title/Abstract])))) OR (("Cholesterol"[Mesh]) OR (Epicholesterol[Title/Abstract]))) OR (("Lipoproteins, HDL"[Mesh]) OR ((((((((((((((((HDL Lipoproteins[Title/Abstract]) OR (High-Density Lipoprotein[Title/Abstract])) OR (Lipoprotein, High-Density[Title/Abstract])) OR (High-Density Lipoproteins[Title/Abstract])) OR (High Density Lipoproteins[Title/Abstract])) OR (Lipoproteins, High-Density[Title/Abstract])) OR (alpha-Lipoproteins[Title/Abstract])) OR (alpha Lipoproteins[Title/Abstract])) OR (Heavy Lipoproteins[Title/Abstract])) OR (Lipoproteins, Heavy[Title/Abstract])) OR (High Density Lipoprotein[Title/Abstract])) OR (Density Lipoprotein, High[Title/Abstract])) OR (Lipoprotein, High Density[Title/Abstract])) OR (alpha-Lipoprotein[Title/Abstract])) OR (alpha Lipoprotein[Title/Abstract])) OR (alpha-1 Lipoprotein[Title/Abstract])))) OR (("Lipoproteins, LDL"[Mesh]) OR (((((((((((((((((((LDL Lipoproteins[Title/Abstract]) OR (beta-Lipoprotein[Title/Abstract])) OR (beta Lipoprotein[Title/Abstract])) OR (Low-Density Lipoproteins[Title/Abstract])) OR (Lipoproteins, Low-Density[Title/Abstract])) OR (Low Density Lipoproteins[Title/Abstract])) OR (beta-Lipoproteins[Title/Abstract])) OR (beta Lipoproteins[Title/Abstract])) OR (Low-Density Lipoprotein[Title/Abstract])) OR (Lipoprotein, Low-Density[Title/Abstract])) OR (Low Density Lipoprotein[Title/Abstract])) OR (LDL-2[Title/Abstract])) OR (LDL2[Title/Abstract])) OR (Low-Density Lipoprotein 2[Title/Abstract])) OR (Low Density Lipoprotein 2[Title/Abstract])) OR (LDL-1[Title/Abstract])) OR (LDL1[Title/Abstract])) OR (Low-Density Lipoprotein 1[Title/Abstract])) OR (Low Density Lipoprotein 1[Title/Abstract])))) AND (("Sedentary Behavior"[Mesh]) OR ((((((((((Behavior, Sedentary[Title/Abstract]) OR (Sedentary Behaviors[Title/Abstract])) OR (Sedentary Lifestyle[Title/Abstract])) OR (Lifestyle, Sedentary[Title/Abstract])) OR (Physical Inactivity[Title/Abstract])) OR (Inactivity, Physical[Title/Abstract])) OR (Lack of Physical Activity[Title/Abstract])) OR (Sedentary Time[Title/Abstract])) OR (Sedentary Times[Title/Abstract])) OR (Time, Sedentary[Title/Abstract])))) (randomized controlled trial[Publication Type] OR randomized[Title/Abstract] OR placebo[Title/Abstract])) AND (("Adult"[Mesh]) OR (adults[Title/Abstract]))

#41 ((((((((((("Body Mass Index"[Mesh]) OR (((((Index, Body Mass[Title/Abstract]) OR (Quetelet Index[Title/Abstract])) OR (Index, Quetelet[Title/Abstract])) OR (Quetelet's Index[Title/Abstract])) OR (Quetelets Index[Title/Abstract]))) OR (("Blood Pressure"[Mesh]) OR ((((((((Pressure, Blood[Title/Abstract]) OR (Diastolic Pressure[Title/Abstract])) OR (Pressure, Diastolic[Title/Abstract])) OR (Pulse Pressure[Title/Abstract])) OR (Pressure, Pulse[Title/Abstract])) OR (Systolic Pressure[Title/Abstract])) OR (Pressure, Systolic[Title/Abstract])) OR (Pressures, Systolic[Title/Abstract])))) OR (("Blood Glucose"[Mesh]) OR (((Blood Sugar[Title/Abstract]) OR (Sugar, Blood[Title/Abstract])) OR (Glucose, Blood[Title/Abstract])))) OR (("Triglycerides"[Mesh]) OR (((Triacylglycerols[Title/Abstract]) OR (Triacylglycerol[Title/Abstract])) OR (Triglyceride[Title/Abstract])))) OR (("Cholesterol"[Mesh]) OR (Epicholesterol[Title/Abstract]))) OR (("Lipoproteins, HDL"[Mesh]) OR ((((((((((((((((HDL Lipoproteins[Title/Abstract]) OR (High-Density Lipoprotein[Title/Abstract])) OR (Lipoprotein, High-Density[Title/Abstract])) OR (High-Density Lipoproteins[Title/Abstract])) OR (High Density Lipoproteins[Title/Abstract])) OR (Lipoproteins, High-Density[Title/Abstract])) OR (alpha-Lipoproteins[Title/Abstract])) OR (alpha Lipoproteins[Title/Abstract])) OR (Heavy Lipoproteins[Title/Abstract])) OR (Lipoproteins, Heavy[Title/Abstract])) OR (High Density Lipoprotein[Title/Abstract])) OR (Density Lipoprotein, High[Title/Abstract])) OR (Lipoprotein, High Density[Title/Abstract])) OR (alpha-Lipoprotein[Title/Abstract])) OR (alpha Lipoprotein[Title/Abstract])) OR (alpha-1 Lipoprotein[Title/Abstract])))) OR (("Lipoproteins, LDL"[Mesh]) OR (((((((((((((((((((LDL Lipoproteins[Title/Abstract]) OR (beta-Lipoprotein[Title/Abstract])) OR (beta Lipoprotein[Title/Abstract])) OR (Low-Density Lipoproteins[Title/Abstract])) OR (Lipoproteins, Low-Density[Title/Abstract])) OR (Low Density Lipoproteins[Title/Abstract])) OR (beta-Lipoproteins[Title/Abstract])) OR (beta Lipoproteins[Title/Abstract])) OR (Low-Density Lipoprotein[Title/Abstract])) OR (Lipoprotein, Low-Density[Title/Abstract])) OR (Low Density Lipoprotein[Title/Abstract])) OR (LDL-2[Title/Abstract])) OR (LDL2[Title/Abstract])) OR (Low-Density Lipoprotein 2[Title/Abstract])) OR (Low Density Lipoprotein 2[Title/Abstract])) OR (LDL-1[Title/Abstract])) OR (LDL1[Title/Abstract])) OR (Low-Density Lipoprotein 1[Title/Abstract])) OR (Low Density Lipoprotein 1[Title/Abstract])))) AND (("Sedentary Behavior"[Mesh]) OR ((((((((((Behavior, Sedentary[Title/Abstract]) OR (Sedentary Behaviors[Title/Abstract])) OR (Sedentary Lifestyle[Title/Abstract])) OR (Lifestyle, Sedentary[Title/Abstract])) OR (Physical Inactivity[Title/Abstract])) OR (Inactivity, Physical[Title/Abstract])) OR (Lack of Physical Activity[Title/Abstract])) OR (Sedentary Time[Title/Abstract])) OR (Sedentary Times[Title/Abstract])) OR (Time, Sedentary[Title/Abstract])))) (randomized controlled trial[Publication Type] OR randomized[Title/Abstract] OR placebo[Title/Abstract])) AND (("Adult"[Mesh]) OR (adults[Title/Abstract]))) OR ((((("Cardiovascular Diseases"[Mesh]) OR (((Cardiovascular Disease[Title/Abstract]) OR (Disease, Cardiovascular[Title/Abstract])) OR (Diseases, Cardiovascular[Title/Abstract]))) AND (("Sedentary Behavior"[Mesh]) OR ((((((((((Behavior, Sedentary[Title/Abstract]) OR (Sedentary Behaviors[Title/Abstract])) OR (Sedentary Lifestyle[Title/Abstract])) OR (Lifestyle, Sedentary[Title/Abstract])) OR (Physical Inactivity[Title/Abstract])) OR (Inactivity, Physical[Title/Abstract])) OR (Lack of Physical Activity[Title/Abstract])) OR (Sedentary Time[Title/Abstract])) OR (Sedentary Times[Title/Abstract])) OR (Time, Sedentary[Title/Abstract])))) AND (cohort studies[mesh:noexp] OR longitudinal studies[mesh:noexp] OR follow-up studies[mesh:noexp] OR prospective studies[mesh:noexp] OR retrospective studies[mesh:noexp] OR cohort[TIAB] OR longitudinal[TIAB] OR prospective[TIAB] OR retrospective[TIAB])) AND (("Adult"[Mesh]) OR (adults[Title/Abstract])))

#42 ((((((((((("Body Mass Index"[Mesh]) OR (((((Index, Body Mass[Title/Abstract]) OR (Quetelet Index[Title/Abstract])) OR (Index, Quetelet[Title/Abstract])) OR (Quetelet's Index[Title/Abstract])) OR (Quetelets Index[Title/Abstract]))) OR (("Blood Pressure"[Mesh]) OR ((((((((Pressure, Blood[Title/Abstract]) OR (Diastolic Pressure[Title/Abstract])) OR (Pressure, Diastolic[Title/Abstract])) OR (Pulse Pressure[Title/Abstract])) OR (Pressure, Pulse[Title/Abstract])) OR (Systolic Pressure[Title/Abstract])) OR (Pressure, Systolic[Title/Abstract])) OR (Pressures, Systolic[Title/Abstract])))) OR (("Blood Glucose"[Mesh]) OR (((Blood Sugar[Title/Abstract]) OR (Sugar, Blood[Title/Abstract])) OR (Glucose, Blood[Title/Abstract])))) OR (("Triglycerides"[Mesh]) OR (((Triacylglycerols[Title/Abstract]) OR (Triacylglycerol[Title/Abstract])) OR (Triglyceride[Title/Abstract])))) OR (("Cholesterol"[Mesh]) OR (Epicholesterol[Title/Abstract]))) OR (("Lipoproteins, HDL"[Mesh]) OR ((((((((((((((((HDL Lipoproteins[Title/Abstract]) OR (High-Density Lipoprotein[Title/Abstract])) OR (Lipoprotein, High-Density[Title/Abstract])) OR (High-Density Lipoproteins[Title/Abstract])) OR (High Density Lipoproteins[Title/Abstract])) OR (Lipoproteins, High-Density[Title/Abstract])) OR (alpha-Lipoproteins[Title/Abstract])) OR (alpha Lipoproteins[Title/Abstract])) OR (Heavy Lipoproteins[Title/Abstract])) OR (Lipoproteins, Heavy[Title/Abstract])) OR (High Density Lipoprotein[Title/Abstract])) OR (Density Lipoprotein, High[Title/Abstract])) OR (Lipoprotein, High Density[Title/Abstract])) OR (alpha-Lipoprotein[Title/Abstract])) OR (alpha Lipoprotein[Title/Abstract])) OR (alpha-1 Lipoprotein[Title/Abstract])))) OR (("Lipoproteins, LDL"[Mesh]) OR (((((((((((((((((((LDL Lipoproteins[Title/Abstract]) OR (beta-Lipoprotein[Title/Abstract])) OR (beta Lipoprotein[Title/Abstract])) OR (Low-Density Lipoproteins[Title/Abstract])) OR (Lipoproteins, Low-Density[Title/Abstract])) OR (Low Density Lipoproteins[Title/Abstract])) OR (beta-Lipoproteins[Title/Abstract])) OR (beta Lipoproteins[Title/Abstract])) OR (Low-Density Lipoprotein[Title/Abstract])) OR (Lipoprotein, Low-Density[Title/Abstract])) OR (Low Density Lipoprotein[Title/Abstract])) OR (LDL-2[Title/Abstract])) OR (LDL2[Title/Abstract])) OR (Low-Density Lipoprotein 2[Title/Abstract])) OR (Low Density Lipoprotein 2[Title/Abstract])) OR (LDL-1[Title/Abstract])) OR (LDL1[Title/Abstract])) OR (Low-Density Lipoprotein 1[Title/Abstract])) OR (Low Density Lipoprotein 1[Title/Abstract])))) AND (("Exercise"[Mesh]) OR ((((((((((((((((((((Physical Activity[Title/Abstract]) OR (Activities, Physical[Title/Abstract])) OR (Activity, Physical[Title/Abstract])) OR (Physical Activities[Title/Abstract])) OR (Exercise, Physical[Title/Abstract])) OR (Exercises, Physical[Title/Abstract])) OR (Physical Exercise[Title/Abstract])) OR (Physical Exercises[Title/Abstract])) OR (Exercise, Isometric[Title/Abstract])) OR (Exercises, Isometric[Title/Abstract])) OR (Isometric Exercises[Title/Abstract])) OR (Isometric Exercise[Title/Abstract])) OR (Exercise, Aerobic[Title/Abstract])) OR (Aerobic Exercise[Title/Abstract])) OR (Aerobic Exercises[Title/Abstract])) OR (Exercises, Aerobic[Title/Abstract])) OR (Exercise Training[Title/Abstract])) OR (Exercise Trainings[Title/Abstract])) OR (Training, Exercise[Title/Abstract])) OR (Trainings, Exercise[Title/Abstract])))) AND (randomized controlled trial[Publication Type] OR randomized[Title/Abstract] OR placebo[Title/Abstract])) AND (("Adult"[Mesh]) OR (adults[Title/Abstract]))) OR ((((("Cardiovascular Diseases"[Mesh]) OR (((Cardiovascular Disease[Title/Abstract]) OR (Disease, Cardiovascular[Title/Abstract])) OR (Diseases, Cardiovascular[Title/Abstract]))) AND (("Exercise"[Mesh]) OR ((((((((((((((((((((Physical Activity[Title/Abstract]) OR (Activities, Physical[Title/Abstract])) OR (Activity, Physical[Title/Abstract])) OR (Physical Activities[Title/Abstract])) OR (Exercise, Physical[Title/Abstract])) OR (Exercises, Physical[Title/Abstract])) OR (Physical Exercise[Title/Abstract])) OR (Physical Exercises[Title/Abstract])) OR (Exercise, Isometric[Title/Abstract])) OR (Exercises, Isometric[Title/Abstract])) OR (Isometric Exercises[Title/Abstract])) OR (Isometric Exercise[Title/Abstract])) OR (Exercise, Aerobic[Title/Abstract])) OR (Aerobic Exercise[Title/Abstract])) OR (Aerobic Exercises[Title/Abstract])) OR (Exercises, Aerobic[Title/Abstract])) OR (Exercise Training[Title/Abstract])) OR (Exercise Trainings[Title/Abstract])) OR (Training, Exercise[Title/Abstract])) OR (Trainings, Exercise[Title/Abstract])))) AND (cohort studies[mesh:noexp] OR longitudinal studies[mesh:noexp] OR follow-up studies[mesh:noexp] OR prospective studies[mesh:noexp] OR retrospective studies[mesh:noexp] OR cohort[TIAB] OR longitudinal[TIAB] OR prospective[TIAB] OR retrospective[TIAB])) AND (("Adult"[Mesh]) OR (adults[Title/Abstract])))

## Embase

#1 'cardiovascular diseases'/exp OR 'cardiovascular diseases'

#2 'cardiovascular disease':ab,ti OR 'disease, cardiovascular':ab,ti OR 'diseases, cardiovascular':ab,ti

#3 ('cardiovascular diseases'/exp OR 'cardiovascular diseases') OR ('cardiovascular disease':ab,ti OR 'disease, cardiovascular':ab,ti OR 'diseases, cardiovascular':ab,ti)

#4 'exercises'

#5 'physical activity':ab,ti OR 'activities, physical':ab,ti OR 'activity, physical':ab,ti OR 'physical activities':ab,ti OR 'exercise, physical':ab,ti OR 'exercises, physical':ab,ti OR 'physical exercise':ab,ti OR 'physical exercises':ab,ti OR 'exercise, isometric':ab,ti OR 'exercises, isometric':ab,ti OR 'isometric exercises':ab,ti OR 'isometric exercise':ab,ti OR 'exercise aerobic':ab,ti OR 'aerobic exercise':ab,ti OR 'aerobic exercises':ab,ti OR 'exercises, aerobic':ab,ti OR 'exercise training':ab,ti OR 'exercise trainings':ab,ti OR 'training, exercise':ab,ti OR 'trainings, exercise':ab,ti

#6 'exercises' OR ('physical activity':ab,ti OR 'activities, physical':ab,ti OR 'activity, physical':ab,ti OR 'physical activities':ab,ti OR 'exercise, physical':ab,ti OR 'exercises, physical':ab,ti OR 'physical exercise':ab,ti OR 'physical exercises':ab,ti OR 'exercise, isometric':ab,ti OR 'exercises, isometric':ab,ti OR 'isometric exercises':ab,ti OR 'isometric exercise':ab,ti OR 'exercise aerobic':ab,ti OR 'aerobic exercise':ab,ti OR 'aerobic exercises':ab,ti OR 'exercises, aerobic':ab,ti OR 'exercise training':ab,ti OR 'exercise trainings':ab,ti OR 'training, exercise':ab,ti OR 'trainings, exercise':ab,ti)

#7 'sedentary behavior'/exp

#8 'behavior, sedentary':ab,ti OR 'sedentary behaviors':ab,ti OR 'sedentary lifestyle':ab,ti OR 'lifestyle, sedentary':ab,ti OR 'physical inactivity':ab,ti OR 'inactivity, physical':ab,ti OR 'lack of physical activity':ab,ti OR 'sedentary time':ab,ti OR 'sedentary times':ab,ti OR 'time, sedentary':ab,ti

#9 'sedentary behavior'/exp OR ('behavior, sedentary':ab,ti OR 'sedentary behaviors':ab,ti OR 'sedentary lifestyle':ab,ti OR 'lifestyle, sedentary':ab,ti OR 'physical inactivity':ab,ti OR 'inactivity, physical':ab,ti OR 'lack of physical activity':ab,ti OR 'sedentary time':ab,ti OR 'sedentary times':ab,ti OR 'time, sedentary':ab,ti)

#10 'cohort studies'/exp OR 'longitudinal studies'/exp OR 'follow-up studies'/exp OR 'prospective studies'/exp OR 'retrospective studies'/exp OR 'cohort' OR 'longitudinal'/exp OR 'prospective' OR 'retrospective'

#11 'adult':ab,ti OR 'adults':ab,ti

#12 (('cardiovascular diseases'/exp OR 'cardiovascular diseases') OR ('cardiovascular disease':ab,ti OR 'disease, cardiovascular':ab,ti OR 'diseases, cardiovascular':ab,ti)) AND ('exercises' OR ('physical activity':ab,ti OR 'activities, physical':ab,ti OR 'activity, physical':ab,ti OR 'physical activities':ab,ti OR 'exercise, physical':ab,ti OR 'exercises, physical':ab,ti OR 'physical exercise':ab,ti OR 'physical exercises':ab,ti OR 'exercise, isometric':ab,ti OR 'exercises, isometric':ab,ti OR 'isometric exercises':ab,ti OR 'isometric exercise':ab,ti OR 'exercise aerobic':ab,ti OR 'aerobic exercise':ab,ti OR 'aerobic exercises':ab,ti OR 'exercises, aerobic':ab,ti OR 'exercise training':ab,ti OR 'exercise trainings':ab,ti OR 'training, exercise':ab,ti OR 'trainings, exercise':ab,ti)) AND ('cohort studies'/exp OR 'longitudinal studies'/exp OR 'follow-up studies'/exp OR 'prospective studies'/exp OR 'retrospective studies'/exp OR 'cohort' OR 'longitudinal'/exp OR 'prospective' OR 'retrospective') AND ('adult':ab,ti OR 'adults':ab,ti)

#13 (('cardiovascular diseases'/exp OR 'cardiovascular diseases') OR ('cardiovascular disease':ab,ti OR 'disease, cardiovascular':ab,ti OR 'diseases, cardiovascular':ab,ti)) AND ('sedentary behavior'/exp OR ('behavior, sedentary':ab,ti OR 'sedentary behaviors':ab,ti OR 'sedentary lifestyle':ab,ti OR 'lifestyle, sedentary':ab,ti OR 'physical inactivity':ab,ti OR 'inactivity, physical':ab,ti OR 'lack of physical activity':ab,ti OR 'sedentary time':ab,ti OR 'sedentary times':ab,ti OR 'time, sedentary':ab,ti)) AND ('cohort studies'/exp OR 'longitudinal studies'/exp OR 'follow-up studies'/exp OR 'prospective studies'/exp OR 'retrospective studies'/exp OR 'cohort' OR 'longitudinal'/exp OR 'prospective' OR 'retrospective') AND ('adult':ab,ti OR 'adults':ab,ti)

#14 'body mass index'/exp

#15 'index, body mass':ab,ti OR 'quetelet index':ab,ti OR 'index, quetelet':ab,ti OR 'quetelets index':ab,ti

#16 'body mass index'/exp OR ('index, body mass':ab,ti OR 'quetelet index':ab,ti OR 'index, quetelet':ab,ti OR 'quetelets index':ab,ti)

#17 'blood pressure'/exp

#18 'pressure, blood':ab,ti OR 'diastolic pressure':ab,ti OR 'pressure, diastolic':ab,ti OR 'pulse pressure':ab,ti OR 'pressure, pulse':ab,ti OR 'systolic pressure':ab,ti OR 'pressure, systolic':ab,ti OR 'pressures, systolic':ab,ti

#19 'blood pressure'/exp OR ('pressure, blood':ab,ti OR 'diastolic pressure':ab,ti OR 'pressure, diastolic':ab,ti OR 'pulse pressure':ab,ti OR 'pressure, pulse':ab,ti OR 'systolic pressure':ab,ti OR 'pressure, systolic':ab,ti OR 'pressures, systolic':ab,ti)

#20 'blood glucose'/exp

#21 'blood sugar':ab,ti OR 'sugar, blood':ab,ti OR 'glucose, blood':ab,ti

#22 'blood glucose'/exp OR ('blood sugar':ab,ti OR 'sugar, blood':ab,ti OR 'glucose, blood':ab,ti)

#23 'triglycerides'/exp

#24 'triacylglycerols':ab,ti OR 'triacylglycerol':ab,ti OR 'triglyceride':ab,ti

#25 'triglycerides'/exp OR ('triacylglycerols':ab,ti OR 'triacylglycerol':ab,ti OR 'triglyceride':ab,ti)

#26 'cholesterol'/exp

#27 'epicholesterol':ab,ti

#28 'cholesterol'/exp OR 'epicholesterol':ab,ti

#29 'lipoproteins, hdl'/exp

#30 'hdl lipoproteins':ab,ti OR 'high-density lipoprotein':ab,ti OR 'lipoprotein, high-density':ab,ti OR 'high-density lipoproteins':ab,ti OR 'high density lipoproteins':ab,ti OR 'lipoproteins, high-density':ab,ti OR 'alpha-lipoproteins':ab,ti OR 'alpha lipoproteins':ab,ti OR 'heavy lipoproteins':ab,ti OR 'lipoproteins, heavy':ab,ti OR 'high density lipoprotein':ab,ti OR 'density lipoprotein, high':ab,ti OR 'lipoprotein, high density':ab,ti OR 'alpha-lipoprotein':ab,ti OR 'alpha lipoprotein':ab,ti OR 'alpha-1 lipoprotein':ab,ti

#31 'lipoproteins, hdl'/exp OR ('hdl lipoproteins':ab,ti OR 'high-density lipoprotein':ab,ti OR 'lipoprotein, high-density':ab,ti OR 'high-density lipoproteins':ab,ti OR 'high density lipoproteins':ab,ti OR 'lipoproteins, high-density':ab,ti OR 'alpha-lipoproteins':ab,ti OR 'alpha lipoproteins':ab,ti OR 'heavy lipoproteins':ab,ti OR 'lipoproteins, heavy':ab,ti OR 'high density lipoprotein':ab,ti OR 'density lipoprotein, high':ab,ti OR 'lipoprotein, high density':ab,ti OR 'alpha-lipoprotein':ab,ti OR 'alpha lipoprotein':ab,ti OR 'alpha-1 lipoprotein':ab,ti)

#32 'lipoproteins, ldl'/exp

#33 'ldl lipoproteins':ab,ti OR 'beta-lipoprotein':ab,ti OR 'beta lipoprotein':ab,ti OR 'low-density lipoproteins':ab,ti OR 'lipoproteins, low-density':ab,ti OR 'low density lipoproteins':ab,ti OR 'beta-lipoproteins':ab,ti OR 'beta lipoproteins':ab,ti OR 'low-density lipoprotein':ab,ti OR 'lipoprotein, low-density':ab,ti OR 'low density lipoprotein':ab,ti OR 'ldl-2':ab,ti OR 'ldl2':ab,ti OR 'low-density lipoprotein 2':ab,ti OR 'low density lipoprotein 2':ab,ti OR 'ldl-1':ab,ti OR 'ldl1':ab,ti OR 'low-density lipoprotein 1':ab,ti OR 'low density lipoprotein 1':ab,ti

#34 'lipoproteins, ldl'/exp OR ('ldl lipoproteins':ab,ti OR 'beta-lipoprotein':ab,ti OR 'beta lipoprotein':ab,ti OR 'low-density lipoproteins':ab,ti OR 'lipoproteins, low-density':ab,ti OR 'low density lipoproteins':ab,ti OR 'beta-lipoproteins':ab,ti OR 'beta lipoproteins':ab,ti OR 'low-density lipoprotein':ab,ti OR 'lipoprotein, low-density':ab,ti OR 'low density lipoprotein':ab,ti OR 'ldl-2':ab,ti OR 'ldl2':ab,ti OR 'low-density lipoprotein 2':ab,ti OR 'low density lipoprotein 2':ab,ti OR 'ldl-1':ab,ti OR 'ldl1':ab,ti OR 'low-density lipoprotein 1':ab,ti OR 'low density lipoprotein 1':ab,ti)

#35 'randomized controlled trial':ab,ti OR 'randomized':ab,ti OR 'placebo':ab,ti

#36 ('body mass index'/exp OR ('index, body mass':ab,ti OR 'quetelet index':ab,ti OR 'index, quetelet':ab,ti OR 'quetelets index':ab,ti)) OR ('blood pressure'/exp OR ('pressure, blood':ab,ti OR 'diastolic pressure':ab,ti OR 'pressure, diastolic':ab,ti OR 'pulse pressure':ab,ti OR 'pressure, pulse':ab,ti OR 'systolic pressure':ab,ti OR 'pressure, systolic':ab,ti OR 'pressures, systolic':ab,ti)) OR ('blood glucose'/exp OR ('blood sugar':ab,ti OR 'sugar, blood':ab,ti OR 'glucose, blood':ab,ti)) OR ('triglycerides'/exp OR ('triacylglycerols':ab,ti OR 'triacylglycerol':ab,ti OR 'triglyceride':ab,ti)) OR ('cholesterol'/exp OR 'epicholesterol':ab,ti) OR ('lipoproteins, hdl'/exp OR ('hdl lipoproteins':ab,ti OR 'high-density lipoprotein':ab,ti OR 'lipoprotein, high-density':ab,ti OR 'high-density lipoproteins':ab,ti OR 'high density lipoproteins':ab,ti OR 'lipoproteins, high-density':ab,ti OR 'alpha-lipoproteins':ab,ti OR 'alpha lipoproteins':ab,ti OR 'heavy lipoproteins':ab,ti OR 'lipoproteins, heavy':ab,ti OR 'high density lipoprotein':ab,ti OR 'density lipoprotein, high':ab,ti OR 'lipoprotein, high density':ab,ti OR 'alpha-lipoprotein':ab,ti OR 'alpha lipoprotein':ab,ti OR 'alpha-1 lipoprotein':ab,ti)) OR ('lipoproteins, ldl'/exp OR ('ldl lipoproteins':ab,ti OR 'beta-lipoprotein':ab,ti OR 'beta lipoprotein':ab,ti OR 'low-density lipoproteins':ab,ti OR 'lipoproteins, low-density':ab,ti OR 'low density lipoproteins':ab,ti OR 'beta-lipoproteins':ab,ti OR 'beta lipoproteins':ab,ti OR 'low-density lipoprotein':ab,ti OR 'lipoprotein, low-density':ab,ti OR 'low density lipoprotein':ab,ti OR 'ldl-2':ab,ti OR 'ldl2':ab,ti OR 'low-density lipoprotein 2':ab,ti OR 'low density lipoprotein 2':ab,ti OR 'ldl-1':ab,ti OR 'ldl1':ab,ti OR 'low-density lipoprotein 1':ab,ti OR 'low density lipoprotein 1':ab,ti))

#37 ('exercises' OR ('physical activity':ab,ti OR 'activities, physical':ab,ti OR 'activity, physical':ab,ti OR 'physical activities':ab,ti OR 'exercise, physical':ab,ti OR 'exercises, physical':ab,ti OR 'physical exercise':ab,ti OR 'physical exercises':ab,ti OR 'exercise, isometric':ab,ti OR 'exercises, isometric':ab,ti OR 'isometric exercises':ab,ti OR 'isometric exercise':ab,ti OR 'exercise aerobic':ab,ti OR 'aerobic exercise':ab,ti OR 'aerobic exercises':ab,ti OR 'exercises, aerobic':ab,ti OR 'exercise training':ab,ti OR 'exercise trainings':ab,ti OR 'training, exercise':ab,ti OR 'trainings, exercise':ab,ti)) AND ('adult':ab,ti OR 'adults':ab,ti) AND ('randomized controlled trial':ab,ti OR 'randomized':ab,ti OR 'placebo':ab,ti) AND (('body mass index'/exp OR ('index, body mass':ab,ti OR 'quetelet index':ab,ti OR 'index, quetelet':ab,ti OR 'quetelets index':ab,ti)) OR ('blood pressure'/exp OR ('pressure, blood':ab,ti OR 'diastolic pressure':ab,ti OR 'pressure, diastolic':ab,ti OR 'pulse pressure':ab,ti OR 'pressure, pulse':ab,ti OR 'systolic pressure':ab,ti OR 'pressure, systolic':ab,ti OR 'pressures, systolic':ab,ti)) OR ('blood glucose'/exp OR ('blood sugar':ab,ti OR 'sugar, blood':ab,ti OR 'glucose, blood':ab,ti)) OR ('triglycerides'/exp OR ('triacylglycerols':ab,ti OR 'triacylglycerol':ab,ti OR 'triglyceride':ab,ti)) OR ('cholesterol'/exp OR 'epicholesterol':ab,ti) OR ('lipoproteins, hdl'/exp OR ('hdl lipoproteins':ab,ti OR 'high-density lipoprotein':ab,ti OR 'lipoprotein, high-density':ab,ti OR 'high-density lipoproteins':ab,ti OR 'high density lipoproteins':ab,ti OR 'lipoproteins, high-density':ab,ti OR 'alpha-lipoproteins':ab,ti OR 'alpha lipoproteins':ab,ti OR 'heavy lipoproteins':ab,ti OR 'lipoproteins, heavy':ab,ti OR 'high density lipoprotein':ab,ti OR 'density lipoprotein, high':ab,ti OR 'lipoprotein, high density':ab,ti OR 'alpha-lipoprotein':ab,ti OR 'alpha lipoprotein':ab,ti OR 'alpha-1 lipoprotein':ab,ti)) OR ('lipoproteins, ldl'/exp OR ('ldl lipoproteins':ab,ti OR 'beta-lipoprotein':ab,ti OR 'beta lipoprotein':ab,ti OR 'low-density lipoproteins':ab,ti OR 'lipoproteins, low-density':ab,ti OR 'low density lipoproteins':ab,ti OR 'beta-lipoproteins':ab,ti OR 'beta lipoproteins':ab,ti OR 'low-density lipoprotein':ab,ti OR 'lipoprotein, low-density':ab,ti OR 'low density lipoprotein':ab,ti OR 'ldl-2':ab,ti OR 'ldl2':ab,ti OR 'low-density lipoprotein 2':ab,ti OR 'low density lipoprotein 2':ab,ti OR 'ldl-1':ab,ti OR 'ldl1':ab,ti OR 'low-density lipoprotein 1':ab,ti OR 'low density lipoprotein 1':ab,ti)))

#38 ('sedentary behavior'/exp OR ('behavior, sedentary':ab,ti OR 'sedentary behaviors':ab,ti OR 'sedentary lifestyle':ab,ti OR 'lifestyle, sedentary':ab,ti OR 'physical inactivity':ab,ti OR 'inactivity, physical':ab,ti OR 'lack of physical activity':ab,ti OR 'sedentary time':ab,ti OR 'sedentary times':ab,ti OR 'time, sedentary':ab,ti)) AND ('adult':ab,ti OR 'adults':ab,ti) AND ('randomized controlled trial':ab,ti OR 'randomized':ab,ti OR 'placebo':ab,ti) AND (('body mass index'/exp OR ('index, body mass':ab,ti OR 'quetelet index':ab,ti OR 'index, quetelet':ab,ti OR 'quetelets index':ab,ti)) OR ('blood pressure'/exp OR ('pressure, blood':ab,ti OR 'diastolic pressure':ab,ti OR 'pressure, diastolic':ab,ti OR 'pulse pressure':ab,ti OR 'pressure, pulse':ab,ti OR 'systolic pressure':ab,ti OR 'pressure, systolic':ab,ti OR 'pressures, systolic':ab,ti)) OR ('blood glucose'/exp OR ('blood sugar':ab,ti OR 'sugar, blood':ab,ti OR 'glucose, blood':ab,ti)) OR ('triglycerides'/exp OR ('triacylglycerols':ab,ti OR 'triacylglycerol':ab,ti OR 'triglyceride':ab,ti)) OR ('cholesterol'/exp OR 'epicholesterol':ab,ti) OR ('lipoproteins, hdl'/exp OR ('hdl lipoproteins':ab,ti OR 'high-density lipoprotein':ab,ti OR 'lipoprotein, high-density':ab,ti OR 'high-density lipoproteins':ab,ti OR 'high density lipoproteins':ab,ti OR 'lipoproteins, high-density':ab,ti OR 'alpha-lipoproteins':ab,ti OR 'alpha lipoproteins':ab,ti OR 'heavy lipoproteins':ab,ti OR 'lipoproteins, heavy':ab,ti OR 'high density lipoprotein':ab,ti OR 'density lipoprotein, high':ab,ti OR 'lipoprotein, high density':ab,ti OR 'alpha-lipoprotein':ab,ti OR 'alpha lipoprotein':ab,ti OR 'alpha-1 lipoprotein':ab,ti)) OR ('lipoproteins, ldl'/exp OR ('ldl lipoproteins':ab,ti OR 'beta-lipoprotein':ab,ti OR 'beta lipoprotein':ab,ti OR 'low-density lipoproteins':ab,ti OR 'lipoproteins, low-density':ab,ti OR 'low density lipoproteins':ab,ti OR 'beta-lipoproteins':ab,ti OR 'beta lipoproteins':ab,ti OR 'low-density lipoprotein':ab,ti OR 'lipoprotein, low-density':ab,ti OR 'low density lipoprotein':ab,ti OR 'ldl-2':ab,ti OR 'ldl2':ab,ti OR 'low-density lipoprotein 2':ab,ti OR 'low density lipoprotein 2':ab,ti OR 'ldl-1':ab,ti OR 'ldl1':ab,ti OR 'low-density lipoprotein 1':ab,ti OR 'low density lipoprotein 1':ab,ti)))

#39 ((('cardiovascular diseases'/exp OR 'cardiovascular diseases') OR ('cardiovascular disease':ab,ti OR 'disease, cardiovascular':ab,ti OR 'diseases, cardiovascular':ab,ti)) AND ('exercises' OR ('physical activity':ab,ti OR 'activities, physical':ab,ti OR 'activity, physical':ab,ti OR 'physical activities':ab,ti OR 'exercise, physical':ab,ti OR 'exercises, physical':ab,ti OR 'physical exercise':ab,ti OR 'physical exercises':ab,ti OR 'exercise, isometric':ab,ti OR 'exercises, isometric':ab,ti OR 'isometric exercises':ab,ti OR 'isometric exercise':ab,ti OR 'exercise aerobic':ab,ti OR 'aerobic exercise':ab,ti OR 'aerobic exercises':ab,ti OR 'exercises, aerobic':ab,ti OR 'exercise training':ab,ti OR 'exercise trainings':ab,ti OR 'training, exercise':ab,ti OR 'trainings, exercise':ab,ti)) AND ('cohort studies'/exp OR 'longitudinal studies'/exp OR 'follow-up studies'/exp OR 'prospective studies'/exp OR 'retrospective studies'/exp OR 'cohort' OR 'longitudinal'/exp OR 'prospective' OR 'retrospective') AND ('adult':ab,ti OR 'adults':ab,ti)) OR (('exercises' OR ('physical activity':ab,ti OR 'activities, physical':ab,ti OR 'activity, physical':ab,ti OR 'physical activities':ab,ti OR 'exercise, physical':ab,ti OR 'exercises, physical':ab,ti OR 'physical exercise':ab,ti OR 'physical exercises':ab,ti OR 'exercise, isometric':ab,ti OR 'exercises, isometric':ab,ti OR 'isometric exercises':ab,ti OR 'isometric exercise':ab,ti OR 'exercise aerobic':ab,ti OR 'aerobic exercise':ab,ti OR 'aerobic exercises':ab,ti OR 'exercises, aerobic':ab,ti OR 'exercise training':ab,ti OR 'exercise trainings':ab,ti OR 'training, exercise':ab,ti OR 'trainings, exercise':ab,ti)) AND ('adult':ab,ti OR 'adults':ab,ti) AND ('randomized controlled trial':ab,ti OR 'randomized':ab,ti OR 'placebo':ab,ti) AND (('body mass index'/exp OR ('index, body mass':ab,ti OR 'quetelet index':ab,ti OR 'index, quetelet':ab,ti OR 'quetelets index':ab,ti)) OR ('blood pressure'/exp OR ('pressure, blood':ab,ti OR 'diastolic pressure':ab,ti OR 'pressure, diastolic':ab,ti OR 'pulse pressure':ab,ti OR 'pressure, pulse':ab,ti OR 'systolic pressure':ab,ti OR 'pressure, systolic':ab,ti OR 'pressures, systolic':ab,ti)) OR ('blood glucose'/exp OR ('blood sugar':ab,ti OR 'sugar, blood':ab,ti OR 'glucose, blood':ab,ti)) OR ('triglycerides'/exp OR ('triacylglycerols':ab,ti OR 'triacylglycerol':ab,ti OR 'triglyceride':ab,ti)) OR ('cholesterol'/exp OR 'epicholesterol':ab,ti) OR ('lipoproteins, hdl'/exp OR ('hdl lipoproteins':ab,ti OR 'high-density lipoprotein':ab,ti OR 'lipoprotein, high-density':ab,ti OR 'high-density lipoproteins':ab,ti OR 'high density lipoproteins':ab,ti OR 'lipoproteins, high-density':ab,ti OR 'alpha-lipoproteins':ab,ti OR 'alpha lipoproteins':ab,ti OR 'heavy lipoproteins':ab,ti OR 'lipoproteins, heavy':ab,ti OR 'high density lipoprotein':ab,ti OR 'density lipoprotein, high':ab,ti OR 'lipoprotein, high density':ab,ti OR 'alpha-lipoprotein':ab,ti OR 'alpha lipoprotein':ab,ti OR 'alpha-1 lipoprotein':ab,ti)) OR ('lipoproteins, ldl'/exp OR ('ldl lipoproteins':ab,ti OR 'beta-lipoprotein':ab,ti OR 'beta lipoprotein':ab,ti OR 'low-density lipoproteins':ab,ti OR 'lipoproteins, low-density':ab,ti OR 'low density lipoproteins':ab,ti OR 'beta-lipoproteins':ab,ti OR 'beta lipoproteins':ab,ti OR 'low-density lipoprotein':ab,ti OR 'lipoprotein, low-density':ab,ti OR 'low density lipoprotein':ab,ti OR 'ldl-2':ab,ti OR 'ldl2':ab,ti OR 'low-density lipoprotein 2':ab,ti OR 'low density lipoprotein 2':ab,ti OR 'ldl-1':ab,ti OR 'ldl1':ab,ti OR 'low-density lipoprotein 1':ab,ti OR 'low density lipoprotein 1':ab,ti))))

#40 ((('cardiovascular diseases'/exp OR 'cardiovascular diseases') OR ('cardiovascular disease':ab,ti OR 'disease, cardiovascular':ab,ti OR 'diseases, cardiovascular':ab,ti)) AND ('sedentary behavior'/exp OR ('behavior, sedentary':ab,ti OR 'sedentary behaviors':ab,ti OR 'sedentary lifestyle':ab,ti OR 'lifestyle, sedentary':ab,ti OR 'physical inactivity':ab,ti OR 'inactivity, physical':ab,ti OR 'lack of physical activity':ab,ti OR 'sedentary time':ab,ti OR 'sedentary times':ab,ti OR 'time, sedentary':ab,ti)) AND ('cohort studies'/exp OR 'longitudinal studies'/exp OR 'follow-up studies'/exp OR 'prospective studies'/exp OR 'retrospective studies'/exp OR 'cohort' OR 'longitudinal'/exp OR 'prospective' OR 'retrospective') AND ('adult':ab,ti OR 'adults':ab,ti)) OR (('sedentary behavior'/exp OR ('behavior, sedentary':ab,ti OR 'sedentary behaviors':ab,ti OR 'sedentary lifestyle':ab,ti OR 'lifestyle, sedentary':ab,ti OR 'physical inactivity':ab,ti OR 'inactivity, physical':ab,ti OR 'lack of physical activity':ab,ti OR 'sedentary time':ab,ti OR 'sedentary times':ab,ti OR 'time, sedentary':ab,ti)) AND ('adult':ab,ti OR 'adults':ab,ti) AND ('randomized controlled trial':ab,ti OR 'randomized':ab,ti OR 'placebo':ab,ti) AND (('body mass index'/exp OR ('index, body mass':ab,ti OR 'quetelet index':ab,ti OR 'index, quetelet':ab,ti OR 'quetelets index':ab,ti)) OR ('blood pressure'/exp OR ('pressure, blood':ab,ti OR 'diastolic pressure':ab,ti OR 'pressure, diastolic':ab,ti OR 'pulse pressure':ab,ti OR 'pressure, pulse':ab,ti OR 'systolic pressure':ab,ti OR 'pressure, systolic':ab,ti OR 'pressures, systolic':ab,ti)) OR ('blood glucose'/exp OR ('blood sugar':ab,ti OR 'sugar, blood':ab,ti OR 'glucose, blood':ab,ti)) OR ('triglycerides'/exp OR ('triacylglycerols':ab,ti OR 'triacylglycerol':ab,ti OR 'triglyceride':ab,ti)) OR ('cholesterol'/exp OR 'epicholesterol':ab,ti) OR ('lipoproteins, hdl'/exp OR ('hdl lipoproteins':ab,ti OR 'high-density lipoprotein':ab,ti OR 'lipoprotein, high-density':ab,ti OR 'high-density lipoproteins':ab,ti OR 'high density lipoproteins':ab,ti OR 'lipoproteins, high-density':ab,ti OR 'alpha-lipoproteins':ab,ti OR 'alpha lipoproteins':ab,ti OR 'heavy lipoproteins':ab,ti OR 'lipoproteins, heavy':ab,ti OR 'high density lipoprotein':ab,ti OR 'density lipoprotein, high':ab,ti OR 'lipoprotein, high density':ab,ti OR 'alpha-lipoprotein':ab,ti OR 'alpha lipoprotein':ab,ti OR 'alpha-1 lipoprotein':ab,ti)) OR ('lipoproteins, ldl'/exp OR ('ldl lipoproteins':ab,ti OR 'beta-lipoprotein':ab,ti OR 'beta lipoprotein':ab,ti OR 'low-density lipoproteins':ab,ti OR 'lipoproteins, low-density':ab,ti OR 'low density lipoproteins':ab,ti OR 'beta-lipoproteins':ab,ti OR 'beta lipoproteins':ab,ti OR 'low-density lipoprotein':ab,ti OR 'lipoprotein, low-density':ab,ti OR 'low density lipoprotein':ab,ti OR 'ldl-2':ab,ti OR 'ldl2':ab,ti OR 'low-density lipoprotein 2':ab,ti OR 'low density lipoprotein 2':ab,ti OR 'ldl-1':ab,ti OR 'ldl1':ab,ti OR 'low-density lipoprotein 1':ab,ti OR 'low density lipoprotein 1':ab,ti))))

## Cochrane

#1 (Body Mass Index):ti,ab,kw OR (Index, Body Mass):ti,ab,kw OR (Quetelet Index):ti,ab,kw OR (Index, Quetelet):ti,ab,kw OR (Quetelet's Index):ti,ab,kw OR (Quetelets Index):ti,ab,kw OR (Blood Pressure):ti,ab,kw OR (Pressure, Blood):ti,ab,kw OR (Diastolic Pressure):ti,ab,kw OR (Pressure, Diastolic):ti,ab,kw OR (Pulse Pressure):ti,ab,kw OR (Pressure, Pulse):ti,ab,kw OR (Systolic Pressure):ti,ab,kw OR (Pressure, Systolic):ti,ab,kw OR (Pressures, Systolic):ti,ab,kw OR (Blood Glucose):ti,ab,kw OR (Blood Sugar):ti,ab,kw OR (Sugar, Blood):ti,ab,kw OR (Glucose, Blood):ti,ab,kw OR (Triglycerides):ti,ab,kw OR (Triacylglycerols):ti,ab,kw OR (Triacylglycerol):ti,ab,kw OR (Triglyceride):ti,ab,kw OR (Cholesterol):ti,ab,kw OR (Epicholesterol):ti,ab,kw OR (Lipoproteins, HDL):ti,ab,kw OR (HDL Lipoproteins):ti,ab,kw OR (High-Density Lipoprotein):ti,ab,kw OR (Lipoprotein, High-Density):ti,ab,kw OR (High-Density Lipoproteins):ti,ab,kw OR (High Density Lipoproteins):ti,ab,kw OR (Lipoproteins, High-Density):ti,ab,kw OR (alpha-Lipoproteins):ti,ab,kw OR (alpha Lipoproteins):ti,ab,kw OR (Heavy Lipoproteins):ti,ab,kw OR (Lipoproteins, Heavy):ti,ab,kw OR (High Density Lipoprotein):ti,ab,kw OR (Density Lipoprotein, High):ti,ab,kw OR (Lipoprotein, High Density):ti,ab,kw OR (alpha-Lipoprotein):ti,ab,kw OR (alpha Lipoprotein):ti,ab,kw OR (alpha-1 Lipoprotein):ti,ab,kw OR (Lipoproteins, LDL):ti,ab,kw OR (LDL Lipoproteins):ti,ab,kw OR (beta-Lipoprotein):ti,ab,kw OR (beta Lipoprotein):ti,ab,kw OR (Low-Density Lipoproteins):ti,ab,kw OR (Lipoproteins, Low-Density):ti,ab,kw OR (Low Density Lipoproteins):ti,ab,kw OR (beta-Lipoproteins):ti,ab,kw OR (beta Lipoproteins):ti,ab,kw OR (Low-Density Lipoprotein):ti,ab,kw OR (Lipoprotein, Low-Density):ti,ab,kw OR (Low Density Lipoprotein):ti,ab,kw OR (LDL-2):ti,ab,kw OR (LDL2):ti,ab,kw OR (Low-Density Lipoprotein 2):ti,ab,kw OR (Low Density Lipoprotein 2):ti,ab,kw OR (LDL-1):ti,ab,kw OR (LDL1):ti,ab,kw OR (Low-Density Lipoprotein 1):ti,ab,kw OR (Low Density Lipoprotein 1):ti,ab,kw

#2 (Exercises):ti,ab,kw OR (Physical Activity):ti,ab,kw OR (Activities, Physical):ti,ab,kw OR (Activity, Physical):ti,ab,kw OR (Physical Activities):ti,ab,kw OR (Exercise, Physical):ti,ab,kw OR (Exercises, Physical):ti,ab,kw OR (Physical Exercise):ti,ab,kw OR (Physical Exercises):ti,ab,kw OR (Exercise, Isometric):ti,ab,kw OR (Exercises, Isometric):ti,ab,kw OR (Isometric Exercises):ti,ab,kw OR (Isometric Exercise):ti,ab,kw OR (Exercise, Aerobic):ti,ab,kw OR (Aerobic Exercise):ti,ab,kw OR (Aerobic Exercises):ti,ab,kw OR (Exercises, Aerobic):ti,ab,kw OR (Exercise Training):ti,ab,kw OR (Exercise Trainings):ti,ab,kw OR (Training, Exercise):ti,ab,kw OR (Trainings, Exercise):ti,ab,kw

#3 (Sedentary Behavior):ti,ab,kw OR (Behavior, Sedentary):ti,ab,kw OR (Sedentary Behaviors):ti,ab,kw OR (Sedentary Lifestyle):ti,ab,kw OR (Lifestyle, Sedentary):ti,ab,kw OR (Physical Inactivity):ti,ab,kw OR (Inactivity, Physical):ti,ab,kw OR (Lack of Physical Activity):ti,ab,kw OR (Sedentary Time):ti,ab,kw OR (Sedentary Times):ti,ab,kw OR (Time, Sedentary):ti,ab,kw

#4 (adult):ti,ab,kw OR (adults):ti,ab,kw

#5 #1AND#2AND#4

#6 #1AND#3AND#4

## Web of Science

#1 TS=(Cardiovascular Diseases, OR Cardiovascular Disease, OR Disease, Cardiovascular, OR Diseases, Cardiovascular)

#2 TS=(Exercises, OR Physical Activity, OR Activities, Physical, OR Activity, Physical, OR Physical Activities, OR Exercise, Physical, OR Exercises, Physical, OR Physical Exercise, OR Physical Exercises, OR Exercise, Isometric, OR Exercises, Isometric, OR Isometric Exercises, OR Isometric Exercise, OR Exercise, Aerobic, OR Aerobic Exercise, OR Aerobic Exercises, OR Exercises, Aerobic, OR Exercise Training, OR Exercise Trainings, OR Training, Exercise, OR Trainings, Exercise)

#3 TS=(Sedentary Behavior, OR Behavior, Sedentary, OR Sedentary Behaviors, OR Sedentary Lifestyle, OR Lifestyle, Sedentary, OR Physical Inactivity, OR Inactivity, Physical, OR Lack of Physical Activity, OR Sedentary Time, OR Sedentary Times, OR Time, Sedentary)

#4 TS=(cohort studies, OR longitudinal studies, OR follow-up studies, OR prospective studies, OR retrospective studies, OR cohort, OR longitudinal, OR prospective, OR retrospective)

#5 TS=(adult, OR adults)

#6 #1AND#2AND#4AND#5

#7 #1AND#3AND#4AND#5

#8 TS=(Body Mass Index, OR Index, Body Mass, OR Quetelet Index, OR Index, Quetelet, OR Quetelet's Index, OR Quetelets Index, OR Blood Pressure, OR Pressure, Blood, OR Diastolic Pressure, OR Pressure, Diastolic, OR Pulse Pressure, OR Pressure, Pulse, OR Systolic Pressure, OR Pressure, Systolic, OR Pressures, Systolic, OR Blood Glucose, OR Blood Sugar, OR Sugar, Blood, OR Glucose, Blood, OR Triglycerides, OR Triacylglycerols, OR Triacylglycerol, OR Triglyceride, OR Cholesterol, OR Epicholesterol, OR Lipoproteins, HDL, OR HDL Lipoproteins, OR High-Density Lipoprotein, OR Lipoprotein, High-Density, OR High-Density Lipoproteins, OR High Density Lipoproteins, OR Lipoproteins, High-Density, OR alpha-Lipoproteins, OR alpha Lipoproteins, OR Heavy Lipoproteins, OR Lipoproteins, Heavy, OR High Density Lipoprotein, OR Density Lipoprotein, High, OR Lipoprotein, High Density, OR alpha-Lipoprotein, OR alpha Lipoprotein, OR alpha-1 Lipoprotein, OR Lipoproteins, LDL, OR LDL Lipoproteins, OR beta-Lipoprotein, OR beta Lipoprotein, OR Low-Density Lipoproteins, OR Lipoproteins, Low-Density, OR Low Density Lipoproteins, OR beta-Lipoproteins, OR beta Lipoproteins, OR Low-Density Lipoprotein, OR Lipoprotein, Low-Density, OR Low Density Lipoprotein, OR LDL-2, OR LDL2, OR Low-Density Lipoprotein 2, OR Low Density Lipoprotein 2, OR LDL-1, OR LDL1, OR Low-Density Lipoprotein 1, OR Low Density Lipoprotein 1)

#9 TS=(randomized controlled trial OR randomized OR placebo)

#10 #2AND#5AND#8AND#9

#11 #3AND#5AND#8AND#9

#12 #6OR#10

#13 #7OR#11

# Literature quality assessment

## Randomized controlled trials


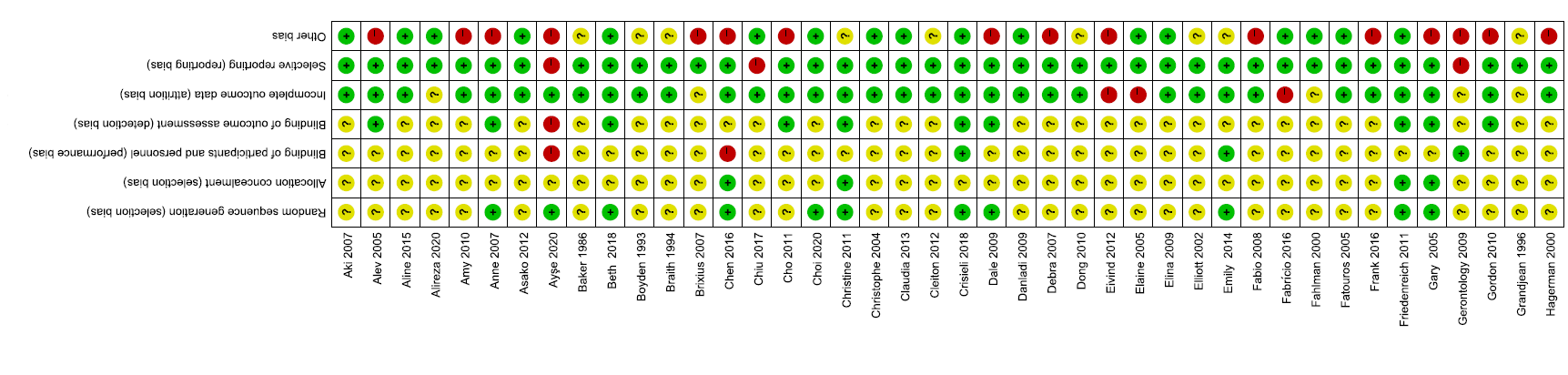

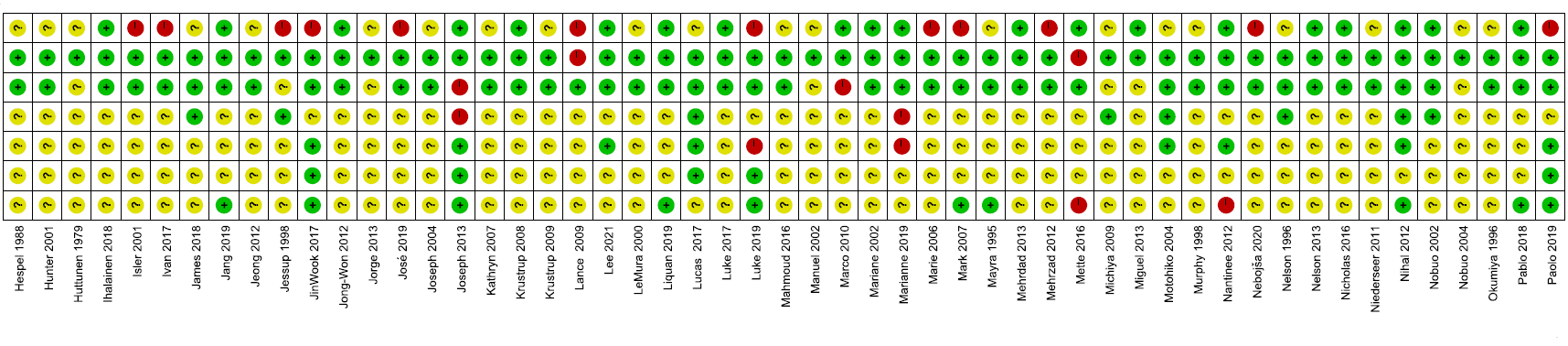

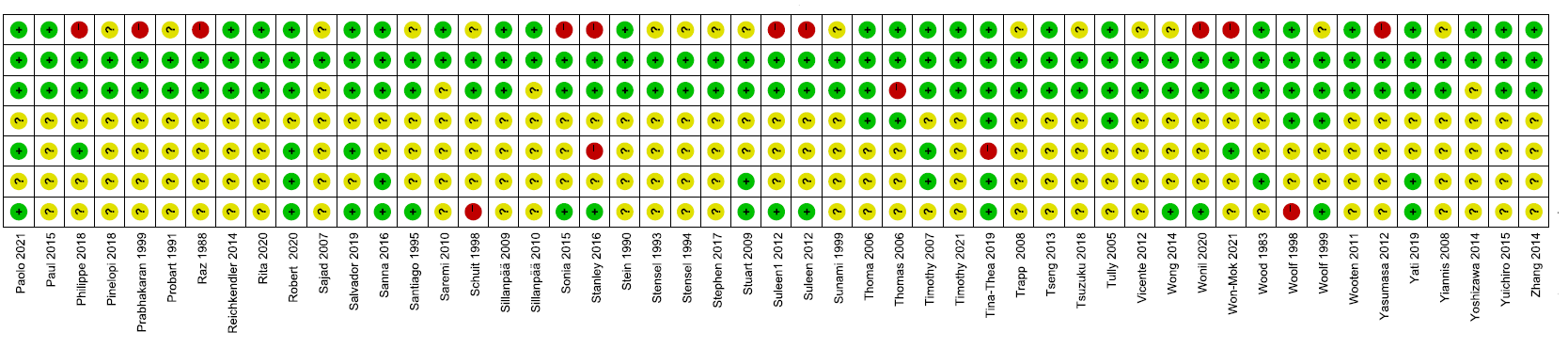


Figure S1. Risk of bias for individual quality.


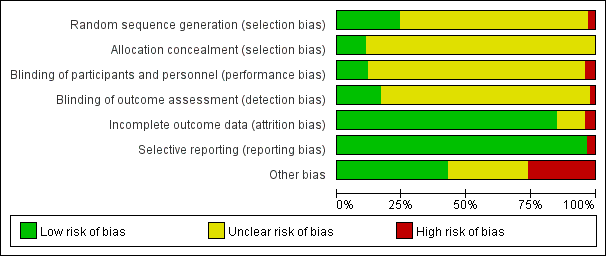


Figure S2. Risk of bias for summary quality.

## Longitudinal studies


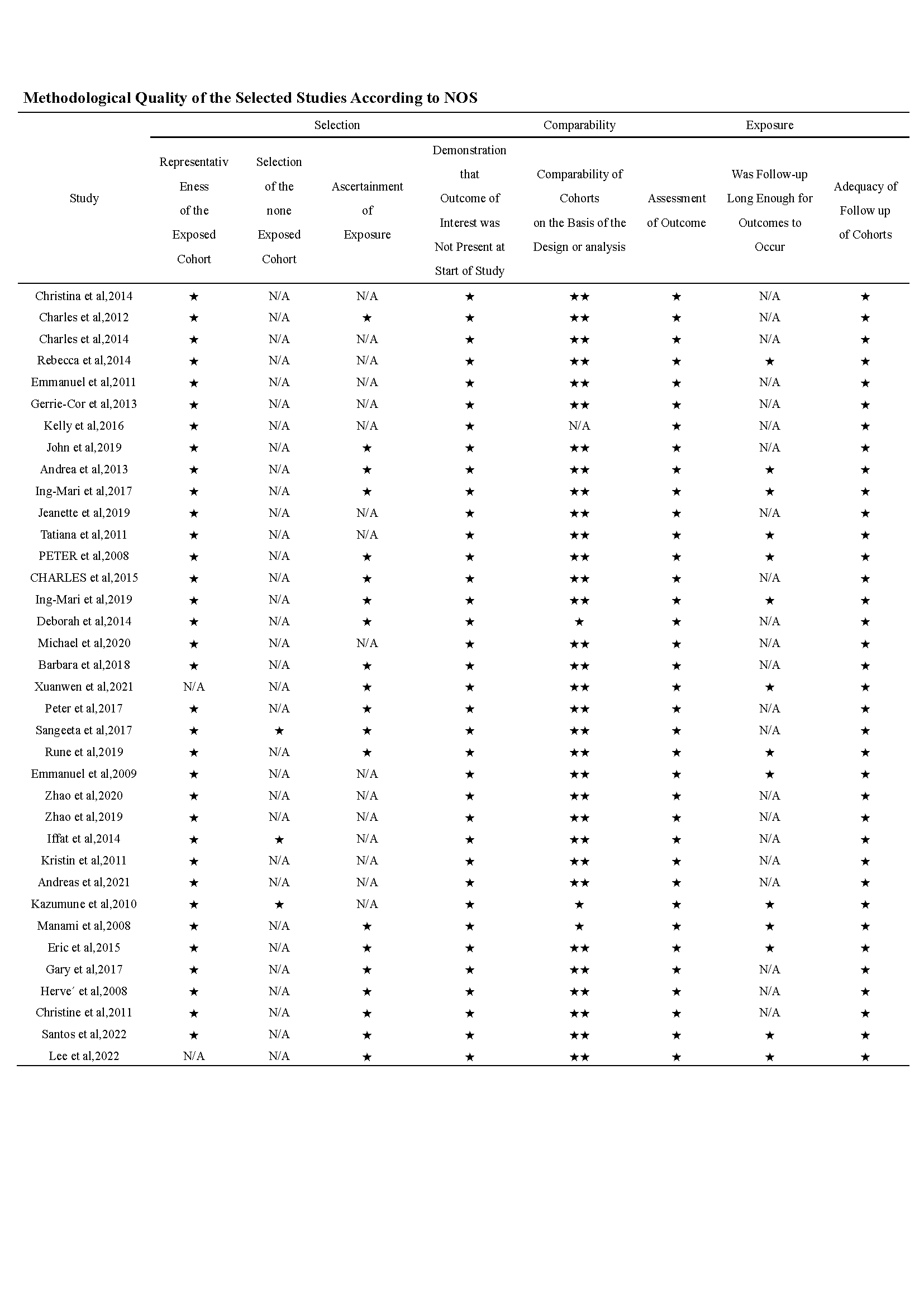
Figure S3. Newcastle-Ottawa Scale for Quality of the included studies. Newcastle-Ottawa Scale for Quality of the included studies. This table identifies “high” quality choices with a “star.” A study can be awarded a maximum of 1 star for each numbered item within the Selection and Exposure categories. A maximum of 2 stars can be given for Comparability. ^★^, yes; N/A, not applicable.

# Forest plot

## Randomized controlled trials


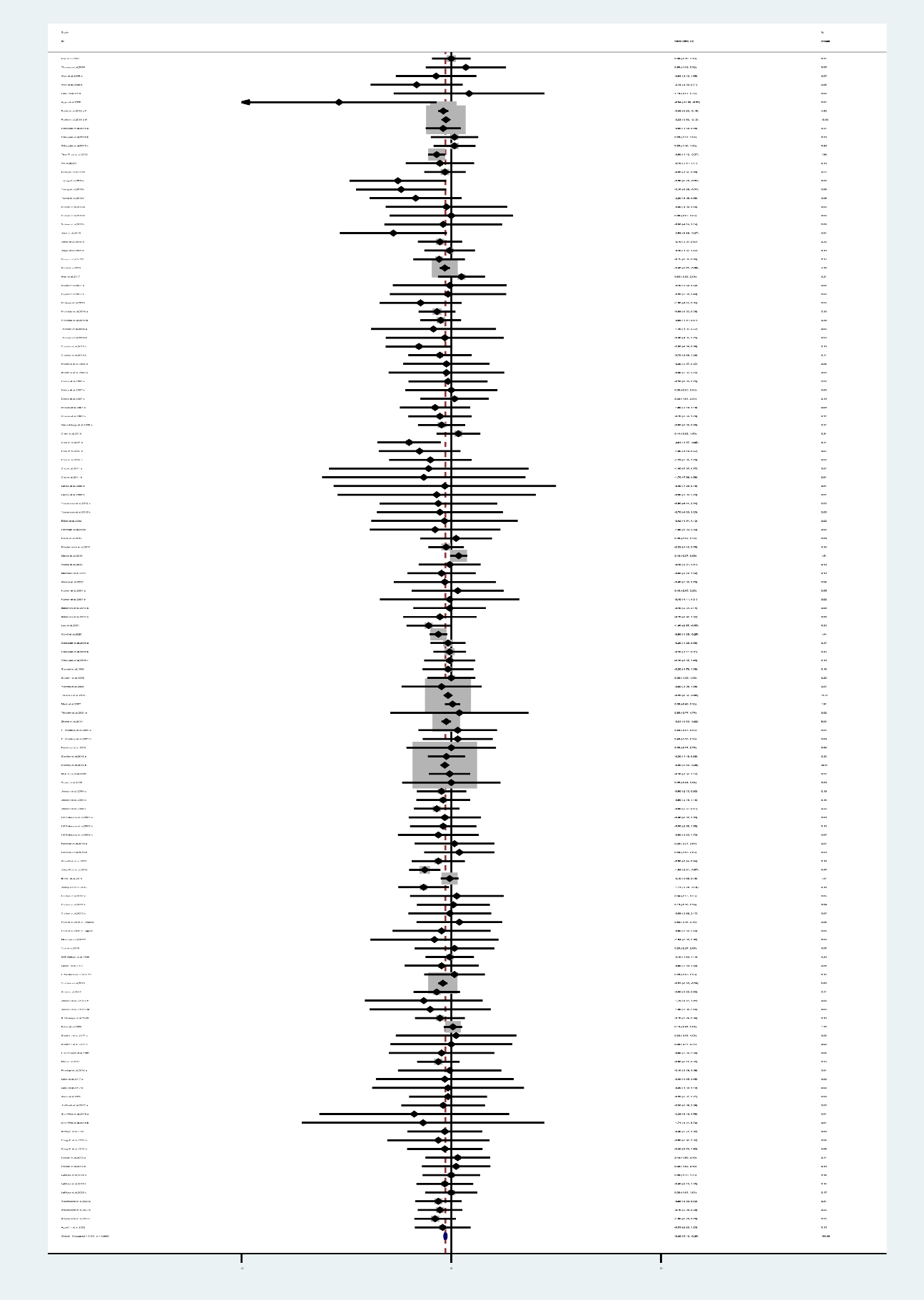


Figure S4. Body mass index forest plot.


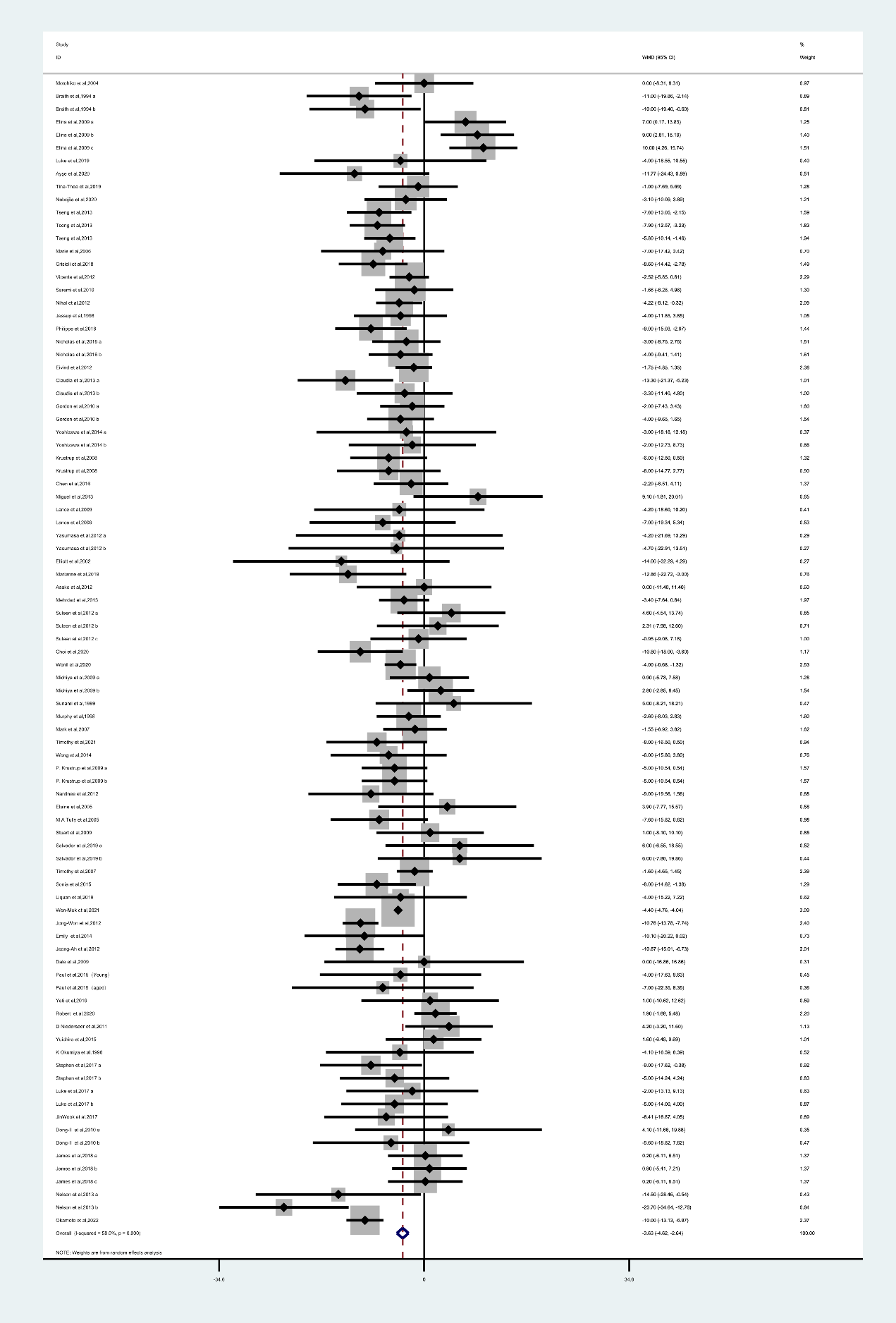


Figure S5. Systolic blood pressure plot.


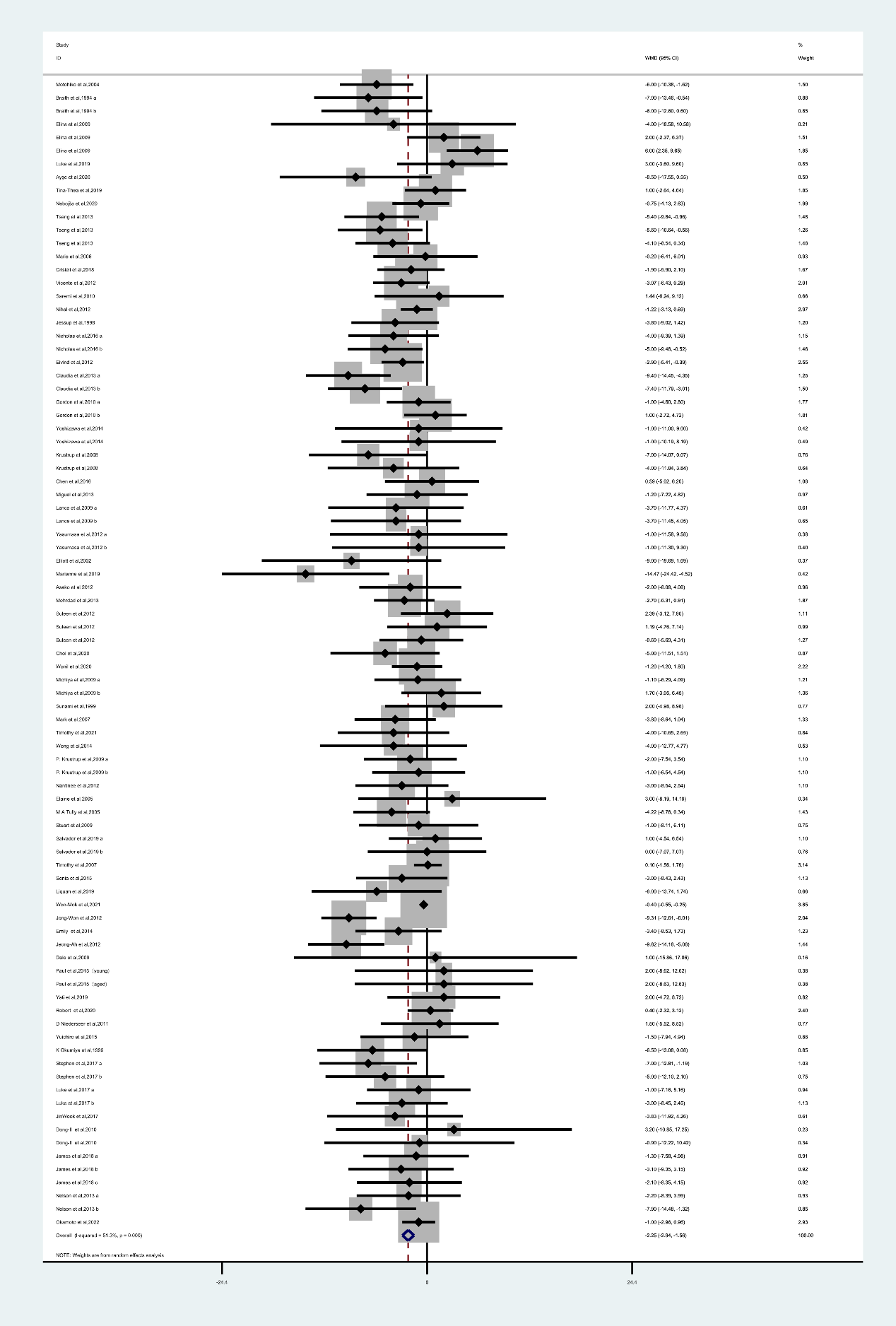


Figure S6. Diastolic blood pressure forest plot.


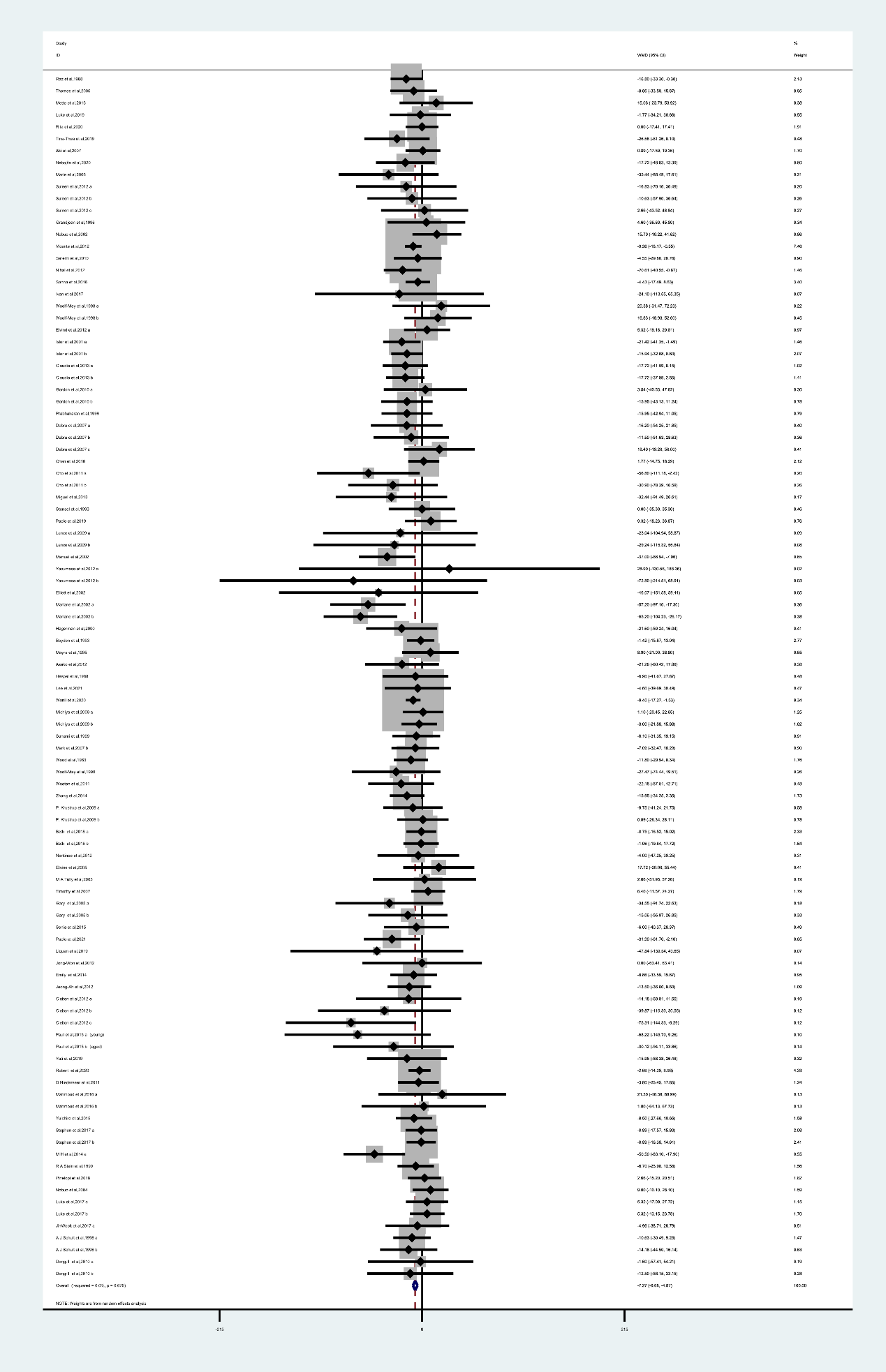


Figure S7. Triglycerides forest plot.
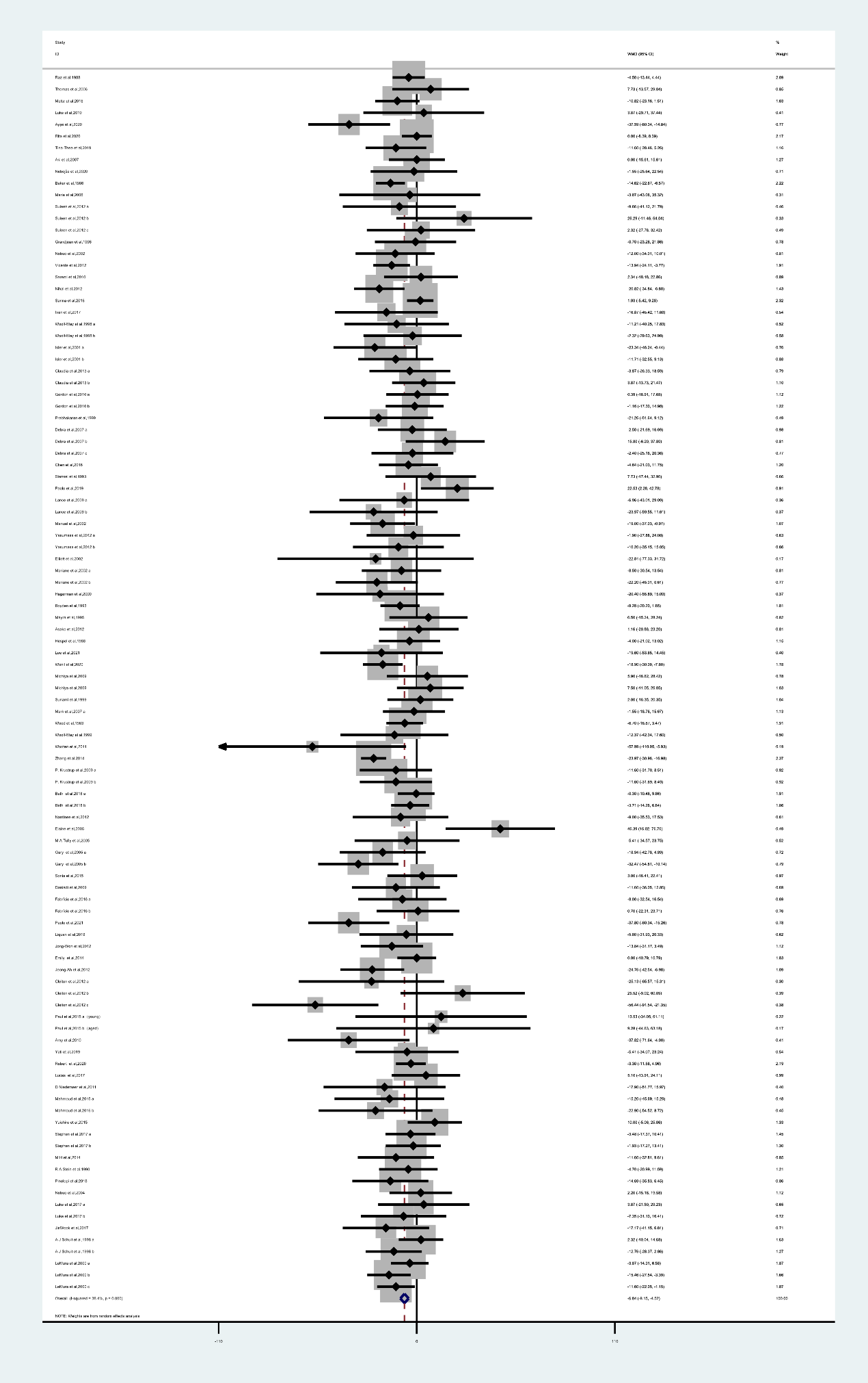


Figure S8. Total cholesterol forest plot.
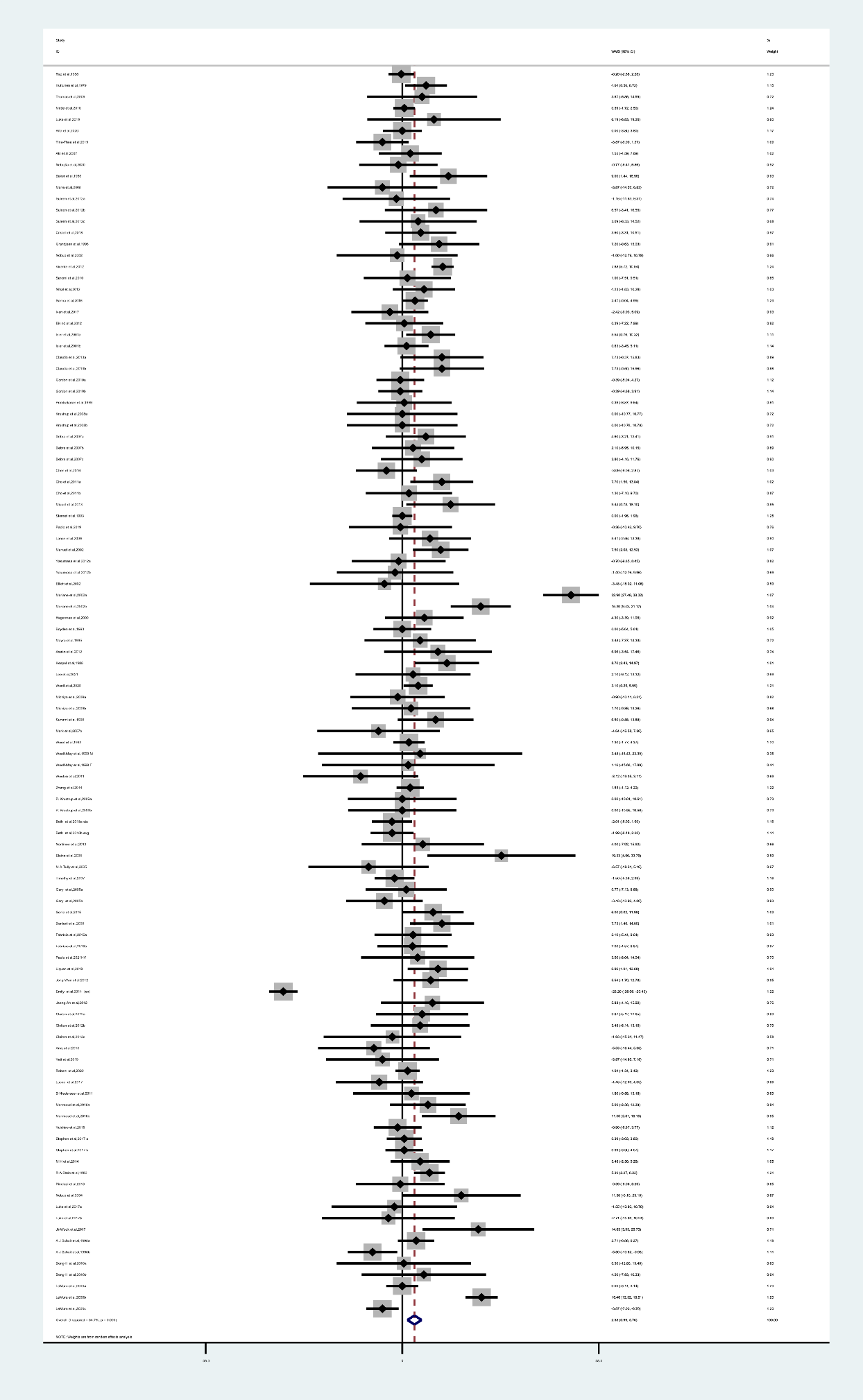


Figure S9. High density lipoprotein forest plot.
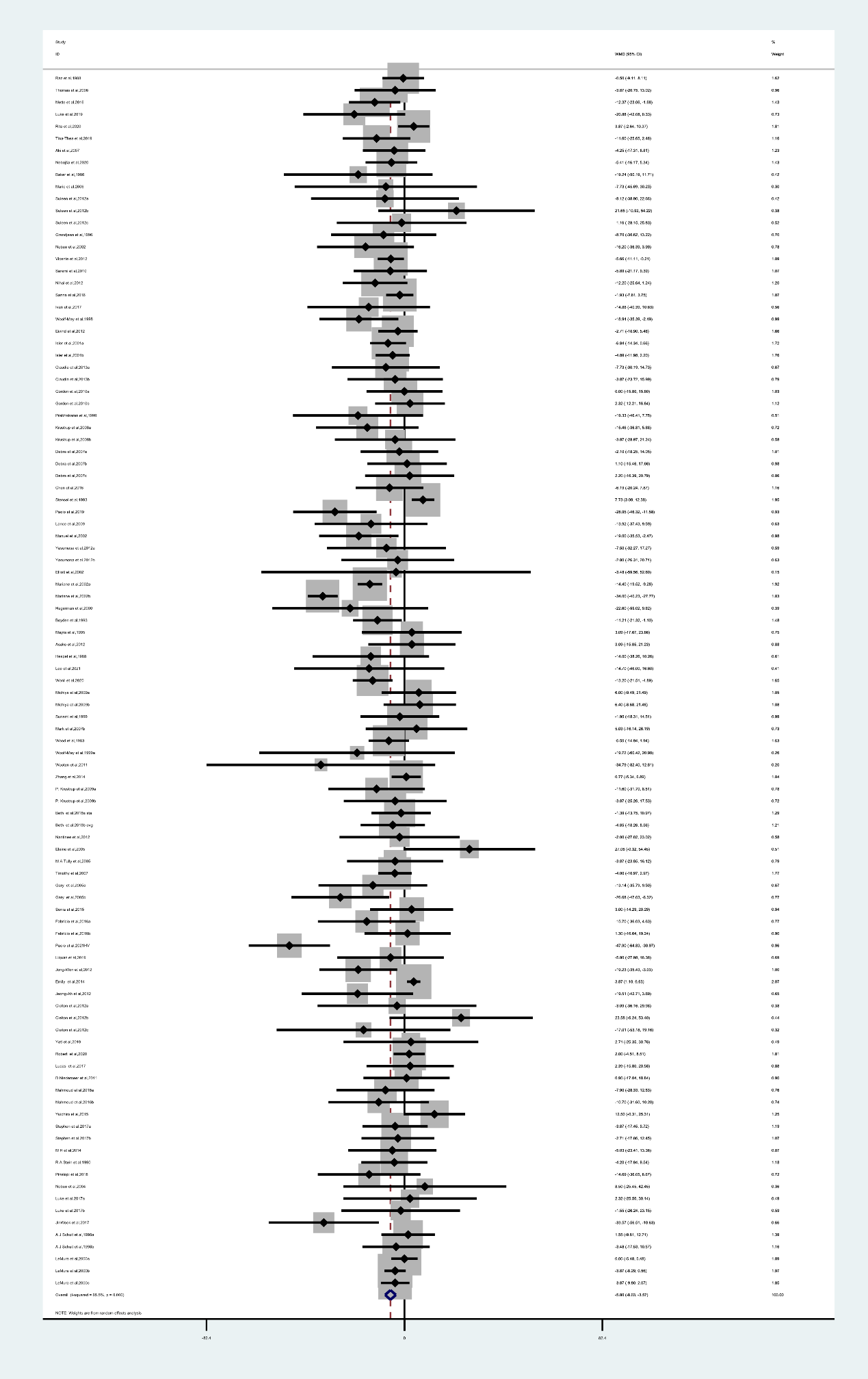


Figure S10. Low density lipoprotein forest plot.
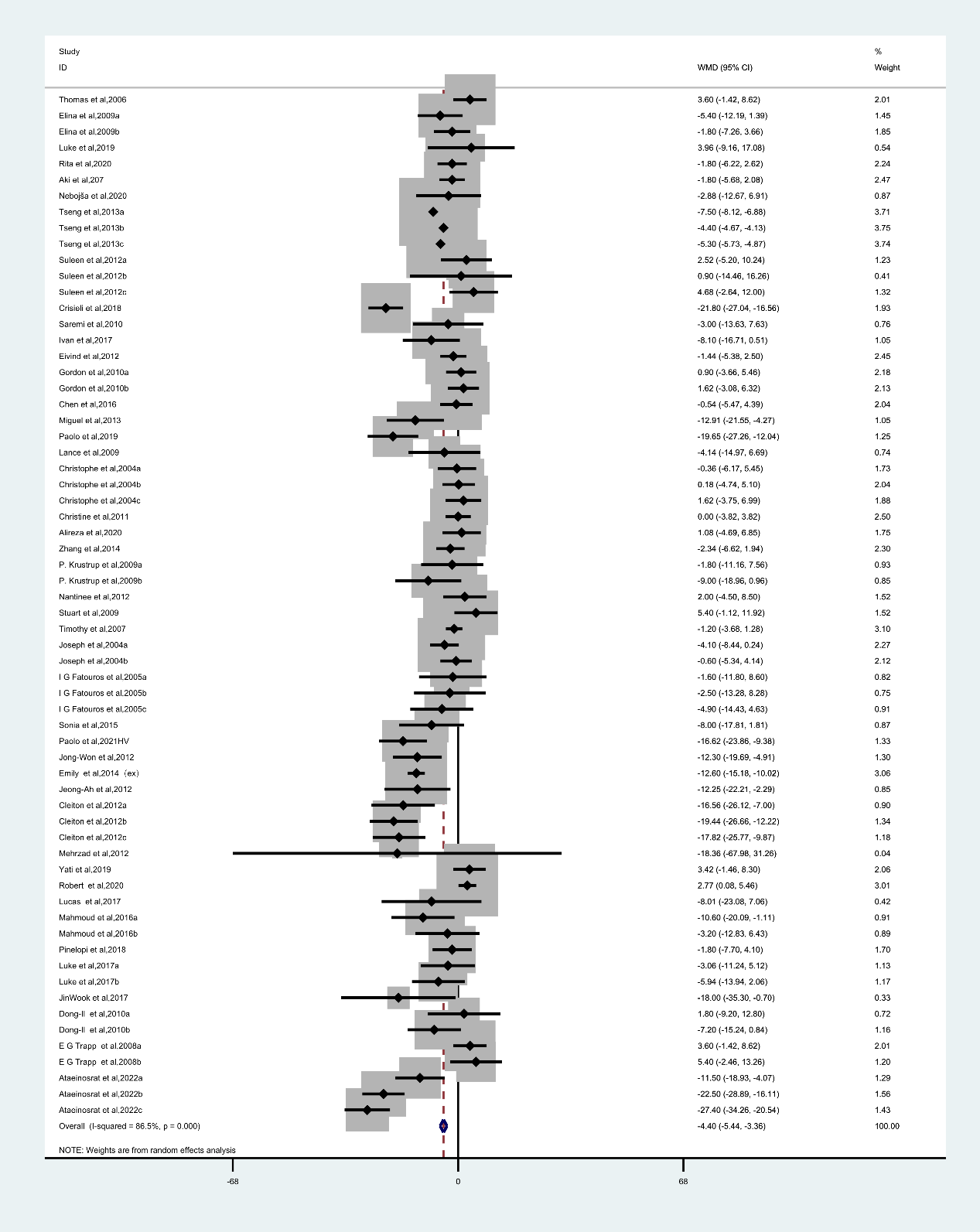


Figure S11. Blood glucose forest plot.

## Longitudinal studies


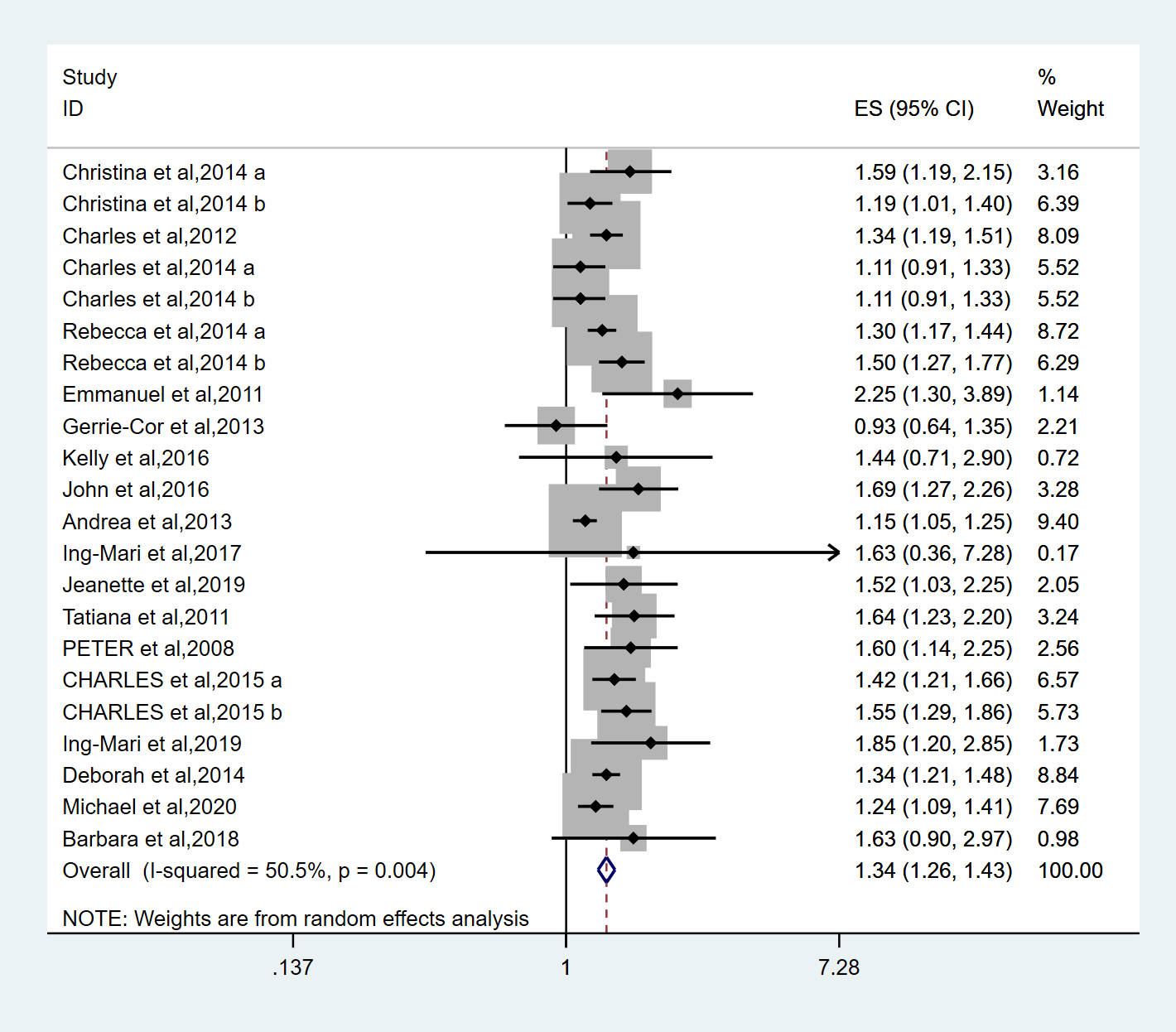


Figure S12. Sedentary behavior forest plot.
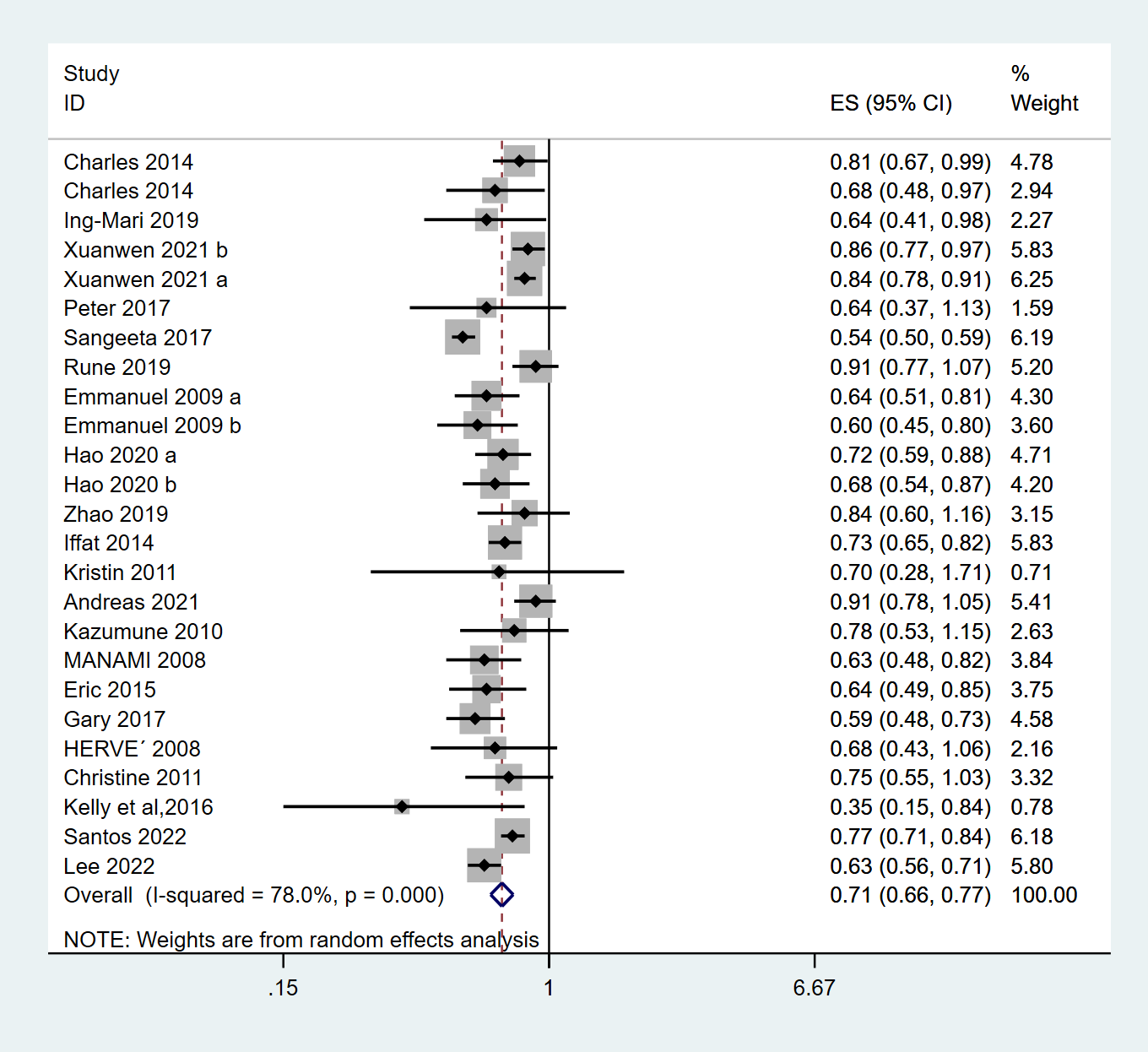


Figure S13. Physical activity forest plot.

# Funnel plot

## Randomized controlled trials


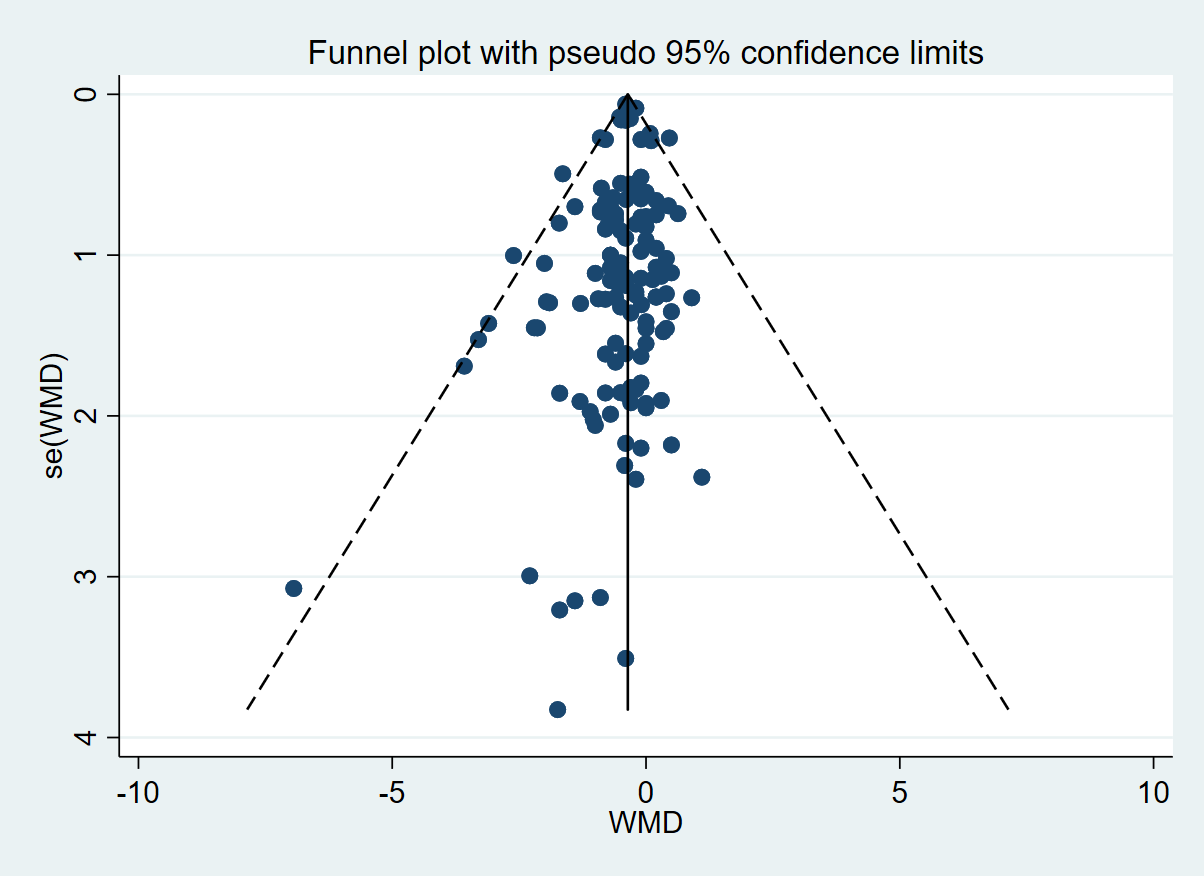


Figure S14. Body mass index funnel plot.
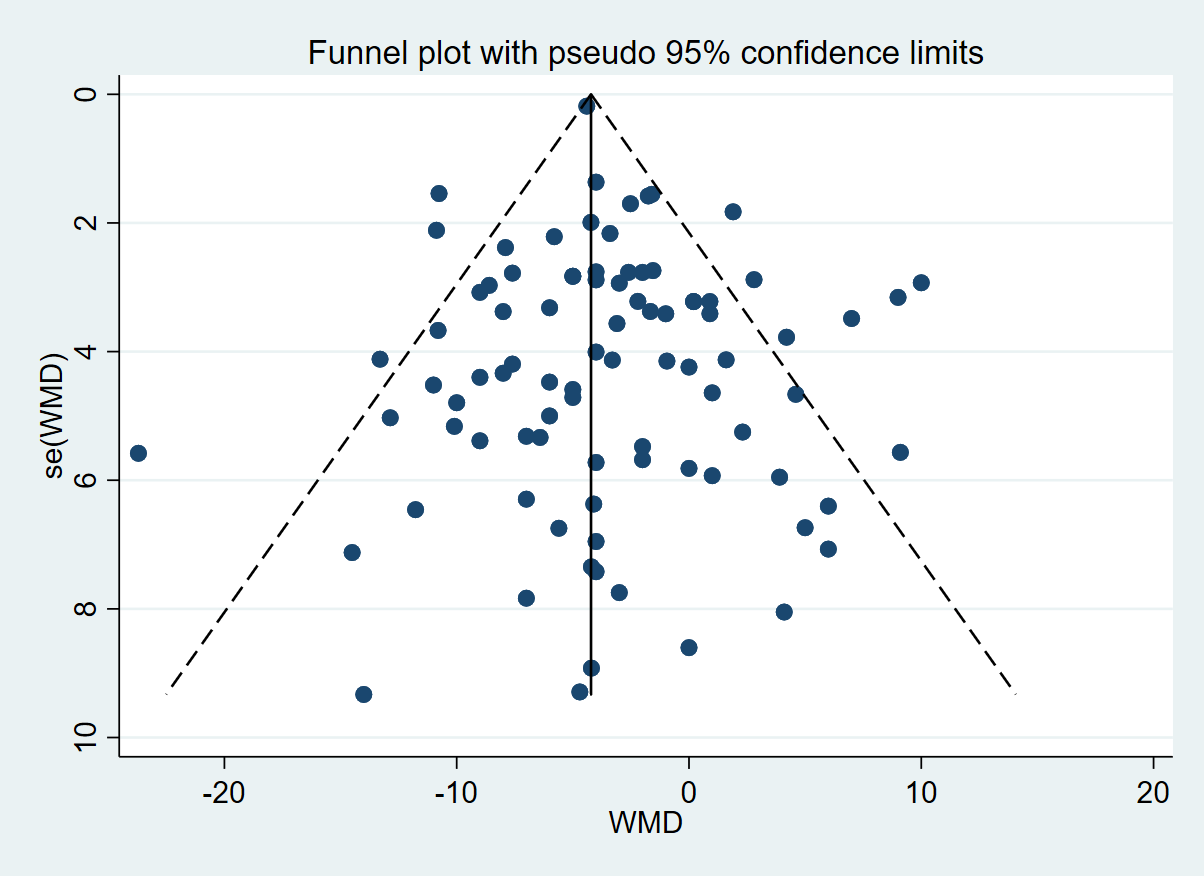


Figure S15. Systolic blood pressure funnel plot.


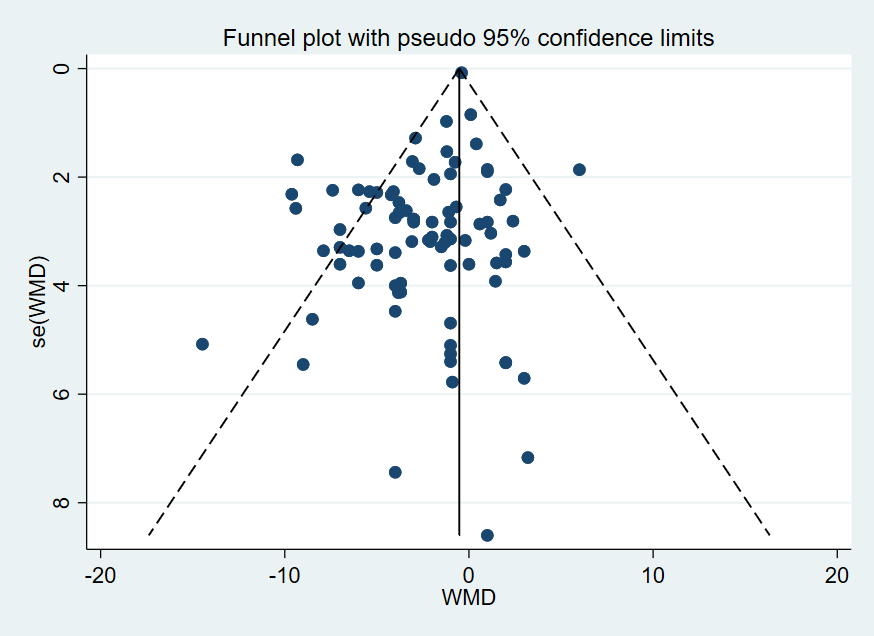


Figure S16. Diastolic blood pressure funnel plot.


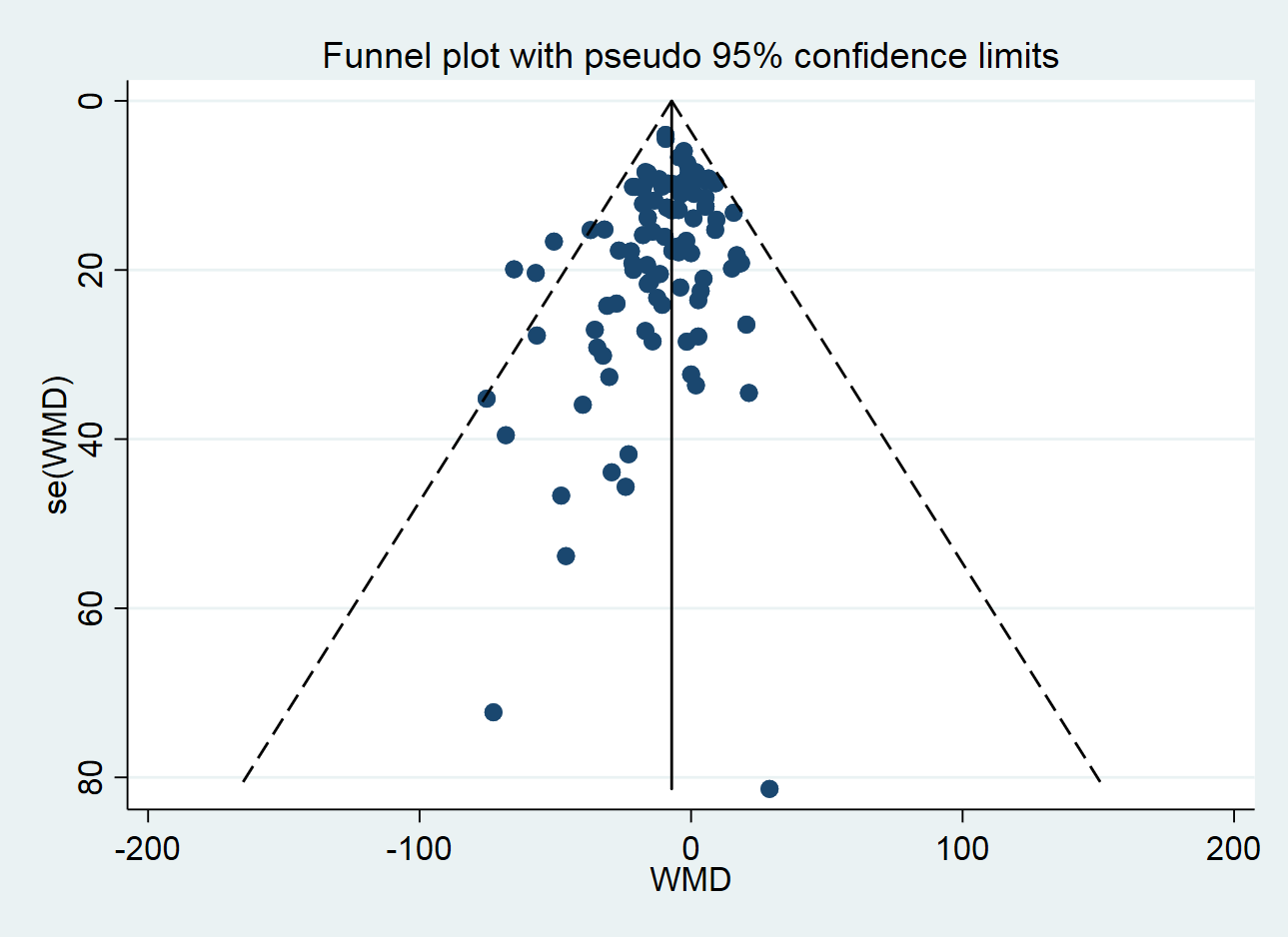


Figure S17. Triglycerides funnel plot.


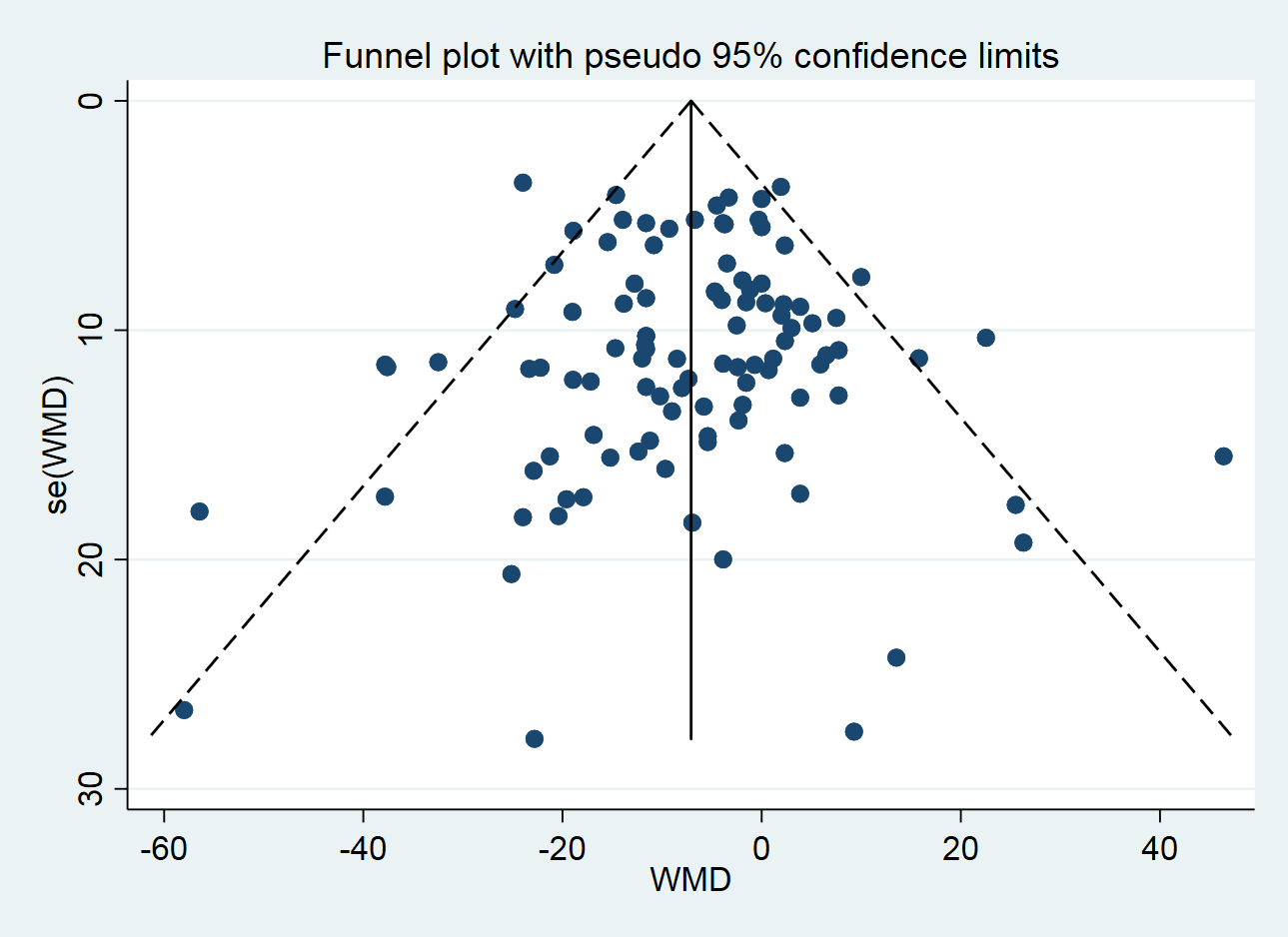


Figure S18. Total cholesterol funnel plot.


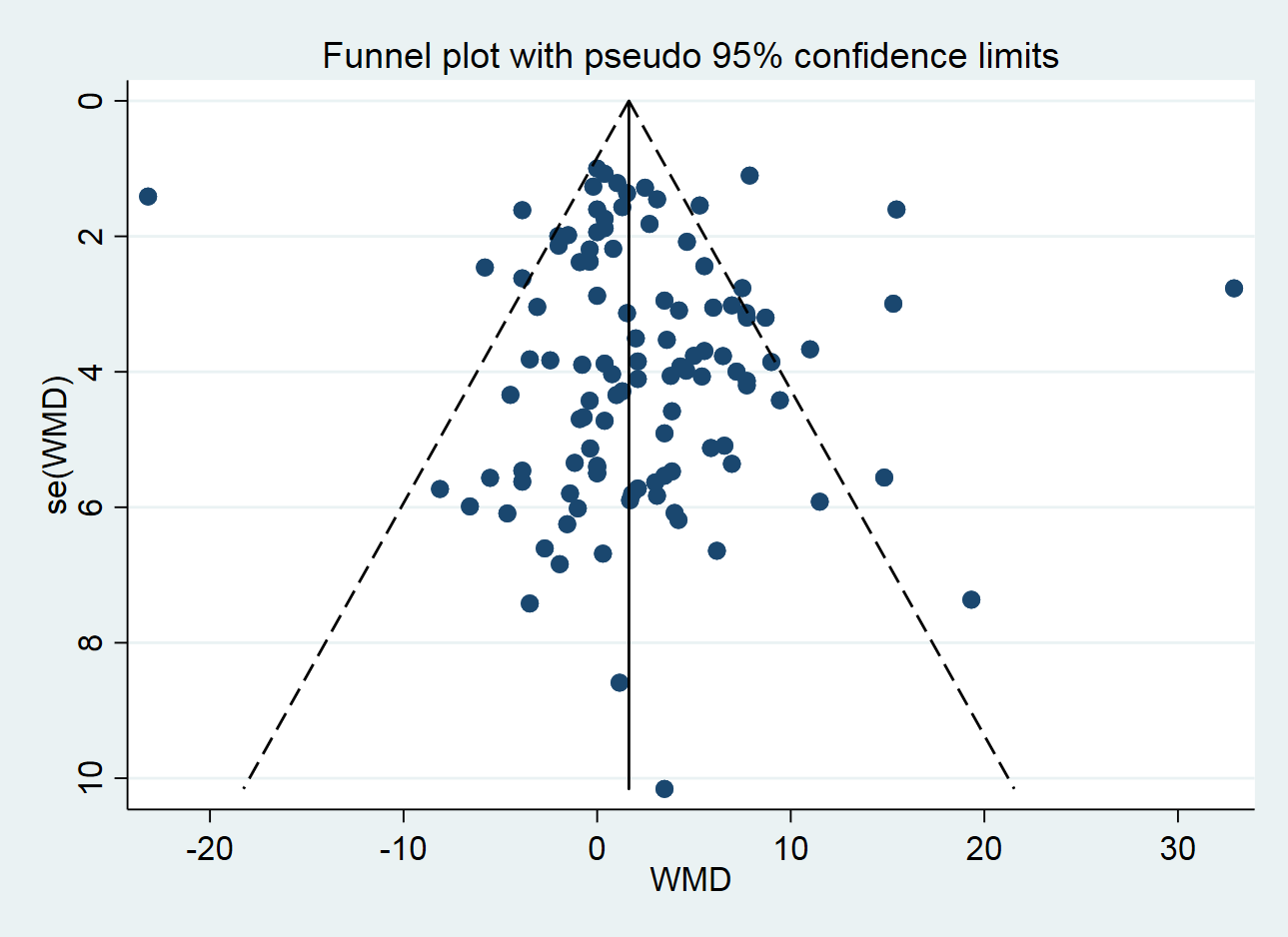


Figure S19. High density lipoprotein funnel plot.


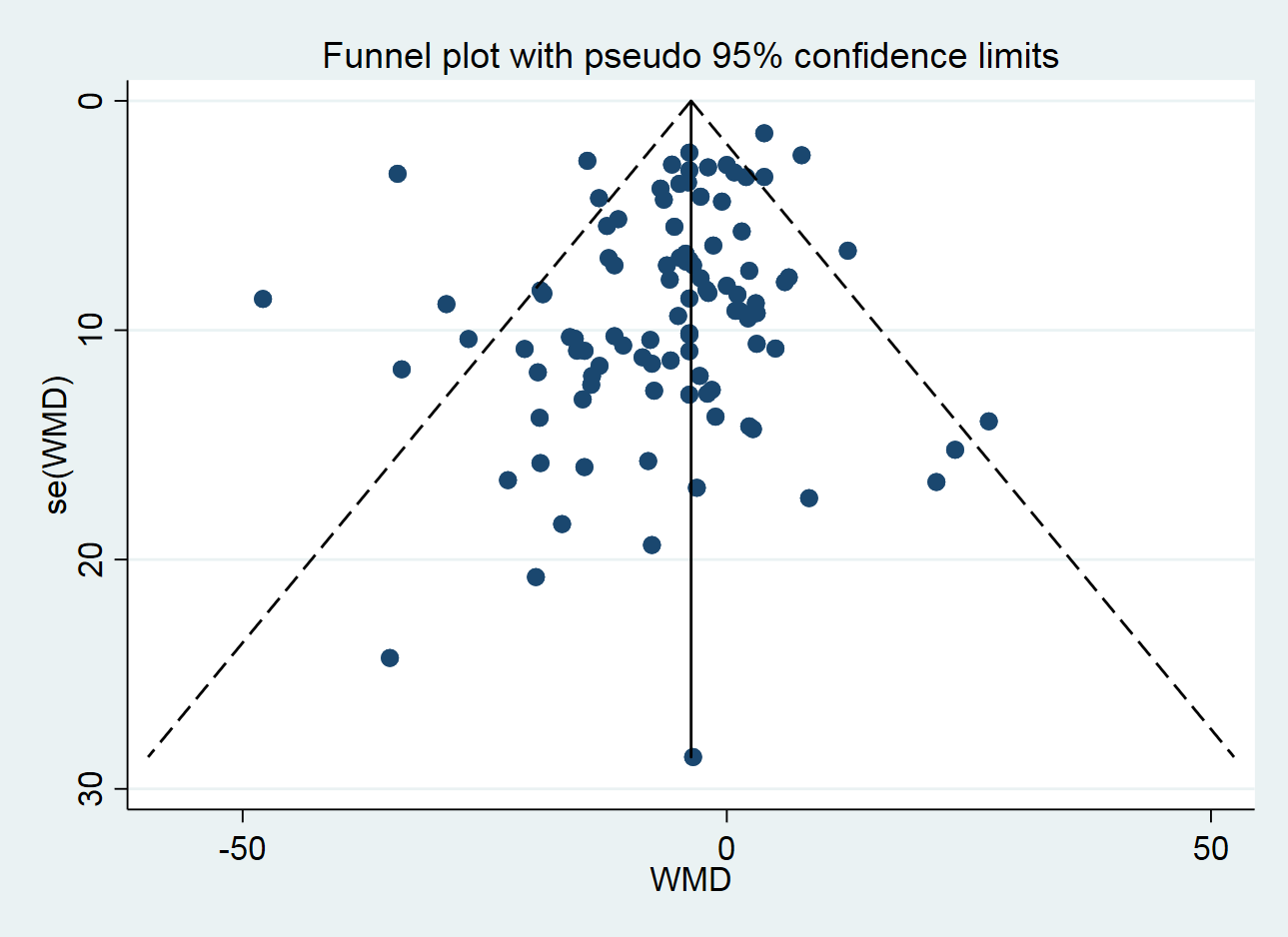


Figure S20. Low density lipoprotein funnel plot.


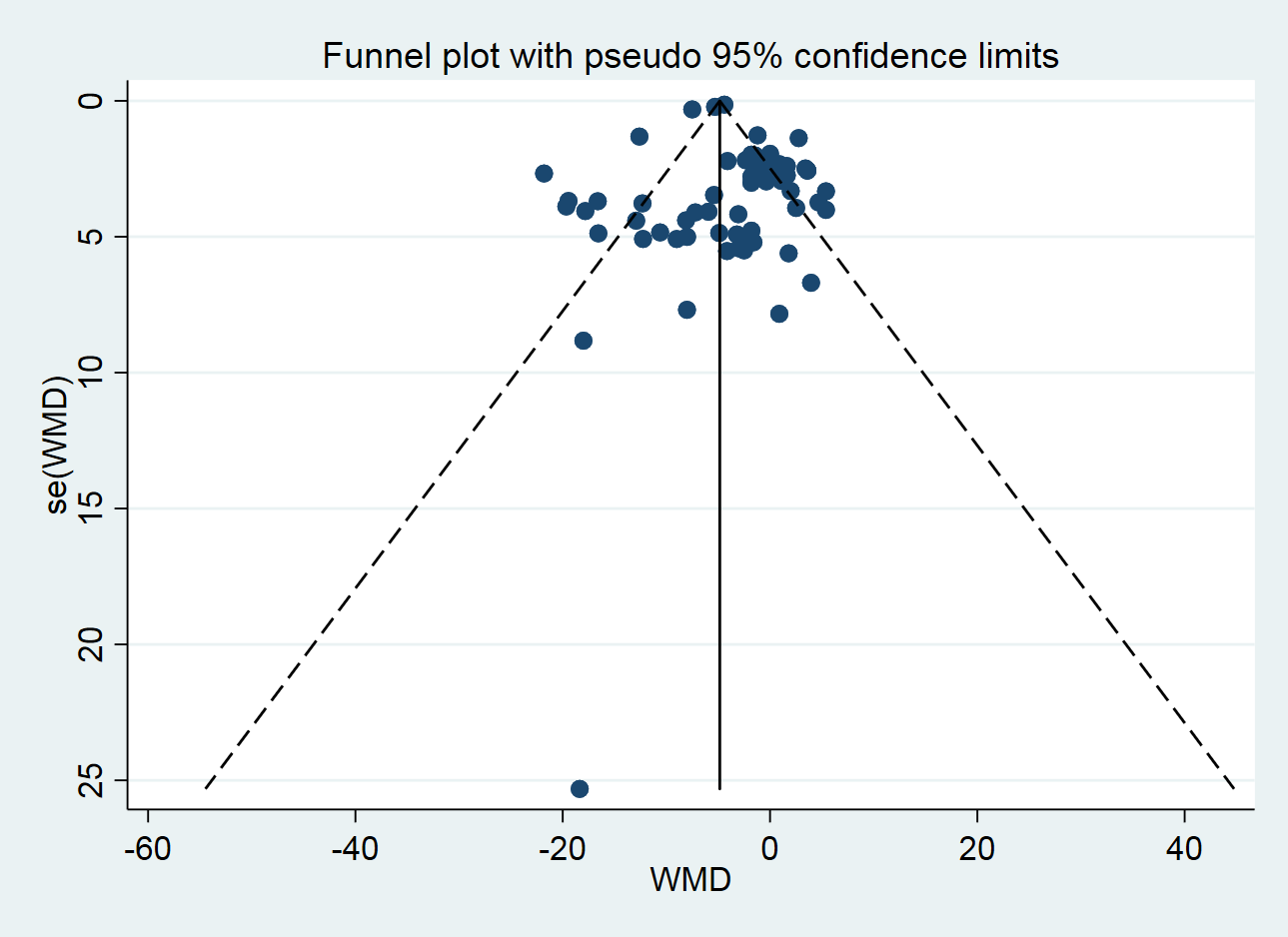


Figure S21. Blood glucose funnel plot.

## Longitudinal studies


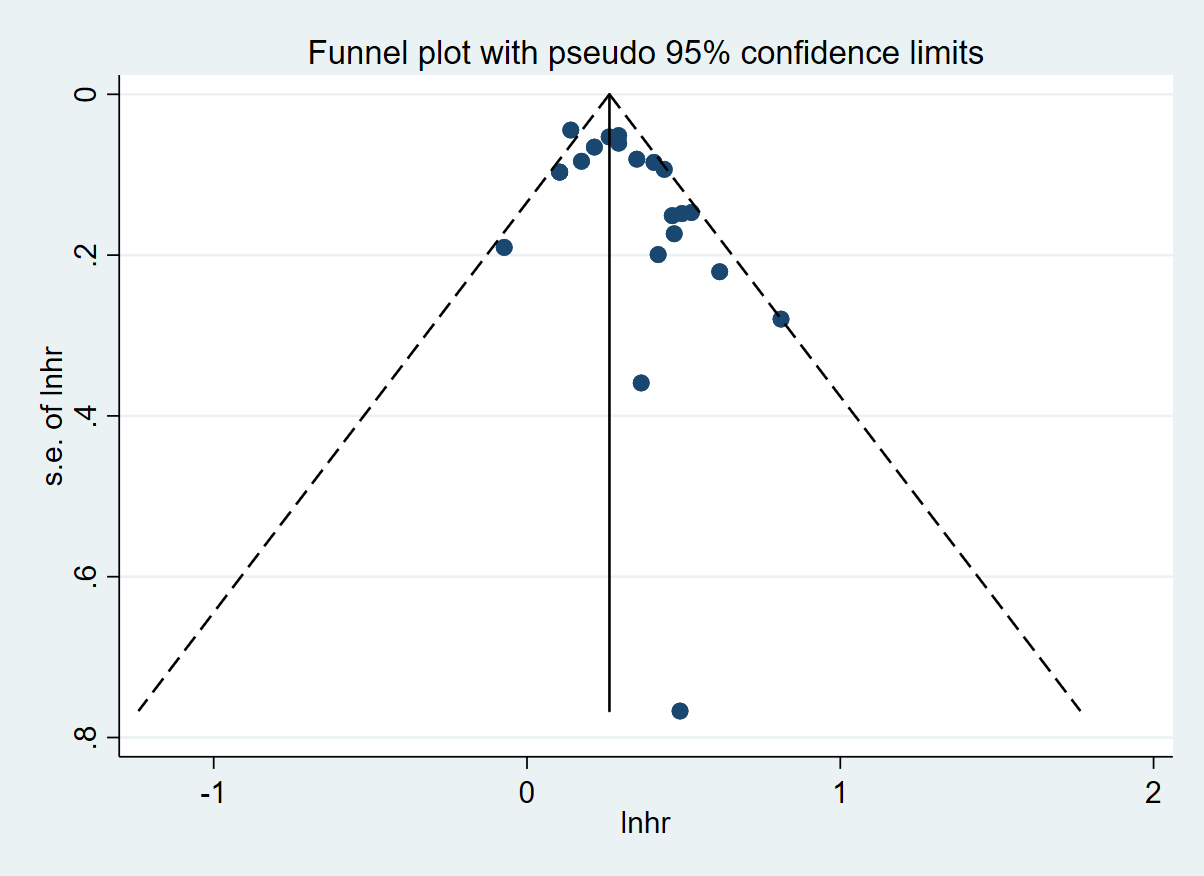


Figure S22. Sedentary behavior funnel plot.


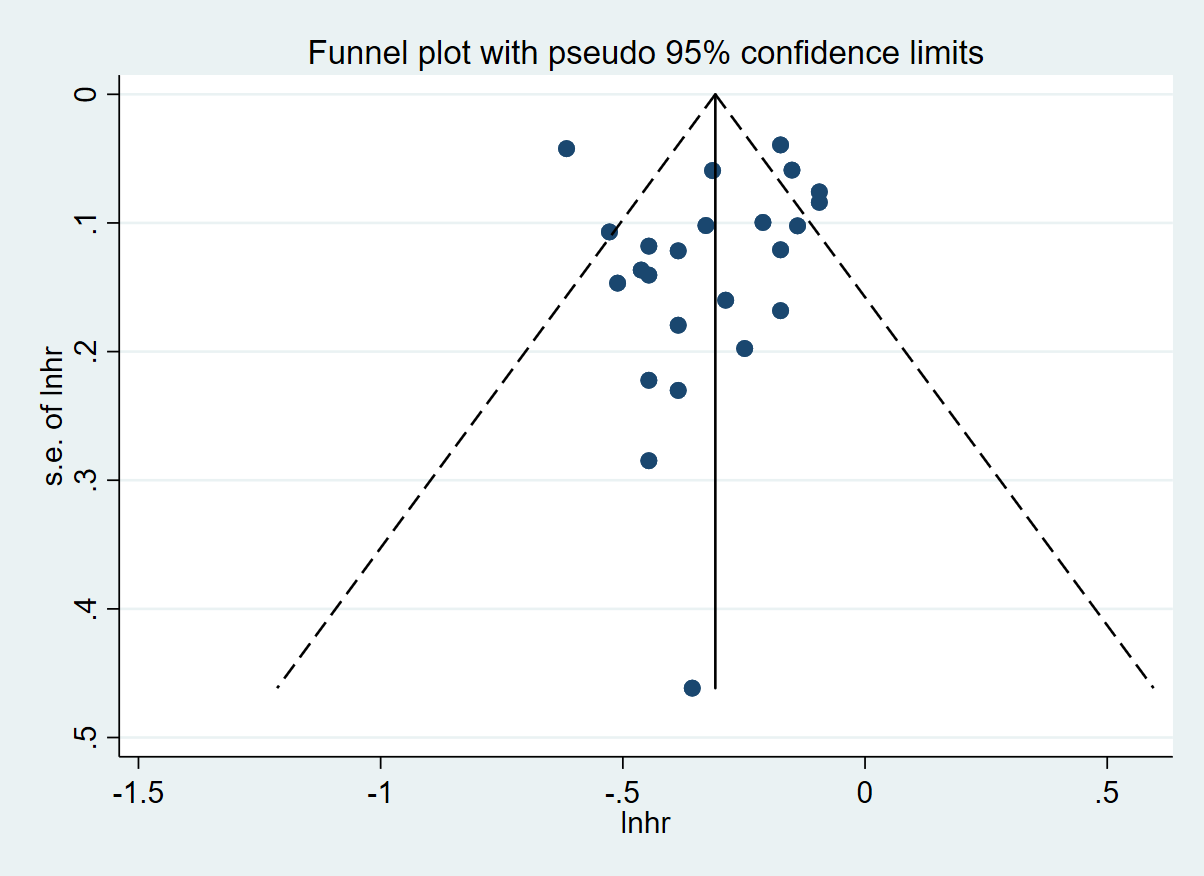


Figure S23. Physical activity funnel plot

# PRISMA 2020 Checklist

| **Section/topic** | **Item No** | **Checklist item** | **Reported on Page Number/Line Number** | **Reported on Section/Paragraph** |
| --- | --- | --- | --- | --- |
| **TITLE** | | | | |
| Title | 1 | Identify the report as a systematic review. | Page1/line1-2 | Title page |
| **ABSTRACT** | | | | |
| Abstract | 2 | See the PRISMA 2020 for Abstracts checklist (Table 2). | Page1-2/line4-41 | Abstract |
| **INTRODUCTION** | | | | |
| Rationale | 3 | Describe the rationale for the review in the context of existing knowledge. | Page4/line44-52 | Introduction/Paragraph1 |
| Objectives | 4 | Provide an explicit statement of the objective(s) or question(s) the review addresses. | Page4-5/line75-96 | Introduction/Paragraph3 |
| **METHODS** | | | | |
| Eligibility criteria | 5 | Specify the inclusion and exclusion criteria for the review and how studies were grouped for the syntheses. | Page5-6/line130-156 | Methods/Paragraph5-8 |
| Information sources | 6 | Specify all databases, registers, websites, organisations, reference lists and other sources searched or consulted to identify studies. Specify the date when each source was last searched or consulted. | Page4-5/line105-124 | Methods/Paragraph2-3 |
| Search strategy | 7 | Present the full search strategies for all databases, registers and websites, including any filters and limits used. | Page4-5/line105-113 | Methods/Paragraph2 |
| Selection process | 8 | Specify the methods used to decide whether a study met the inclusion criteria of the review, including how many reviewers screened each record and each report retrieved, whether they worked independently, and if applicable, details of automation tools used in the process. | Page5/line114-124 | Methods/Paragraph3 |
| Data collection process | 9 | Specify the methods used to collect data from reports, including how many reviewers collected data from each report, whether they worked independently, any processes for obtaining or confirming data from study investigators, and if applicable, details of automation tools used in the process. | Page5/line114-124 | Methods/Paragraph3 |
| Data items | 10a | List and define all outcomes for which data were sought. Specify whether all results that were compatible with each outcome domain in each study were sought (e.g. for all measures, time points, analyses), and if not, the methods used to decide which results to collect. | Page6/line163-171 | Methods/Paragraph9-10 |
|  | 10b | List and define all other variables for which data were sought (e.g. participant and intervention characteristics, funding sources). Describe any assumptions made about any missing or unclear information. | Page7/line183-186 | Methods/Paragraph12 |

| Study risk of bias assessment | 11 | Specify the methods used to assess risk of bias in the included studies, including details of the tool(s) used, how many reviewers assessed each study and whether they worked independently, and if applicable, details of automation tools used in the process. | Page7/line187-197 | Methods/Paragraph13-14 |
| --- | --- | --- | --- | --- |
| Effect measures | 12 | Specify for each outcome the effect measure(s) (e.g. risk ratio, mean difference) used in the synthesis or presentation of results. | Page8/line206-209 | Methods/Paragraph15 |
| Synthesis methods | 13a | Describe the processes used to decide which studies were eligible for each synthesis. | Page7/line201-204 | Methods/Paragraph15 |
|  | 13b | Describe any methods required to prepare the data for presentation or synthesis, such as handling of missing summary statistics, or data conversions. | Page7/line204-208 | Methods/Paragraph15 |
|  | 13c | Describe any methods used to tabulate or visually display results of individual studies and syntheses. | Page7/line213-214 | Methods/Paragraph16 |
|  | 13d | Describe any methods used to synthesize results and provide a rationale for the choice(s). If meta-analysis was performed, describe the model(s), method(s) to identify the presence and extent of statistical heterogeneity, and software package(s) used. | Page7-8/line204-217, 231-232 | Methods/Paragraph15-16 |
|  | 13e | Describe any methods used to explore possible causes of heterogeneity among study results. | Page8/line223-231 | Methods/Paragraph16 |
|  | 13f | Describe any sensitivity analyses conducted to assess robustness of the synthesized results. | Page8/line217-219 | Methods/Paragraph16 |
| Reporting bias assessment | 14 | Describe any methods used to assess risk of bias due to missing results in a synthesis (arising from reporting biases). | Page8/line220-221 | Methods/Paragraph16 |
| Certainty assessment | 15 | Describe any methods used to assess certainty (or confidence) in the body of evidence for an outcome. | Page7/line201-203 | Methods/Paragraph15 |
| **RESULTS** | | | | |
| Study selection | 16a | Describe the results of the search and selection process, from the number of records identified in the search to the number of studies included in the review, ideally using a flow diagram. | Page8/line236-246 | Results/Paragraph1 |
|  | 16b | Cite studies that met many but not all inclusion criteria (‘near-misses’) and explain why they were excluded. | Page8/line238-245 | Results/Paragraph1 |
| Study characteristics | 17 | Cite each included study and present its characteristics. | Page9/line247-257 | Results/Paragraph2 |
| Risk of bias in studies | 18 | Present assessments of risk of bias for each included study. | Page9/line263-270 | Results/Paragraph3-4 |
| Results of individual studies | 19 | For all outcomes, present, for each study: (a) summary statistics for each group (where appropriate) and (b) an effect estimate and its precision (e.g. confidence/credible interval), ideally using structured tables or plots. | Page9-12/line272-353 | Results/Paragraph5-12 |

| Results of syntheses | 20a | For each synthesis, briefly summarise the characteristics and risk of bias among contributing studies. | Page9-12/line272-353 | Results/Paragraph5-12 |
| --- | --- | --- | --- | --- |
|  | 20b | Present results of all statistical syntheses conducted. If meta-analysis was done, present for each the summary estimate and its precision (e.g. confidence/credible interval) and measures of statistical heterogeneity. If comparing groups, describe the direction of the effect. | Page9-12/line272-353 | Results/Paragraph5-12 |
|  | 20c | Present results of all investigations of possible causes of heterogeneity among study results. | Page10, 11/line290-303, 317-325 | Results/Paragraph6, 12 |
|  | 20d | Present results of all sensitivity analyses conducted to assess the robustness of the synthesized results. | Page12/line359-367 | Results/Paragraph13 |
| Reporting biases | 21 | Present assessments of risk of bias due to missing results (arising from reporting biases) for each synthesis assessed. | Page9/line263-270 | Results/Paragraph3-4 |
| Certainty of evidence | 22 | Present assessments of certainty (or confidence) in the body of evidence for each outcome assessed. | Page9/line263-270 | Results/Paragraph3-4 |
| **DISCUSSION** | | | | |
| Discussion | 23a | Provide a general interpretation of the results in the context of other evidence. | Page12/line341-347 | Discussion/Paragraph1 |
|  | 23b | Discuss any limitations of the evidence included in the review. | Page15-16/line479-487 | Discussion/Paragraph8 |
|  | 23c | Discuss any limitations of the review processes used. | Page15-16/line479-487 | Discussion/Paragraph8 |
|  | 23d | Discuss implications of the results for practice, policy, and future research. | Page15/line469-478 | Discussion/Paragraph7 |
| **OTHER INFORMATION** | | | | |
| Registration and protocol | 24a | Provide registration information for the review, including register name and registration number, or state that the review was not registered. | NA | NA |
|  | 24b | Indicate where the review protocol can be accessed, or state that a protocol was not prepared. | NA | NA |
|  | 24c | Describe and explain any amendments to information provided at registration or in the protocol. | NA | NA |
| Support | 25 | Describe sources of financial or non-financial support for the review, and the role of the funders or sponsors in the review. | NA | NA |
| Competing interests | 26 | Declare any competing interests of review authors. | NA | NA |
| Availability of data, code and other materials | 27 | Report which of the following are publicly available and where they can be found: template data collection forms; data extracted from included studies; data used for all analyses; analytic code; any other materials used in the review. | NA | NA |

# PRISMA 2020 for Abstracts checklist

| **Section/topic** | **Item No** | **Checklist item** | **Reported on Page Number/Line Number** | **Reported on Section/Paragraph** |
| --- | --- | --- | --- | --- |
| **TITLE** | | | | |
| Title | 1 | Identify the report as a systematic review. | Page1/line1-2 | Abstract/title |
| **BACKGROUND** | | | | |
| Objectives | 2 | Provide an explicit statement of the main objective(s) or question(s) the review addresses. | Page1/line4-7 | Abstract/Paragraph1 |
| **METHODS** | | | | |
| Eligibility criteria | 3 | Specify the inclusion and exclusion criteria for the review. | Page1/line11-14 | Abstract/Paragraph2 |
| Information sources | 4 | Specify the information sources (e.g. databases, registers) used to identify studies and the date when each was last searched. | Page1/line9-10 | Abstract/Paragraph2 |
| Risk of bias | 5 | Specify the methods used to assess risk of bias in the included studies. | Page1/line15-16 | Abstract/Paragraph2 |
| Synthesis of results | 6 | Specify the methods used to present and synthesize results. | Page1/line14 | Abstract/Paragraph2 |
| **RESULTS** | | | | |
| Included studies | 7 | Give the total number of included studies and participants and summarise relevant characteristics of studies. | Page1/line18-19 | Abstract/Paragraph3 |
| Synthesis of results | 8 | Present results for main outcomes, preferably indicating the number of included studies and participants for each. If meta-analysis was done, report the summary estimate and confidence/credible interval. If comparing groups, indicate the direction of the effect (i.e. which group is favoured). | Page1/line19-29 | Abstract/Paragraph3 |
| **DISCUSSION** | | | | |
| Limitations of evidence | 9 | Provide a brief summary of the limitations of the evidence included in the review (e.g. study risk of bias, inconsistency and imprecision). | Page1/line31-33 | Abstract/Paragraph4 |
| Interpretation | 10 | Provide a general interpretation of the results and important implications. | Page1/line33-34 | Abstract/Paragraph4 |
| **OTHER** | | | | |
| Funding | 11 | Specify the primary source of funding for the review. | NA | NA |
| Registration | 12 | Provide the register name and registration number. | NA | NA |

Please leave this space alone as it will be supplemented by the editorial office when needed.

# List of literature included in the Meta-analysis

1. Raz I, Rosenblit H, Kark JD. Effect of moderate exercise on serum lipids in young men with low high density lipoprotein cholesterol. *Arteriosclerosis (Dallas, Tex)*. May-Jun 1988;8(3):245-51. doi:10.1161/01.atv.8.3.245

2. Huttunen JK, Länsimies E, Voutilainen E, et al. Effect of moderate physical exercise on serum lipoproteins. A controlled clinical trial with special reference to serum high-density lipoproteins. *Circulation*. Dec 1979;60(6):1220-9. doi:10.1161/01.cir.60.6.1220

3. Miyachi M, Kawano H, Sugawara J, et al. Unfavorable effects of resistance training on central arterial compliance: a randomized intervention study. *Circulation*. Nov 2 2004;110(18):2858-63. doi:10.1161/01.Cir.0000146380.08401.99

4. Olson TP, Dengel DR, Leon AS, Schmitz KH. Moderate resistance training and vascular health in overweight women. *Med Sci Sports Exerc*. Sep 2006;38(9):1558-64. doi:10.1249/01.mss.0000227540.58916.0e

5. Ay A, Yurtkuran M. Influence of aquatic and weight-bearing exercises on quantitative ultrasound variables in postmenopausal women. *Am J Phys Med Rehabil*. Jan 2005;84(1):52-61. doi:10.1097/01.phm.0000146500.85850.be

6. Braith RW, Pollock ML, Lowenthal DT, Graves JE, Limacher MC. Moderate- and high-intensity exercise lowers blood pressure in normotensive subjects 60 to 79 years of age. *Am J Cardiol*. Jun 1 1994;73(15):1124-8. doi:10.1016/0002-9149(94)90294-1

7. Korshøj M, Ravn MH, Holtermann A, Hansen Å M, Krustrup P. Aerobic exercise reduces biomarkers related to cardiovascular risk among cleaners: effects of a worksite intervention RCT. *Int Arch Occup Environ Health*. Feb 2016;89(2):239-49. doi:10.1007/s00420-015-1067-5

8. Sillanpää E, Laaksonen DE, Häkkinen A, et al. Body composition, fitness, and metabolic health during strength and endurance training and their combination in middle-aged and older women. *Eur J Appl Physiol*. May 2009;106(2):285-96. doi:10.1007/s00421-009-1013-x

9. Connolly LJ, Scott S, Morencos CM, et al. Impact of a novel home-based exercise intervention on health indicators in inactive premenopausal women: a 12-week randomised controlled trial. *Eur J Appl Physiol*. Apr 2020;120(4):771-782. doi:10.1007/s00421-020-04315-7

10. Dağistan Akgöz A, Gözüm S. Effectiveness of a nurse-led physical activity intervention to decrease cardiovascular disease risk in middle-aged adults: A pilot randomized controlled study. *Journal of vascular nursing : official publication of the Society for Peripheral Vascular Nursing*. Sep 2020;38(3):140-148. doi:10.1016/j.jvn.2020.05.002

11. Pereira R, Krustrup P, Castagna C, et al. Effects of a 16-week recreational team handball intervention on aerobic performance and cardiometabolic fitness markers in postmenopausal women: A randomized controlled trial. *Prog Cardiovasc Dis*. Nov-Dec 2020;63(6):800-806. doi:10.1016/j.pcad.2020.10.005

12. Marcos-Pardo PJ, Orquin-Castrillón FJ, Gea-García GM, et al. Effects of a moderate-to-high intensity resistance circuit training on fat mass, functional capacity, muscular strength, and quality of life in elderly: A randomized controlled trial. *Sci Rep*. May 24 2019;9(1):7830. doi:10.1038/s41598-019-44329-6

13. Sillanpää E, Häkkinen A, Laaksonen DE, Karavirta L, Kraemer WJ, Häkkinen K. Serum basal hormone concentrations, nutrition and physical fitness during strength and/or endurance training in 39-64-year-old women. *Int J Sports Med*. Feb 2010;31(2):110-7. doi:10.1055/s-0029-1242811

14. Nielsen TT, Møller TK, Andersen LL, Zebis MK, Hansen PR, Krustrup P. Feasibility and Health Effects of a 15-Week Combined Exercise Programme for Sedentary Elderly: A Randomised Controlled Trial. *Biomed Res Int*. 2019;2019:3081029. doi:10.1155/2019/3081029

15. Vainionpää A, Korpelainen R, Kaikkonen H, Knip M, Leppäluoto J, Jämsä T. Effect of impact exercise on physical performance and cardiovascular risk factors. *Med Sci Sports Exerc*. May 2007;39(5):756-63. doi:10.1249/mss.0b013e318031c039

16. Trajković N, Sporiš G, Krističević T, Bogataj Š. Effects of Small-Sided Recreational Volleyball on Health Markers and Physical Fitness in Middle-Aged Men. *Int J Environ Res Public Health*. Apr 27 2020;17(9)doi:10.3390/ijerph17093021

17. Baker TT, Allen D, Lei KY, Willcox KK. Alterations in lipid and protein profiles of plasma lipoproteins in middle-aged men consequent to an aerobic exercise program. *Metabolism: clinical and experimental*. Nov 1986;35(11):1037-43. doi:10.1016/0026-0495(86)90040-5

18. Tseng ML, Ho CC, Chen SC, Huang YC, Lai CH, Liaw YP. A simple method for increasing levels of high-density lipoprotein cholesterol: a pilot study of combination aerobic- and resistance-exercise training. *Int J Sport Nutr Exerc Metab*. Jun 2013;23(3):271-81. doi:10.1123/ijsnem.23.3.271

19. Murphy MH, Murtagh EM, Boreham CA, Hare LG, Nevill AM. The effect of a worksite based walking programme on cardiovascular risk in previously sedentary civil servants [NCT00284479]. *BMC Public Health*. May 22 2006;6:136. doi:10.1186/1471-2458-6-136

20. Ho SS, Dhaliwal SS, Hills AP, Pal S. The effect of 12 weeks of aerobic, resistance or combination exercise training on cardiovascular risk factors in the overweight and obese in a randomized trial. *BMC Public Health*. Aug 28 2012;12:704. doi:10.1186/1471-2458-12-704

21. Tomeleri CM, Souza MF, Burini RC, et al. Resistance training reduces metabolic syndrome and inflammatory markers in older women: A randomized controlled trial. *Journal of diabetes*. Apr 2018;10(4):328-337. doi:10.1111/1753-0407.12614

22. Carral JMC, Rodríguez AL, Cardalda IM, Bezerra J. Muscle strength training program in nonagenarians - a randomized controlled trial. *Revista da Associacao Medica Brasileira (1992)*. Jul 22 2019;65(6):851-856. doi:10.1590/1806-9282.65.6.851

23. Grandjean PW, Oden GL, Crouse SF, Brown JA, Green JS. Lipid and lipoprotein changes in women following 6 months of exercise training in a worksite fitness program. *J Sports Med Phys Fitness*. Mar 1996;36(1):54-9.

24. Takeshima N, Rogers ME, Watanabe E, et al. Water-based exercise improves health-related aspects of fitness in older women. *Med Sci Sports Exerc*. Mar 2002;34(3):544-51. doi:10.1097/00005768-200203000-00024

25. Perez-Gomez J, Vicente-Rodríguez G, Ara Royo I, et al. Effect of endurance and resistance training on regional fat mass and lipid profile. *Nutr Hosp*. Mar-Apr 2013;28(2):340-6. doi:10.3305/nh.2013.28.2.6200

26. Vicente-Campos D, Mora J, Castro-Piñero J, González-Montesinos JL, Conde-Caveda J, Chicharro JL. Impact of a physical activity program on cerebral vasoreactivity in sedentary elderly people. *J Sports Med Phys Fitness*. Oct 2012;52(5):537-44.

27. Saremi A, Asghari M, Ghorbani A. Effects of aerobic training on serum omentin-1 and cardiometabolic risk factors in overweight and obese men. *J Sports Sci*. Jul 2010;28(9):993-8. doi:10.1080/02640414.2010.484070

28. Gelecek N, Ilçin N, Subaşi SS, Acar S, Demir N, Ormen M. The effects of resistance training on cardiovascular disease risk factors in postmenopausal women: a randomized-controlled trial. *Health Care Women Int*. 2012;33(12):1072-85. doi:10.1080/07399332.2011.645960

29. Tiainen S, Luoto R, Ahotupa M, Raitanen J, Vasankari T. 6-mo aerobic exercise intervention enhances the lipid peroxide transport function of HDL. *Free radical research*. 2016;50(11):1279-1285. doi:10.1080/10715762.2016.1252040

30. Bonfante IL, Chacon-Mikahil MP, Brunelli DT, et al. Combined training, FNDC5/irisin levels and metabolic markers in obese men: A randomised controlled trial. *Eur J Sport Sci*. Jun 2017;17(5):629-637. doi:10.1080/17461391.2017.1296025

31. Woolf-May K, Kearney EM, Jones DW, Davison RC, Coleman D, Bird SR. The effect of two different 18-week walking programmes on aerobic fitness, selected blood lipids and factor XIIa. *J Sports Sci*. Nov 1998;16(8):701-10. doi:10.1080/026404198366335

32. Jessup JV, Lowenthal DT, Pollock ML, Turner T. The effects of endurance exercise training on ambulatory blood pressure in normotensive older adults. *Geriatric nephrology and urology*. 1998;8(2):103-9. doi:10.1023/a:1008287320868

33. Ahmadizad S, Haghighi AH, Hamedinia MR. Effects of resistance versus endurance training on serum adiponectin and insulin resistance index. *Eur J Endocrinol*. Nov 2007;157(5):625-31. doi:10.1530/eje-07-0223

34. Gradidge PJ, Golele PN. Walking as a feasible means of effecting positive changes in BMI, waist, and blood pressure in black South African women. *African health sciences*. Dec 2018;18(4):917-921. doi:10.4314/ahs.v18i4.10

35. Allen NG, Higham SM, Mendham AE, Kastelein TE, Larsen PS, Duffield R. The effect of high-intensity aerobic interval training on markers of systemic inflammation in sedentary populations. *Eur J Appl Physiol*. Jun 2017;117(6):1249-1256. doi:10.1007/s00421-017-3613-1

36. Andersen E, Høstmark AT, Anderssen SA. Effect of a physical activity intervention on the metabolic syndrome in Pakistani immigrant men: a randomized controlled trial. *J Immigr Minor Health*. Oct 2012;14(5):738-46. doi:10.1007/s10903-012-9586-6

37. Andersen TR, Schmidt JF, Pedersen MT, Krustrup P, Bangsbo J. The Effects of 52 Weeks of Soccer or Resistance Training on Body Composition and Muscle Function in +65-Year-Old Healthy Males--A Randomized Controlled Trial. *PLoS One*. 2016;11(2):e0148236. doi:10.1371/journal.pone.0148236

38. Kin Isler A, Koşar SN, Korkusuz F. Effects of step aerobics and aerobic dancing on serum lipids and lipoproteins. *J Sports Med Phys Fitness*. Sep 2001;41(3):380-5.

39. Battaglia C, di Cagno A, Fiorilli G, et al. Benefits of selected physical exercise programs in detention: a randomized controlled study. *Int J Environ Res Public Health*. Oct 31 2013;10(11):5683-96. doi:10.3390/ijerph10115683

40. Bell GJ, Harber V, Murray T, Courneya KS, Rodgers W. A comparison of fitness training to a pedometer-based walking program matched for total energy cost. *J Phys Act Health*. Mar 2010;7(2):203-13. doi:10.1123/jpah.7.2.203

41. Prabhakaran B, Dowling EA, Branch JD, Swain DP, Leutholtz BC. Effect of 14 weeks of resistance training on lipid profile and body fat percentage in premenopausal women. *Br J Sports Med*. Jun 1999;33(3):190-5. doi:10.1136/bjsm.33.3.190

42. Yoshizawa M, Maeda S, Miyaki A, et al. Effect of 12 weeks of moderate-intensity resistance training on arterial stiffness: a randomised controlled trial in women aged 32-59 years. *Br J Sports Med*. Aug 2009;43(8):615-8. doi:10.1136/bjsm.2008.052126

43. Krustrup P, Nielsen JJ, Krustrup BR, et al. Recreational soccer is an effective health-promoting activity for untrained men. *Br J Sports Med*. Oct 2009;43(11):825-31. doi:10.1136/bjsm.2008.053124

44. Boardley D, Fahlman M, Topp R, Morgan AL, McNevin N. The impact of exercise training on blood lipids in older adults. *The American journal of geriatric cardiology*. Jan-Feb 2007;16(1):30-5. doi:10.1111/j.1076-7460.2007.05353.x

45. Brixius K, Schoenberger S, Ladage D, et al. Long-term endurance exercise decreases antiangiogenic endostatin signalling in overweight men aged 50-60 years. *Br J Sports Med*. Feb 2008;42(2):126-9; discussion 129. doi:10.1136/bjsm.2007.035188

46. Carvalho MJ, Marques E, Mota J. Training and detraining effects on functional fitness after a multicomponent training in older women. *Gerontology*. 2009;55(1):41-8. doi:10.1159/000140681

47. Chen N, Xia X, Qin L, et al. Effects of 8-Week Hatha Yoga Training on Metabolic and Inflammatory Markers in Healthy, Female Chinese Subjects: A Randomized Clinical Trial. *BioMed research international*. 2016;2016:5387258. doi:10.1155/2016/5387258

48. Chiu CH, Ko MC, Wu LS, et al. Benefits of different intensity of aerobic exercise in modulating body composition among obese young adults: a pilot randomized controlled trial. *Health Qual Life Outcomes*. Aug 24 2017;15(1):168. doi:10.1186/s12955-017-0743-4

49. Cho JK, Lee SH, Lee JY, Kang HS. Randomized controlled trial of training intensity in adiposity. *Int J Sports Med*. Jun 2011;32(6):468-75. doi:10.1055/s-0031-1271789

50. Conceição MS, Bonganha V, Vechin FC, et al. Sixteen weeks of resistance training can decrease the risk of metabolic syndrome in healthy postmenopausal women. *Clin Interv Aging*. 2013;8:1221-8. doi:10.2147/cia.S44245

51. Stensel DJ, Hardman AE, Brooke-Wavell K, et al. Brisk walking and serum lipoprotein variables in formerly sedentary men aged 42-59 years. *Clinical science (London, England : 1979)*. Dec 1993;85(6):701-8. doi:10.1042/cs0850701

52. Cunha PM, Ribeiro AS, Nunes JP, et al. Resistance training performed with single-set is sufficient to reduce cardiovascular risk factors in untrained older women: The randomized clinical trial. Active Aging Longitudinal Study. *Arch Gerontol Geriatr*. Mar-Apr 2019;81:171-175. doi:10.1016/j.archger.2018.12.012

53. Dalleck LC, Allen BA, Hanson BA, Borresen EC, Erickson ME, De Lap SL. Dose-response relationship between moderate-intensity exercise duration and coronary heart disease risk factors in postmenopausal women. *Journal of women's health (2002)*. Jan-Feb 2009;18(1):105-13. doi:10.1089/jwh.2008.0790

54. Delecluse C, Colman V, Roelants M, et al. Exercise programs for older men: mode and intensity to induce the highest possible health-related benefits. *Prev Med*. Oct 2004;39(4):823-33. doi:10.1016/j.ypmed.2004.03.023

55. Veríssimo MT, Aragão A, Sousa A, et al. Effect of physical exercise on lipid metabolism in the elderly. *Revista portuguesa de cardiologia : orgao oficial da Sociedade Portuguesa de Cardiologia = Portuguese journal of cardiology : an official journal of the Portuguese Society of Cardiology*. Oct 2002;21(10):1099-112.

56. Eguchi Y, Ohta M, Inoue T, et al. Effects of transitory stimulation interval exercise on physical function: a randomized controlled pilot study among Japanese Subjects. *Journal of UOEH*. Dec 1 2012;34(4):297-308. doi:10.7888/juoeh.34.297

57. Elliott KJ, Sale C, Cable NT. Effects of resistance training and detraining on muscle strength and blood lipid profiles in postmenopausal women. *Br J Sports Med*. Oct 2002;36(5):340-4. doi:10.1136/bjsm.36.5.340

58. Friedenreich CM, Neilson HK, Woolcott CG, et al. Changes in insulin resistance indicators, IGFs, and adipokines in a year-long trial of aerobic exercise in postmenopausal women. *Endocrine-related cancer*. Jun 2011;18(3):357-69. doi:10.1530/erc-10-0303

59. Fahlman M, Boardley D, Flynn MG, Braun WA, Lambert CP, Bouillon LE. Effects of endurance training on selected parameters of immune function in elderly women. *Gerontology*. Mar-Apr 2000;46(2):97-104. doi:10.1159/000022142

60. Fahlman MM, Boardley D, Lambert CP, Flynn MG. Effects of endurance training and resistance training on plasma lipoprotein profiles in elderly women. *The journals of gerontology Series A, Biological sciences and medical sciences*. Feb 2002;57(2):B54-60. doi:10.1093/gerona/57.2.b54

61. Frank P, Andersson E, Pontén M, Ekblom B, Ekblom M, Sahlin K. Strength training improves muscle aerobic capacity and glucose tolerance in elderly. *Scand J Med Sci Sports*. Jul 2016;26(7):764-73. doi:10.1111/sms.12537

62. Friedenreich CM, Woolcott CG, McTiernan A, et al. Adiposity changes after a 1-year aerobic exercise intervention among postmenopausal women: a randomized controlled trial. *International journal of obesity (2005)*. Mar 2011;35(3):427-35. doi:10.1038/ijo.2010.147

63. Hagerman FC, Walsh SJ, Staron RS, et al. Effects of high-intensity resistance training on untrained older men. I. Strength, cardiovascular, and metabolic responses. *The journals of gerontology Series A, Biological sciences and medical sciences*. Jul 2000;55(7):B336-46. doi:10.1093/gerona/55.7.b336

64. Rezende Barbosa MP, Vanderlei LC, Neves LM, et al. Functional training in postmenopause: Cardiac autonomic modulation and cardiorespiratory parameters, a randomized trial. *Geriatr Gerontol Int*. Aug 2019;19(8):823-828. doi:10.1111/ggi.13690

65. Monteiro MA, Gabriel RE, Neves ECM, Sousa MF, Abrantes JM, Moreira MH. Exercise effects in plantar pressure of postmenopausal women. *Menopause (New York, NY)*. Sep-Oct 2010;17(5):1017-25. doi:10.1097/gme.0b013e3181ddf6ef

66. Boyden TW, Pamenter RW, Going SB, et al. Resistance exercise training is associated with decreases in serum low-density lipoprotein cholesterol levels in premenopausal women. *Arch Intern Med*. Jan 11 1993;153(1):97-100.

67. Miyaki A, Maeda S, Choi Y, Akazawa N, Tanabe Y, Ajisaka R. Habitual aerobic exercise increases plasma pentraxin 3 levels in middle-aged and elderly women. *Appl Physiol Nutr Metab*. Oct 2012;37(5):907-11. doi:10.1139/h2012-069

68. Hespel P, Lijnen P, Fagard R, Van Hoof R, Rosseneu M, Amery A. Changes in plasma lipids and apoproteins associated with physical training in middle-aged sedentary men. *Am Heart J*. Apr 1988;115(4):786-92. doi:10.1016/0002-8703(88)90880-0

69. Heydari M, Boutcher YN, Boutcher SH. The effects of high-intensity intermittent exercise training on cardiovascular response to mental and physical challenge. *International journal of psychophysiology : official journal of the International Organization of Psychophysiology*. Feb 2013;87(2):141-6. doi:10.1016/j.ijpsycho.2012.11.013

70. Safarzade A, Alizadeh H, Bastani Z. The effects of circuit resistance training on plasma progranulin level, insulin resistance and body composition in obese men. *Hormone molecular biology and clinical investigation*. Mar 9 2020;41(2)doi:10.1515/hmbci-2019-0050

71. Ho SS, Radavelli-Bagatini S, Dhaliwal SS, Hills AP, Pal S. Resistance, aerobic, and combination training on vascular function in overweight and obese adults. *Journal of clinical hypertension (Greenwich, Conn)*. Dec 2012;14(12):848-54. doi:10.1111/j.1751-7176.2012.00700.x

72. Hunter GR, Wetzstein CJ, McLafferty CL, Jr., Zuckerman PA, Landers KA, Bamman MM. High-resistance versus variable-resistance training in older adults. *Med Sci Sports Exerc*. Oct 2001;33(10):1759-64. doi:10.1097/00005768-200110000-00022

73. Ihalainen JK, Schumann M, Eklund D, et al. Combined aerobic and resistance training decreases inflammation markers in healthy men. *Scand J Med Sci Sports*. Jan 2018;28(1):40-47. doi:10.1111/sms.12906

74. Lee YK, Cho SY, Roh HT. Effects of 16 Weeks of Taekwondo Training on the Cerebral Blood Flow Velocity, Circulating Neurotransmitters, and Subjective Well-Being of Obese Postmenopausal Women. *Int J Environ Res Public Health*. Oct 14 2021;18(20)doi:10.3390/ijerph182010789

75. Choi HM, Hurr C, Kim S. Effects of Elastic Band Exercise on Functional Fitness and Blood Pressure Response in the Healthy Elderly. *Int J Environ Res Public Health*. Sep 29 2020;17(19)doi:10.3390/ijerph17197144

76. Park W, Jung WS, Hong K, Kim YY, Kim SW, Park HY. Effects of Moderate Combined Resistance- and Aerobic-Exercise for 12 Weeks on Body Composition, Cardiometabolic Risk Factors, Blood Pressure, Arterial Stiffness, and Physical Functions, among Obese Older Men: A Pilot Study. *Int J Environ Res Public Health*. Oct 3 2020;17(19)doi:10.3390/ijerph17197233

77. Tanimoto M, Kawano H, Gando Y, et al. Low-intensity resistance training with slow movement and tonic force generation increases basal limb blood flow. *Clin Physiol Funct Imaging*. Apr 2009;29(2):128-35. doi:10.1111/j.1475-097X.2008.00847.x

78. Sillanpää E, Häkkinen A, Punnonen K, Häkkinen K, Laaksonen DE. Effects of strength and endurance training on metabolic risk factors in healthy 40-65-year-old men. *Scand J Med Sci Sports*. Dec 2009;19(6):885-95. doi:10.1111/j.1600-0838.2008.00849.x

79. Stensel DJ, Brooke-Wavell K, Hardman AE, Jones PR, Norgan NG. The influence of a 1-year programme of brisk walking on endurance fitness and body composition in previously sedentary men aged 42-59 years. *Eur J Appl Physiol Occup Physiol*. 1994;68(6):531-7. doi:10.1007/bf00599525

80. Sunami Y, Motoyama M, Kinoshita F, et al. Effects of low-intensity aerobic training on the high-density lipoprotein cholesterol concentration in healthy elderly subjects. *Metabolism: clinical and experimental*. Aug 1999;48(8):984-8. doi:10.1016/s0026-0495(99)90194-4

81. Murphy MH, Hardman AE. Training effects of short and long bouts of brisk walking in sedentary women. *Med Sci Sports Exerc*. Jan 1998;30(1):152-7. doi:10.1097/00005768-199801000-00021

82. Tsekouras YE, Magkos F, Kellas Y, Basioukas KN, Kavouras SA, Sidossis LS. High-intensity interval aerobic training reduces hepatic very low-density lipoprotein-triglyceride secretion rate in men. *American journal of physiology Endocrinology and metabolism*. Oct 2008;295(4):E851-8. doi:10.1152/ajpendo.90545.2008

83. Tsuzuku S, Kajioka T, Sakakibara H, Shimaoka K. Slow movement resistance training using body weight improves muscle mass in the elderly: A randomized controlled trial. *Scand J Med Sci Sports*. Apr 2018;28(4):1339-1344. doi:10.1111/sms.13039

84. Tully MA, Cupples ME, Hart ND, et al. Randomised controlled trial of home-based walking programmes at and below current recommended levels of exercise in sedentary adults. *J Epidemiol Community Health*. Sep 2007;61(9):778-83. doi:10.1136/jech.2006.053058

85. Werner TJ, Pellinger TK, Rosette VD, Ortlip AT. Effects of a 12-Week Resistance Training Program on Arterial Stiffness: A Randomized Controlled Trial. *Journal of strength and conditioning research*. Dec 1 2021;35(12):3281-3287. doi:10.1519/jsc.0000000000003331

86. Wong A, Figueroa A. Eight weeks of stretching training reduces aortic wave reflection magnitude and blood pressure in obese postmenopausal women. *J Hum Hypertens*. Apr 2014;28(4):246-50. doi:10.1038/jhh.2013.98

87. Wood PD, Haskell WL, Blair SN, et al. Increased exercise level and plasma lipoprotein concentrations: a one-year, randomized, controlled study in sedentary, middle-aged men. *Metabolism: clinical and experimental*. Jan 1983;32(1):31-9. doi:10.1016/0026-0495(83)90152-x

88. Woolf-May K, Kearney EM, Owen A, Jones DW, Davison RC, Bird SR. The efficacy of accumulated short bouts versus single daily bouts of brisk walking in improving aerobic fitness and blood lipid profiles. *Health Educ Res*. Dec 1999;14(6):803-15. doi:10.1093/her/14.6.803

89. Wooten JS, Phillips MD, Mitchell JB, et al. Resistance exercise and lipoproteins in postmenopausal women. *Int J Sports Med*. Jan 2011;32(1):7-13. doi:10.1055/s-0030-1268008

90. Zhang J, Chen G, Lu W, et al. Effects of physical exercise on health-related quality of life and blood lipids in perimenopausal women: a randomized placebo-controlled trial. *Menopause (New York, NY)*. Dec 2014;21(12):1269-76. doi:10.1097/gme.0000000000000264

91. Krustrup P, Hansen PR, Randers MB, et al. Beneficial effects of recreational football on the cardiovascular risk profile in untrained premenopausal women. *Scand J Med Sci Sports*. Apr 2010;20 Suppl 1:40-9. doi:10.1111/j.1600-0838.2010.01110.x

92. Bock BC, Dunsiger SI, Ciccolo JT, et al. Exercise Videogames, Physical Activity, and Health: Wii Heart Fitness: A Randomized Clinical Trial. *Am J Prev Med*. Apr 2019;56(4):501-511. doi:10.1016/j.amepre.2018.11.026

93. Nualnim N, Parkhurst K, Dhindsa M, Tarumi T, Vavrek J, Tanaka H. Effects of swimming training on blood pressure and vascular function in adults >50 years of age. *Am J Cardiol*. Apr 1 2012;109(7):1005-10. doi:10.1016/j.amjcard.2011.11.029

94. Hui SS, Xie YJ, Woo J, Kwok TC. Practicing Tai Chi had lower energy metabolism than walking but similar health benefits in terms of aerobic fitness, resting energy expenditure, body composition and self-perceived physical health. *Complement Ther Med*. Aug 2016;27:43-50. doi:10.1016/j.ctim.2016.05.006

95. Murtagh EM, Boreham CA, Nevill A, Hare LG, Murphy MH. The effects of 60 minutes of brisk walking per week, accumulated in two different patterns, on cardiovascular risk. *Prev Med*. Jul 2005;41(1):92-7. doi:10.1016/j.ypmed.2004.10.008

96. Tully MA, Cupples ME, Chan WS, McGlade K, Young IS. Brisk walking, fitness, and cardiovascular risk: a randomized controlled trial in primary care. *Prev Med*. Aug 2005;41(2):622-8. doi:10.1016/j.ypmed.2004.11.030

97. Gray SR, Baker G, Wright A, Fitzsimons CF, Mutrie N, Nimmo MA. The effect of a 12 week walking intervention on markers of insulin resistance and systemic inflammation. *Prev Med*. Jan 2009;48(1):39-44. doi:10.1016/j.ypmed.2008.10.013

98. Jaime SJ, Maharaj A, Alvarez-Alvarado S, Figueroa A. Impact of low-intensity resistance and whole-body vibration training on aortic hemodynamics and vascular function in postmenopausal women. *Hypertension research : official journal of the Japanese Society of Hypertension*. Dec 2019;42(12):1979-1988. doi:10.1038/s41440-019-0328-1

99. Church TS, Earnest CP, Skinner JS, Blair SN. Effects of different doses of physical activity on cardiorespiratory fitness among sedentary, overweight or obese postmenopausal women with elevated blood pressure: a randomized controlled trial. *Jama*. May 16 2007;297(19):2081-91. doi:10.1001/jama.297.19.2081

100. Houmard JA, Tanner CJ, Slentz CA, Duscha BD, McCartney JS, Kraus WE. Effect of the volume and intensity of exercise training on insulin sensitivity. *J Appl Physiol (1985)*. Jan 2004;96(1):101-6. doi:10.1152/japplphysiol.00707.2003

101. O'Donovan G, Owen A, Bird SR, et al. Changes in cardiorespiratory fitness and coronary heart disease risk factors following 24 wk of moderate- or high-intensity exercise of equal energy cost. *J Appl Physiol (1985)*. May 2005;98(5):1619-25. doi:10.1152/japplphysiol.01310.2004

102. Fatouros IG, Tournis S, Leontsini D, et al. Leptin and adiponectin responses in overweight inactive elderly following resistance training and detraining are intensity related. *The Journal of clinical endocrinology and metabolism*. Nov 2005;90(11):5970-7. doi:10.1210/jc.2005-0261

103. Vega-López S, Pignotti GA, Keller C, et al. Participation in a Social-Support Physical Activity Intervention Modestly Improves Lipoprotein Cholesterol Distribution Among Postpartum Sedentary Hispanic Women. *J Phys Act Health*. Sep 2015;12(9):1289-97. doi:10.1123/jpah.2014-0245

104. Musa DI, Adeniran SA, Dikko AU, Sayers SP. The effect of a high-intensity interval training program on high-density lipoprotein cholesterol in young men. *Journal of strength and conditioning research*. Mar 2009;23(2):587-92. doi:10.1519/JSC.0b013e318198fd28

105. Rossi FE, Fortaleza AC, Neves LM, et al. Combined Training (Aerobic Plus Strength) Potentiates a Reduction in Body Fat but Demonstrates No Difference on the Lipid Profile in Postmenopausal Women When Compared With Aerobic Training With a Similar Training Load. *Journal of strength and conditioning research*. Jan 2016;30(1):226-34. doi:10.1519/jsc.0000000000001020

106. Cunha PM, Tomeleri CM, Nascimento MA, et al. Comparision of Low and High Volume of Resistance Training on Body Fat and Blood Biomarkers in Untrained Older Women: A Randomized Clinical Trial. *Journal of strength and conditioning research*. Jan 1 2021;35(1):1-8. doi:10.1519/jsc.0000000000003245

107. Cao L, Jiang Y, Li Q, Wang J, Tan S. Exercise Training at Maximal Fat Oxidation Intensity for Overweight or Obese Older Women: A Randomized Study. *J Sports Sci Med*. Sep 2019;18(3):413-418.

108. Son WM, Park JJ. Resistance Band Exercise Training Prevents the Progression of Metabolic Syndrome in Obese Postmenopausal Women. *J Sports Sci Med*. Jun 2021;20(2):291-299. doi:10.52082/jssm.2021.291

109. Kim JW, Kim DY. Effects of aerobic exercise training on serum sex hormone binding globulin, body fat index, and metabolic syndrome factors in obese postmenopausal women. *Metabolic syndrome and related disorders*. Dec 2012;10(6):452-7. doi:10.1089/met.2012.0036

110. Knight E, Stuckey MI, Petrella RJ. Prescribing physical activity through primary care: does activity intensity matter? *Phys Sportsmed*. Sep 2014;42(3):78-89. doi:10.3810/psm.2014.09.2079

111. Lee JA, Kim JW, Kim DY. Effects of yoga exercise on serum adiponectin and metabolic syndrome factors in obese postmenopausal women. *Menopause (New York, NY)*. Mar 2012;19(3):296-301. doi:10.1097/gme.0b013e31822d59a2

112. Libardi CA, De Souza GV, Cavaglieri CR, Madruga VA, Chacon-Mikahil MP. Effect of resistance, endurance, and concurrent training on TNF-α, IL-6, and CRP. *Med Sci Sports Exerc*. Jan 2012;44(1):50-6. doi:10.1249/MSS.0b013e318229d2e9

113. Lovell DI, Cuneo R, Gass GC. Resistance training reduces the blood pressure response of older men during submaximum aerobic exercise. *Blood Press Monit*. Aug 2009;14(4):137-44. doi:10.1097/MBP.0b013e32832e0644

114. Medlow P, McEneny J, Murphy MH, Trinick T, Duly E, Davison GW. Exercise training protects the LDL I subfraction from oxidation susceptibility in an aged human population. *Atherosclerosis*. Apr 2015;239(2):516-22. doi:10.1016/j.atherosclerosis.2015.02.012

115. Moghadasi M, Mohebbi H, Rahmani-Nia F, Hassan-Nia S, Noroozi H, Pirooznia N. High-intensity endurance training improves adiponectin mRNA and plasma concentrations. *Eur J Appl Physiol*. Apr 2012;112(4):1207-14. doi:10.1007/s00421-011-2073-2

116. Morgan AL, Tobar DA, Snyder L. Walking toward a new me: the impact of prescribed walking 10,000 steps/day on physical and psychological well-being. *J Phys Act Health*. May 2010;7(3):299-307. doi:10.1123/jpah.7.3.299

117. Boutcher YN, Boutcher SH, Yoo HY, Meerkin JD. The Effect of Sprint Interval Training on Body Composition of Postmenopausal Women. *Med Sci Sports Exerc*. Jul 2019;51(7):1413-1419. doi:10.1249/mss.0000000000001919

118. Newton RL, Jr., Johnson WD, Larrivee S, et al. A Randomized Community-based Exercise Training Trial in African American Men: Aerobic Plus Resistance Training and Insulin Sensitivity in African American Men. *Med Sci Sports Exerc*. Feb 2020;52(2):408-416. doi:10.1249/mss.0000000000002149

119. Nelson ME, Fiatarone MA, Layne JE, et al. Analysis of body-composition techniques and models for detecting change in soft tissue with strength training. *The American journal of clinical nutrition*. May 1996;63(5):678-86. doi:10.1093/ajcn/63.5.678

120. Neves LM, Fortaleza AC, Rossi FE, et al. Functional training reduces body fat and improves functional fitness and cholesterol levels in postmenopausal women: a randomized clinical trial. *J Sports Med Phys Fitness*. Apr 2017;57(4):448-456. doi:10.23736/s0022-4707.17.06062-5

121. Niederseer D, Ledl-Kurkowski E, Kvita K, et al. Salzburg Skiing for the Elderly Study: changes in cardiovascular risk factors through skiing in the elderly. *Scand J Med Sci Sports*. Aug 2011;21 Suppl 1:47-55. doi:10.1111/j.1600-0838.2011.01341.x

122. Nikseresht M, Hafezi Ahmadi MR, Hedayati M. Detraining-induced alterations in adipokines and cardiometabolic risk factors after nonlinear periodized resistance and aerobic interval training in obese men. *Appl Physiol Nutr Metab*. Oct 2016;41(10):1018-1025. doi:10.1139/apnm-2015-0693

123. Nishida Y, Tanaka K, Hara M, et al. Effects of home-based bench step exercise on inflammatory cytokines and lipid profiles in elderly Japanese females: A randomized controlled trial. *Arch Gerontol Geriatr*. Nov-Dec 2015;61(3):443-51. doi:10.1016/j.archger.2015.06.017

124. McTiernan A, Sorensen B, Irwin ML, et al. Exercise effect on weight and body fat in men and women. *Obesity (Silver Spring, Md)*. Jun 2007;15(6):1496-512. doi:10.1038/oby.2007.178

125. Donnelly JE, Honas JJ, Smith BK, et al. Aerobic exercise alone results in clinically significant weight loss for men and women: midwest exercise trial 2. *Obesity (Silver Spring, Md)*. Mar 2013;21(3):E219-28. doi:10.1002/oby.20145

126. Okumiya K, Matsubayashi K, Wada T, Kimura S, Doi Y, Ozawa T. Effects of exercise on neurobehavioral function in community-dwelling older people more than 75 years of age. *J Am Geriatr Soc*. May 1996;44(5):569-72. doi:10.1111/j.1532-5415.1996.tb01444.x

127. Orsatti FL, Nahas EA, Maesta N, Nahas-Neto J, Burini RC. Plasma hormones, muscle mass and strength in resistance-trained postmenopausal women. *Maturitas*. Apr 20 2008;59(4):394-404. doi:10.1016/j.maturitas.2008.04.002

128. Patterson S, Pattison J, Legg H, Gibson AM, Brown N. The impact of badminton on health markers in untrained females. *J Sports Sci*. Jun 2017;35(11):1098-1106. doi:10.1080/02640414.2016.1210819

129. Probart CK, Notelovitz M, Martin D, Khan FY, Fields C. The effect of moderate aerobic exercise on physical fitness among women 70 years and older. *Maturitas*. Dec 1991;14(1):49-56. doi:10.1016/0378-5122(91)90147-i

130. Reichkendler MH, Rosenkilde M, Auerbach PL, et al. Only minor additional metabolic health benefits of high as opposed to moderate dose physical exercise in young, moderately overweight men. *Obesity (Silver Spring, Md)*. May 2014;22(5):1220-32. doi:10.1002/oby.20226

131. Stein RA, Michielli DW, Glantz MD, et al. Effects of different exercise training intensities on lipoprotein cholesterol fractions in healthy middle-aged men. *Am Heart J*. Feb 1990;119(2 Pt 1):277-83. doi:10.1016/s0002-8703(05)80017-1

132. Stavrinou PS, Bogdanis GC, Giannaki CD, Terzis G, Hadjicharalambous M. High-intensity Interval Training Frequency: Cardiometabolic Effects and Quality of Life. *Int J Sports Med*. Feb 2018;39(3):210-217. doi:10.1055/s-0043-125074

133. Takeshima N, Rogers ME, Islam MM, Yamauchi T, Watanabe E, Okada A. Effect of concurrent aerobic and resistance circuit exercise training on fitness in older adults. *Eur J Appl Physiol*. Oct 2004;93(1-2):173-82. doi:10.1007/s00421-004-1193-3

134. Connolly LJ, Bailey SJ, Krustrup P, Fulford J, Smietanka C, Jones AM. Effects of self-paced interval and continuous training on health markers in women. *Eur J Appl Physiol*. Nov 2017;117(11):2281-2293. doi:10.1007/s00421-017-3715-9

135. Gerage AM, Ritti-Dias RM, do Nascimento MA, et al. Chronic resistance training does not affect post-exercise blood pressure in normotensive older women: a randomized controlled trial. *Age (Dordrecht, Netherlands)*. Jun 2015;37(3):63. doi:10.1007/s11357-015-9801-1

136. Chung J, Kim K, Hong J, Kong HJ. Effects of prolonged exercise versus multiple short exercise sessions on risk for metabolic syndrome and the atherogenic index in middle-aged obese women: a randomised controlled trial. *BMC women's health*. Aug 22 2017;17(1):65. doi:10.1186/s12905-017-0421-z

137. Jang SH, Paik IY, Ryu JH, Lee TH, Kim DE. Effects of aerobic and resistance exercises on circulating apelin-12 and apelin-36 concentrations in obese middle-aged women: a randomized controlled trial. *BMC women's health*. Jan 29 2019;19(1):23. doi:10.1186/s12905-019-0722-5

138. Schmitz KH, Hannan PJ, Stovitz SD, Bryan CJ, Warren M, Jensen MD. Strength training and adiposity in premenopausal women: strong, healthy, and empowered study. *The American journal of clinical nutrition*. Sep 2007;86(3):566-72. doi:10.1093/ajcn/86.3.566

139. Schuit AJ, Schouten EG, Miles TP, Evans WJ, Saris WH, Kok FJ. The effect of six months training on weight, body fatness and serum lipids in apparently healthy elderly Dutch men and women. *International journal of obesity and related metabolic disorders : journal of the International Association for the Study of Obesity*. Sep 1998;22(9):847-53. doi:10.1038/sj.ijo.0800671

140. Seo DI, Jun TW, Park KS, Chang H, So WY, Song W. 12 weeks of combined exercise is better than aerobic exercise for increasing growth hormone in middle-aged women. *Int J Sport Nutr Exerc Metab*. Feb 2010;20(1):21-6. doi:10.1123/ijsnem.20.1.21

141. Trapp EG, Chisholm DJ, Freund J, Boutcher SH. The effects of high-intensity intermittent exercise training on fat loss and fasting insulin levels of young women. *International journal of obesity (2005)*. Apr 2008;32(4):684-91. doi:10.1038/sj.ijo.0803781

142. Timmons JF, Minnock D, Hone M, Cogan KE, Murphy JC, Egan B. Comparison of time-matched aerobic, resistance, or concurrent exercise training in older adults. *Scand J Med Sci Sports*. Nov 2018;28(11):2272-2283. doi:10.1111/sms.13254

143. Sousa N, Mendes R, Abrantes C, Sampaio J, Oliveira J. A randomized 9-month study of blood pressure and body fat responses to aerobic training versus combined aerobic and resistance training in older men. *Exp Gerontol*. Aug 2013;48(8):727-33. doi:10.1016/j.exger.2013.04.008

144. LeMura LM, von Duvillard SP, Andreacci J, Klebez JM, Chelland SA, Russo J. Lipid and lipoprotein profiles, cardiovascular fitness, body composition, and diet during and after resistance, aerobic and combination training in young women. *Eur J Appl Physiol*. Aug 2000;82(5-6):451-8. doi:10.1007/s004210000234

145. Santiago MC, Leon AS, Serfass RC. Failure of 40 weeks of brisk walking to alter blood lipids in normolipemic women. *Canadian journal of applied physiology = Revue canadienne de physiologie appliquee*. Dec 1995;20(4):417-28. doi:10.1139/h95-033

146. Matthews CE, Cohen SS, Fowke JH, et al. Physical activity, sedentary behavior, and cause-specific mortality in black and white adults in the Southern Community Cohort Study. *Am J Epidemiol*. Aug 15 2014;180(4):394-405. doi:10.1093/aje/kwu142

147. Evenson KR, Wen F, Herring AH. Associations of Accelerometry-Assessed and Self-Reported Physical Activity and Sedentary Behavior With All-Cause and Cardiovascular Mortality Among US Adults. *Am J Epidemiol*. Nov 1 2016;184(9):621-632. doi:10.1093/aje/kww070

148. Dohrn IM, Welmer AK, Hagströmer M. Accelerometry-assessed physical activity and sedentary time and associations with chronic disease and hospital visits - a prospective cohort study with 15 years follow-up. *The international journal of behavioral nutrition and physical activity*. Dec 9 2019;16(1):125. doi:10.1186/s12966-019-0878-2

149. Bjørk Petersen C, Bauman A, Grønbæk M, Wulff Helge J, Thygesen LC, Tolstrup JS. Total sitting time and risk of myocardial infarction, coronary heart disease and all-cause mortality in a prospective cohort of Danish adults. *The international journal of behavioral nutrition and physical activity*. Feb 5 2014;11:13. doi:10.1186/1479-5868-11-13

150. Matthews CE, George SM, Moore SC, et al. Amount of time spent in sedentary behaviors and cause-specific mortality in US adults. *The American journal of clinical nutrition*. Feb 2012;95(2):437-45. doi:10.3945/ajcn.111.019620

151. Seguin R, Buchner DM, Liu J, et al. Sedentary behavior and mortality in older women: the Women's Health Initiative. *Am J Prev Med*. Feb 2014;46(2):122-35. doi:10.1016/j.amepre.2013.10.021

152. Stamatakis E, Hamer M, Dunstan DW. Screen-based entertainment time, all-cause mortality, and cardiovascular events: population-based study with ongoing mortality and hospital events follow-up. *J Am Coll Cardiol*. Jan 18 2011;57(3):292-9. doi:10.1016/j.jacc.2010.05.065

153. Herber-Gast GC, Jackson CA, Mishra GD, Brown WJ. Self-reported sitting time is not associated with incidence of cardiovascular disease in a population-based cohort of mid-aged women. *The international journal of behavioral nutrition and physical activity*. May 7 2013;10:55. doi:10.1186/1479-5868-10-55

154. Bellettiere J, LaMonte MJ, Evenson KR, et al. Sedentary behavior and cardiovascular disease in older women: The Objective Physical Activity and Cardiovascular Health (OPACH) Study. *Circulation*. Feb 19 2019;139(8):1036-1046. doi:10.1161/circulationaha.118.035312

155. Chomistek AK, Manson JE, Stefanick ML, et al. Relationship of sedentary behavior and physical activity to incident cardiovascular disease: results from the Women's Health Initiative. *J Am Coll Cardiol*. Jun 11 2013;61(23):2346-54. doi:10.1016/j.jacc.2013.03.031

156. Garcia JM, Duran AT, Schwartz JE, et al. Types of Sedentary Behavior and Risk of Cardiovascular Events and Mortality in Blacks: The Jackson Heart Study. *J Am Heart Assoc*. Jul 2 2019;8(13):e010406. doi:10.1161/jaha.118.010406

157. Warren TY, Barry V, Hooker SP, Sui X, Church TS, Blair SN. Sedentary behaviors increase risk of cardiovascular disease mortality in men. *Med Sci Sports Exerc*. May 2010;42(5):879-85. doi:10.1249/MSS.0b013e3181c3aa7e

158. Katzmarzyk PT, Church TS, Craig CL, Bouchard C. Sitting time and mortality from all causes, cardiovascular disease, and cancer. *Med Sci Sports Exerc*. May 2009;41(5):998-1005. doi:10.1249/MSS.0b013e3181930355

159. Matthews CE, Moore SC, Sampson J, et al. Mortality Benefits for Replacing Sitting Time with Different Physical Activities. *Med Sci Sports Exerc*. Sep 2015;47(9):1833-40. doi:10.1249/mss.0000000000000621

160. Dohrn M, Sjöström M, Kwak L, Oja P, Hagströmer M. Accelerometer-measured sedentary time and physical activity—a 15 year follow-up of mortality in a Swedish population-based cohort. *Journal of science and medicine in sport*. 2018;21(7):702-707.

161. Young DR, Reynolds K, Sidell M, et al. Effects of physical activity and sedentary time on the risk of heart failure. *Circulation Heart failure*. Jan 2014;7(1):21-7. doi:10.1161/circheartfailure.113.000529

162. LaMonte MJ, Larson JC, Manson JE, et al. Association of Sedentary Time and Incident Heart Failure Hospitalization in Postmenopausal Women. *Circulation Heart failure*. Dec 2020;13(12):e007508. doi:10.1161/circheartfailure.120.007508

163. Jefferis BJ, Parsons TJ, Sartini C, et al. Does total volume of physical activity matter more than pattern for onset of CVD? A prospective cohort study of older British men. *Int J Cardiol*. Mar 1 2019;278:267-272. doi:10.1016/j.ijcard.2018.12.024

164. Mu X, Yu K, Long P, et al. Leisure-time physical activity and risk of incident cardiovascular disease in Chinese retired adults. *Sci Rep*. Dec 17 2021;11(1):24202. doi:10.1038/s41598-021-03475-6

165. Hermansen R, Jacobsen BK, Løchen ML, Morseth B. Leisure time and occupational physical activity, resting heart rate and mortality in the Arctic region of Norway: The Finnmark Study. *Eur J Prev Cardiol*. Oct 2019;26(15):1636-1644. doi:10.1177/2047487319848205

166. Stamatakis E, Hamer M, Lawlor DA. Physical activity, mortality, and cardiovascular disease: is domestic physical activity beneficial? The Scottish Health Survey -- 1995, 1998, and 2003. *Am J Epidemiol*. May 15 2009;169(10):1191-200. doi:10.1093/aje/kwp042

167. Rahman I, Bellavia A, Wolk A. Relationship between physical activity and heart failure risk in women. *Circulation Heart failure*. Nov 2014;7(6):877-81. doi:10.1161/circheartfailure.114.001467

168. Shiroma EJ, Sesso HD, Moorthy MV, Buring JE, Lee IM. Do moderate-intensity and vigorous-intensity physical activities reduce mortality rates to the same extent? *J Am Heart Assoc*. Oct 17 2014;3(5):e000802. doi:10.1161/jaha.114.000802

169. Schnohr P, O'Keefe JH, Lange P, Jensen GB, Marott JL. Impact of persistence and non-persistence in leisure time physical activity on coronary heart disease and all-cause mortality: The Copenhagen City Heart Study. *European journal of preventive cardiology*. Oct 2017;24(15):1615-1623. doi:10.1177/2047487317721021

170. Lachman S, Boekholdt SM, Luben RN, et al. Impact of physical activity on the risk of cardiovascular disease in middle-aged and older adults: EPIC Norfolk prospective population study. *Eur J Prev Cardiol*. Jan 2018;25(2):200-208. doi:10.1177/2047487317737628

171. Zhao H, Zhang XN, Shi Z, et al. Association of level of leisure-time physical activity with risks of all-cause mortality and cardiovascular disease in an elderly Chinese population: a prospective cohort study. *Journal of geriatric cardiology : JGC*. Oct 28 2020;17(10):628-637. doi:10.11909/j.issn.1671-5411.2020.10.003

172. Zhao M, Veeranki SP, Li S, Steffen LM, Xi B. Beneficial associations of low and large doses of leisure time physical activity with all-cause, cardiovascular disease and cancer mortality: a national cohort study of 88,140 US adults. *Br J Sports Med*. Nov 2019;53(22):1405-1411. doi:10.1136/bjsports-2018-099254

173. Borch KB, Braaten T, Lund E, Weiderpass E. Physical activity and mortality among Norwegian women - the Norwegian Women and Cancer Study. *Clinical epidemiology*. 2011;3:229-35. doi:10.2147/clep.S22681

174. Holtermann A, Schnohr P, Nordestgaard BG, Marott JL. The physical activity paradox in cardiovascular disease and all-cause mortality: the contemporary Copenhagen General Population Study with 104 046 adults. *Eur Heart J*. Apr 14 2021;42(15):1499-1511. doi:10.1093/eurheartj/ehab087

175. Ueshima K, Ishikawa-Takata K, Yorifuji T, et al. Physical activity and mortality risk in the Japanese elderly: a cohort study. *Am J Prev Med*. Apr 2010;38(4):410-8. doi:10.1016/j.amepre.2009.12.033

176. Inoue M, Iso H, Yamamoto S, et al. Daily total physical activity level and premature death in men and women: results from a large-scale population-based cohort study in Japan (JPHC study). *Ann Epidemiol*. Jul 2008;18(7):522-30. doi:10.1016/j.annepidem.2008.03.008

177. O'Donovan G, Lee IM, Hamer M, Stamatakis E. Association of "Weekend Warrior" and Other Leisure Time Physical Activity Patterns With Risks for All-Cause, Cardiovascular Disease, and Cancer Mortality. *JAMA Intern Med*. Mar 1 2017;177(3):335-342. doi:10.1001/jamainternmed.2016.8014

178. Besson H, Ekelund U, Brage S, et al. Relationship between subdomains of total physical activity and mortality. *Med Sci Sports Exerc*. Nov 2008;40(11):1909-15. doi:10.1249/MSS.0b013e318180bcad

179. Autenrieth CS, Baumert J, Baumeister SE, et al. Association between domains of physical activity and all-cause, cardiovascular and cancer mortality. *European journal of epidemiology*. Feb 2011;26(2):91-9. doi:10.1007/s10654-010-9517-6

180. Ataeinosrat A, Saeidi A, Abednatanzi H, Rahmani H, Daloii AA, Pashaei Z, et al. Intensity Dependent Effects of Interval Resistance Training on Myokines and Cardiovascular Risk Factors in Males With Obesity. Front Endocrinol (Lausanne). 2022;13:895512 https://doi.org/10.3389/fendo.2022.895512.

181. Okamoto T, Hashimoto Y. Decreases in Arterial Stiffness and Wave Reflection after Isometric Handgrip Training Are Associated with Improvements in Cognitive Function in Older Adults. Int J Environ Res Public Health. 2022;19(15) https://doi.org/10.3390/ijerph19159585.

182. Aibar-Almazán A, Martínez-Amat A, Cruz-Díaz D, Jesús de la Torre-Cruz M, Jiménez-García JD, Zagalaz-Anula N, et al. The Influence of Pilates Exercises on Body Composition, Muscle Strength, and Gait Speed in Community-Dwelling Older Women: A Randomized Controlled Trial. J Strength Cond Res. 2022;36(8):2298-305 https://doi.org/10.1519/jsc.0000000000003790.

183. Dos Santos M, Ferrari G, Lee DH, Rey-López JP, Aune D, Liao B, et al. Association of the "Weekend Warrior" and Other Leisure-time Physical Activity Patterns With All-Cause and Cause-Specific Mortality: A Nationwide Cohort Study. JAMA Intern Med. 2022;182(8):840-8 https://doi.org/10.1001/jamainternmed.2022.2488.

184. Lee DH, Rezende LFM, Joh HK, Keum N, Ferrari G, Rey-Lopez JP, et al. Long-Term Leisure-Time Physical Activity Intensity and All-Cause and Cause-Specific Mortality: A Prospective Cohort of US Adults. Circulation. 2022;146(7):523-34 https://doi.org/10.1161/circulationaha.121.058162.
