# Supplementary material for: Association between sedentary behavior, physical activity, and cardiovascular disease-related outcomes in adults—A meta-analysis and systematic review
Source: Front Public Health. 2022 Oct 19;10:1018460. doi: 10.3389/fpubh.2022.1018460 (PMC9632849; doi:10.3389/fpubh.2022.1018460)
Supplement: Supplementary file 2 [file Table_1.DOCX]

| Sources  **Table 1** Demographic characteristics of included studies | Study  design | Intervention type,  sample size, age(mean±SD/range), duration, | Time and  frequency  (per week) | Intervention time | Proportion of  female(%) | Whether  supervision | Region | Outcomes |
| --- | --- | --- | --- | --- | --- | --- | --- | --- |
| Raz et al, 1988 | RCT | AE=28, 24.70±0.80  CG=27, 25.00±0.80 | 55min×3 | 9weeks | 0 | Yes | Israel | BMI, TG, TC,  HDL, LDL |
| Huttunen et al, 1979 | RCT | MIE=44, 40.00-45.00  CG=46, 40.00-45.00 | 55min×3 | 4months | 0 | No | Finland | HDL |
| Motohiko et al, 2004 | RCT | RE=14, 22.00±3.74  CG=14, 22.00±3.74 | 45min×3 | 4months | 0 | Yes | Japan | SBP, DBP |
| Thomas et al, 2006 | RCT | RE=15, 38.00±7.75  CG=15, 39.00±7.75 | NR×2 | 1year | 100.0 | Yes | USA | BMI, TG, TC,  HDL, LDL, GLU |
| Alev et al, 2005 | RCT | Aquatic exercise=21, 54.28±6.08  Weight bearing exercise=21, 54.88±3.85  CG=20, 55.11±5.32 | 25-40min×3  25-40min×3 | 6months  6months | 100.0 | Yes | Turkey | BMI |
| Braithet al, 1994 | RCT | MIE=19, 66.00±5.00  HIE=14, 65.00±4.00  CG=11, 66.00±5.00 | 20-45min×3  20-35min×3 | 26weeks  26weeks | NR | NR | USA | SBP, DBP |
| Mette et al, 2016 | RCT | AE=57, 44.90±9.20  CG=59, 45.70±8.10 | 30min×NR | 16weeks | 75.9 | Yes | Denmark | TG, TC, HDL,  LDL |
| Elina et al, 2009 | RCT | EE=15, 51.70±6.90  Strength exercise=17, 50.80±7.90  Combined strength=18, 48.90±6.80  CG=12, 51.40±7.80 | 30-90min×2  60-90min×2  60-90min×2 | 21weeks  21weeks  21weeks | 100.0 | Yes | Finland | SBP, DBP, GLU |
| Luke et al, 2019  **Table 1** *(continued)* | RCT | Audio-visual-directed Exercise=12, 41.00±8.00  CG=12, 38.00±11.00 | 15min×3 | 12weeks | 100.0 | Yes | UK | BMI, SBP, DBP,  TG, TC, HDL, LDL,GLU |
| Sources  **Table 1** *(continued)* | Study  design | Intervention type,  sample size, age(mean±SD/range) | Time and  frequency  (per week) | Intervention  time | Proportion of  female(%) | Whether  supervision | Region | Outcomes |
| Ayşe et al, 2020 | RCT | Walking=9, 40.00-65.00  CG=9, 40.00-65.00 | 40-55min×5 | 12weeks | NR | NR | Turkey | BMI, SBP, DBP,  TC |
| Rita et al, 2020 | RCT | Recreational team handball=41, 68.00±6.00  CG=26, 68.00±6.00 | 60min×2-3 | 16weeks | 100.0 | NR | Portugal | TG, TC, HDL, LDL, GLU |
| Pablo et al, 2018 | RCT | Moderate-to-high intensity  Resistance circuit training=24, 69.00±3.20  CG=21, 70.00±4.10 | NR×3  NR×3 | 12weeks  12weeks | 40.0 | Yes | Spain | BMI |
| Sillanpää et al, 2010 | RCT | EE=21, 53.00±8.00  Strength exercise=27, 52.00±8.00  Combined exercise=22, 51.00±7.00  CG=9, 53.0±8.00 | 60-90min×NR  30-90min×NR  30-90min×NR | 21weeks  21weeks  21weeks | 100.0 | Yes | Holland | BMI |
| Tina-Thea et al, 2019 | RCT | AE=30, 70.00±6.00  CG=15, 71.00±6.00 | 90min×1 | 15weeks | 55.6 | Yes | Denmark | BMI, SBP, DBP,  TG, TC, HDL,  LDL |
| Aki et al, 2007 | RCT | Impact exercise=37, 35.00-40.00  CG=39, 35.00-40.00 | 60min×3 | 12months | 100.0 | Yes | Finland | BMI, TG, TC,  HDL, LDL, GLU |
| Nebojša et al, 2020 | RCT | Small-Sided recreational volleyball=12, 44.70±6.34  CG=12, 42.90±8.72 | 90min×3 | 10weeks | 0 | NR | Republic of Serbia | BMI, SBP, DBP,  TG, TC, HDL,  LDL, GLU |
| Baker et al, 1986  **Table 1** *(continued)* | RCT | AE=20, 58.20  CG=14, 58.20 | 48min×3 | 20weeks | 0 | Yes | USA | TC, HDL, LDL |
| Sources  **Table 1** *(continued)* | Study  design | Intervention type,  sample size, age(mean±SD/range) | Time and  frequency  (per week) | Intervention  time | Proportion of  female(%) | Whether  supervision | Region | Outcomes |
| Tseng et al, 2013 | RCT | AE=10, 22.20±2.21  RE=10, 22.10±3.48  Combined exercise=10, 21.30±1.90  CG=10, 22.30±3.16 | 60min×5  60min×3-5  60min×2-3 | 12weeks  12weeks | 0 | Yes | Taiwan | BMI, SBP, DBP,  GLU |
| Marie et al, 2006 | RCT | Walking=21, 41.40±7.50  CG=12, 40.80±10.00 | 25-35min×1-2 | 8weeks | NR | NR | UK | SBP, DBP, TG,  TC, HDL, LDL |
| Suleen et al, 2012 | RCT | AE=15, 55.00±4.65  RE=16, 52.00±4.40  Combination exercise=17, 53.00±5.36  CG=16, 52.00±7.20 | 30min×3-5  30min×3-5  30min×3-5 | 12weeks  12weeks  12weeks | 84.4 | NR | Australia | BMI, TG, TC,  HDL, LDL, GLU |
| Crisieli et al, 2018 | RCT | RE=22, 72.10±6.30  CG=23, 68.80±4.90 | NR×3 | 12weeks | 100.0 | Yes | Brazil | SBP, DBP, HDL, GLU |
| José et al, 2019 | RCT | Strength program with Thera Bands=11, 90.18±4.02  CG=13, 91.00±4.88 | 60min×2 | 12weeks | 100.0 | Yes | Spain | BMI |
| Grandjean et al, 1996 | RCT | AE=20, NR  CG=17, NR | 20-60min×3 | 6months | 100.0 | NR | USA | TG, TC, HDL,  LDL |
| Nobuo et al, 2002 | RCT | Well-rounded exercise=15, 69.30±4.50  CG=15, 69.30±3.30 | 70min×3 | 12weeks | 100.0 | Yes | Japan | TG, TC, HDL,  LDL |
| Jorge et al, 2013 | RCT | EE=7, 21.80±1.00  RE=8, 22.00±1.20  CG=11, 23.30±2.50 | 90min×NR  90min×NR | 10weeks  10weeks | 0 | Yes | Spain | BMI |
| Vicente et al, 2012  **Table 1** *(continued)* | RCT | PA=22, 64.00±4.00  CG=21, 64.00±2.00  **Table 1** *(continued)* | 60min×3-4 | 7months | 58.1 | NR | USA | SBP, DBP, TG,  TC, HDL, LDL |
| Sources | Study  design | Intervention type,  sample size, age(mean±SD/range) | Time and  frequency  (per week) | Intervention  time | Proportion of  female(%) | Whether  supervision | Region | Outcomes |
| Saremi et al, 2010 | RCT | AE=9, 43.10±4.70  CG=9, 43.10±4.70 | 50-60min×5 | 12weeks | 0 | NR | Iran | BMI, SBP, DBP,  TG, TC, HDL,  LDL, GLU |
| Nihal et al, 2012 | RCT | RE=24, 54.33±5.30  CG=19, 51.80±3.65 | 50-60min×3 | 12weeks | 100.0 | Yes | USA | BMI, SBP, DBP,  TG, TC, HDL,  LDL |
| Sanna et al, 2016 | RCT | AE=79, 54.50±3.50  CG=82, 54.10±3.70 | 60min×4 | 6months | 100.0 | No | Finland | TG, TC, HDL,  LDL |
| Ivan et al, 2017 | RCT | AE=12, 49.10±5.46  CG=10, 49.10±6.22 | 60min×3 | 4months | 0 | Yes | Brazil | BMI, TG, TC,  HDL, LDL, GLU |
| Woolf-May et al, 1998 | RCT | Long walking=16, 54.50±8.60  Repetitive short walking=16, 56.10±8.20  CG=16, 56.90±8.20 | 20-40min×7  10-15min×7 | 18weeks  18weeks | 42.9 | Yes | UK | TG, TC, LDL |
| Jessup et al, 1998 | RCT | EE=11, 67.80±4.40  CG=10, 69.20±5.10 | 45min×3 | 16weeks | 52.4 | Yes | USA | SBP, DBP |
| Sajad et al, 2007 | RCT | EE=8, 41.30±5.10  RE=8, 40.90±3.20  CG=8, 38.60±3.20 | 20-30min×3  56-60min×3 | 12weeks  12weeks | 0 | Yes | Iran | BMI |
| Philippe et al, 2018  **Table 1** *(continued)* | RCT | Walking=49, 37.40±8.78  CG=66, 44.40±11.50 | 30min×1 | 12weeks | 100.0 | Yes | South Africa | BMI, SBP |
| Sources  **Table 1** *(continued)* | Study  design | Intervention type,  sample size, age(mean±SD/range) | Time and  frequency  (per week) | Intervention  time | Proportion of  female(%) | Whether  supervision | Region | Outcomes |
| Nicholas et al, 2016 | RCT | HIIE=20, 35.00-60.00 Prolonged intermittent  sprint exercise=21, 35.00-60.00  CG=14, 35.00-60.00 | NR×3  NR×3 | 9weeks  9weeks | NR | Yes | Australia | BMI, SBP, DBP |
| Eivind et al, 2012 | RCT | PA=89, 35.70±6.10  CG=61, 39.70±9.20 | 60min×2 | 5months | 0 | Yes | Norway | SBP, DBP, TG,  HDL, LDL, GLU |
| Thomas et al, 2016 | RCT | Soccer exercise=9, 68.10±2.10  RE=9, 68.10±2.10  CG=8, 68.10±2.10 | 60-75min×2 | 52weeks | 0 | Yes | Denmark | BMI |
| Isler et al, 2001 | RCT | Step AE=15, 21.88±2.16  AE=15, 20.23±0.16  CG=15, 21.88±1.82 | 45min×3 | 8weeks | 100.0 | Yes | Turkey | TG, TC, HDL,  LDL |
| Claudia et al, 2013 | RCT | RE=21, 30.10±5.90  HIE=19, 33.90±6.80  CG=18, 32.80±8.90 | 45min×2  60min×2 | 9months  9months | 0 | Yes | Italy | BMI, SBP, DBP, TG, TC, HDL, LDL |
| Gordon et al, 2010 | RCT | Fitness exercise=40, 49.50±10.00  Walking program=43, 49.50±10.00  CG=45, 49.50±10.00 | 20-43min×3-4  NR×7 | 6months  6months | NR | Yes | Canada | SBP, DBP, TG,  TC, HDL, LDL,  GLU |
| Prabhakaran et al, 1999 | RCT | RE=12, 28.00±6.00  CG=12, 26.00±6.00 | 45-50min×3 | 14weeks | 100.0 | Yes | USA | TG, TC, HDL,  LDL |
| Yoshizawa et al, 2009  **Table 1** *(continued)* | RCT | RE=11, 47.00±6.63  AE=12, 47.00±6.93  CG=12, 49.00±10.39 | NR×2  30min×2 | 12weeks  12weeks | 100.0 | NR | Japan | SBP, DBP |
| Sources  **Table 1** *(continued)* | Study  design | Intervention type,  sample size, age(mean±SD/range) | Time and  frequency  (per week) | Intervention  time | Proportion of  female(%) | Whether  supervision | Region | Outcomes |
| Krustrup et al, 2008 | RCT | Soccer=13, 20.00-43.00  Running=12, 20.00-43.00  CG=11, 20.00-43.00 | 65min×2-3  NR×2-3 | 12weeks  12weeks | 0 | Yes | Denmark | BMI, SBP, DBP,  HDL, LDL |
| Debra et al, 2007 | RCT | RE=33, 75.30±6.00  AE=31, 74.10±6.20  Combined exercise=31, 73.20±6.60  CG=35, 75.90±7.70 | 45-50min×NR  45-50min×NR  45-50min×NR | 16weeks  16weeks  16weeks | 72.5 | No | USA | BMI, TG, TC,  HDL, LDL |
| Brixius et al, 2007 | RCT | Running=7, 50.00-60.00  Cycling=7, 50.00-60.00  CG=7, 50.00-60.00 | 60min×3  90min×3 | 6months  6months | 0 | NR | Germany | BMI |
| Gerontology et al, 2009 | RCT | AE=32, 68.40±2.93  CG=25, 69.60±4.20 | 60min×2 | 3months | 100.0 | Yes | Portugal | BMI |
| Chen et al, 2016 | RCT | Hatha yoga exercise=15, 18.00-25.00  CG=15, 18.00-25.00 | 60min×2 | 8weeks | 100.0 | Yes | China | BMI, SBP, DBP,  TG, TC, HDL,  LDL, GLU |
| Chiu et al, 2017 | RCT | Light-intensity exercise=12, 21.75±2.56  MIE=12, 20.92±1.32  HIE=12, 20.67±1.94  CG=12, 20.83±2.46 | 80min×3  80min×3  80min×3 | 12weeks  12weeks  12weeks | 29.2 | Yes | Taiwan | BMI |
| Cho et al, 2011  **Table 1** *(continued)* | RCT | Low-intensity exercise=13, 45.40±7.30  HIE=12, 45.40±7.30  CG=10, 45.40±7.30 | NR×3  NR×3 | 12weeks  12weeks | 100.0 | Yes | Korea | BMI, TG, HDL |
| Sources  **Table 1** *(continued)* | Study  design | Intervention type,  sample size, age(mean±SD/range) | Time and  frequency  (per week) | Intervention  time | Proportion of  female(%) | Whether  supervision | Region | Outcomes |
| Miguel et al, 2013 | RCT | RE=10, 53.40±3.95  CG=10, 53.00±5.70 | NR×3 | 16weeks | 100.0 | NR | Brazil | SBP, DBP, TG,  HDL, GLU |
| Stensel et al, 1993 | RCT | Brisk walking=42, 50.30±5.18  CG=23, 51.60±4.80 | 20-45min×2-3 | 1year | 0 | No | UK | TG, TC, HDL,  LDL |
| Paolo et al, 2019 | RCT | Single-set RT program=25, 71.40±5.71  CG=23, 69.04±4.45 | NR×3 | 12weeks | 100.0 | Yes | Brazil | TG, TC, HDL,  LDL, GLU |
| Lance et al, 2009 | RCT | 30-minute exercise time=8, 59.90±3.10  45-minute exercise time=8, 55.40±3.20  CG=10, 57.40±4.60 | 30min×3-4  45min×4-5 | 12weeks  12weeks | 100.0 | Yes | USA | BMI, SBP, DBP,  TG, TC, HDL,  LDL, GLU |
| Christophe et al, 2004 | RCT | EE and moderate-intensity RE=20, 63.80±4.80  EE and low-intensity=21, 63.70±6.00  RE=13, 64.50±5.30  CG=13, 61.50±5.00 | 24-35min×NR  24-35min×NR  24-35min×NR | 20weeks  20weeks  20weeks | 0 | Yes | Belgium | GLU |
| Manuel et al, 2002 | RCT | Combined exercise=31, 77.90±7.40  CG=32, 77.80±7.50 | 60min×3 | 8months | 50.8 | NR | Portugal | TG, TC, HDL,  LDL |
| Yasumasa et al, 2012 | RCT | Transitory stimulation interval  exercise=10, 50.80±11.20  MIE=10, 50.70±6.50  CG=10, 52.90±9.70 | 30min×3  30min×3 | 12weeks  12weeks | 100.0 | Yes | Japan | BMI, SBP, DBP,  TG, TC, HDL,  LDL |
| Elliott et al, 2002  **Table 1** *(continued)* | RCT | RE=8, 58.00±4.00  CG=7, 52.90±9.70 | NR×3 | 8weeks | 100.0 | Yes | UK | BMI, SBP, DBP,  TG, TC, HDL,  LDL |
| Sources  **Table 1** *(continued)* | Study  design | Intervention type,  sample size, age(mean±SD/range) | Time and  frequency  (per week) | Intervention  time | Proportion of  female(%) | Whether  supervision | Region | Outcomes |
| Christine et al, 2011 | RCT | AE=154, 61.20±5.40  CG=154, 60.60±5.70 | 45min×5 | 12months | 100.0 | Yes | Canada | GLU |
| Fahlman et al, 2000 | RCT | EE=15, 76.00±5.00  CG=14, 77.00±6.00 | 20-50min×3 | 10weeks | 100.0 | Yes | USA | BMI |
| Mariane et al, 2002 | RCT | EE=15, 76.00±5.00  RE=15, 73.00±3.00  CG=15, 74.00±5.00 | 20-50min×3  NR×3 | 10weeks  10weeks | 100.0 | NR | USA | TG, TC, HDL,  LDL |
| Frank et al, 2016 | RCT | EE=12, 71.40±3.81  CG=9, 72.00±4.20 | NR×3 | 8weeks | 50.0 | Yes | Sweden | BMI |
| Friedenreich et al, 2011 | RCT | AE=160, 61.20±5.40  CG=160, 60.60±5.70 | 45min×3 | 1year | 100.0 | Yes | Canada | BMI |
| Hagerman et al, 2000 | RCT | RE=9, 63.70±5.00  CG=9, 66.20±6.50 | NR×2 | 16weeks | 0 | Yes | USA | TG, TC, HDL,  LDL |
| Marianne et al, 2019 | RCT | Functional exercise=19, 60.00±4.50  CG=20, 58.45±4.80 | 18-30min×3 | 18weeks | 100.0 | NR | Brazil | SBP, DBP |
| Marco et al, 2010 | RCT | Combined exercise=60, 69.10±10.95  CG=61, 59.23±7.42 | 60min×3 | 1year | 100.0 | Yes | Portugal | BMI |
| Boyden et al, 1993 | RCT | RE=46, 34.1±2.92  CG=42, 34.5±29.8 | 60min×3 | 5months | 100.0 | Yes | USA | TG, TC, HDL,  LDL |
| Santiago et al, 1995  **Table 1** *(continued)* | RCT | Brisk walking=16, 30.10±5.30  CG=11, 31.50±6.10 | NR | 40weeks | 100.0 | Yes | USA | TG, TC, HDL,  LDL |
| Sources  **Table 1** *(continued)* | Study  design | Intervention type,  sample size, age(mean±SD/range) | Time and  frequency  (per week) | Intervention  time | Proportion of  female(%) | Whether  supervision | Region | Outcomes |
| Asako et al, 2012 | RCT | AE=11, 60.00±6.00  CG=11, 60.00±6.00 | 30-45min×3-5 | 2months | 100.0 | Yes | Japan | BMI, SBP, DBP,  TG, TC, HDL,  LDL |
| Hespel et al, 1988 | RCT | AE=13, 20.00-55.00  CG=14, 20.00-55.00 | 60min×3 | 16weeks | 0 | Yes | Holland | TG, TC, HDL,  LDL |
| Mehrdad et al, 2013 | RCT | HIIE=21, 18.00-35.00  CG=20, 18.00-35.00 | 25min×3 | 12weeks | 0 | Yes | Australia | BMI, SBP, DBP |
| Alireza et al, 2020 | RCT | RE=14, 36.00±7.70  CG=14, 36.00±7.70 | 80min×3 | 8weeks | 100.0 | Yes | Iran | BMI, GLU |
| Suleen et al, 2012 | RCT | RE=15, 55.00 AE=16, 52.00 Combination exercise=17, 53.00  CG=16, 52.00 | 30min×5  30min×5  30min×5 | 12weeks  12weeks  12weeks | NR | Yes | Australia | SBP, DBP |
| Hunter et al, 2001 | RCT | HIE=14, 67.30±4.70  RE=14, 65.90±4.30  CG=8, 65.90±4.00 | 45min×3  45min×3 | 25weeks  25weeks | 50.0 | Yes | USA | BMI |
| Ihalainen et al, 2018 | RCT | AE and RE in the same  training session=16, 31.00±6.00  AE and RE on alternating days=15, 31.00±6.00  CG=18, 31.00±6.00 | 30-50min×4-6  30-50min×4-6  30-50min×2-3 | 24weeks  24weeks  24weeks | 0 | Yes | Finland | BMI |
| Lee et al, 2021  **Table 1** *(continued)* | RCT | Taekwondo exercise=12, 56.00±2.90  CG=12, 57.50±2.90 | 60min×5 | 16weeks | 100.0 | Yes | Korea | BMI, TG, TC,  HDL, LDL |
| Sources  **Table 1** *(continued)* | Study  design | Intervention type,  sample size, age(mean±SD/range) | Time and  frequency  (per week) | Intervention  time | Proportion of  female(%) | Whether  supervision | Region | Outcomes |
| Choi et al, 2020 | RCT | Elastic band exercise=15, 75.10±5.42  CG=12, 72.30±4.85 | 60min×3 | 12weeks | NR | Yes | Korea | SBP, DBP |
| Wonil et al, 2020 | RCT | AE and RE=10, 69.10±0.90  CG=10, 69.10±0.90 | 90-120min×3 | 12weeks | 0 | Yes | Korea | BMI, SBP, DBP,  TG, TC, HDL,  LDL |
| Michiya et al, 2009 | RCT | Low-intensity RE=12, 19.00±0.69  Tonic force generation=12, 19.50±0.35  CG=12, 19.80±0.69 | NR×2  NR×2 | 13weeks  13weeks | NR | Yes | Japan | SBP, DBP, TG,  TC, HDL, LDL |
| Sillanpää et al, 2009 | RCT | EE=17, 52.60±7.90  Strength exercise=15, 54.10±6.00  Combined strength and EE=15, 56.30±6.80  CG=15, 53.80±7.70 | 30-90min×2  60-90min×2  60-90min×2 | 21weeks  21weeks  21weeks | 0 | Yes | Finland | BMI |
| Stensel et al, 1994 | RCT | Brisk walking=43, 50.30±5.25  CG=23, 51.60±4.80 | 20-45min×3-7 | 1year | 0 | No | UK | BMI |
| Sunami et al, 1999 | RCT | Low-intensity AE=20, 67.00  CG=20, 67.00 | 60min×2-4 | 5months | 50.0 | Yes | Japan | BMI, SBP, DBP,  TG, TC, HDL,  LDL |
| Murphy, 1998 | RCT | Brisk walking(long)=12, 44.80±8.40  CG=10, 47.30±4.10 | 30 min×5 | 10weeks | 100.0 | Yes | UK | SBP |
| Yiannis, 2008 | RCT | HIIE=7, 20.00-40.00  CG=8, 20.00-40.00 | 32 min×3 | 2months | 0 | Yes | Greece | BMI |
| Tsuzuku et al, 2018  **Table 1** *(continued)* | RCT | Slow movement RE=42, 72.50±2.10  CG=44, 73.20±2.10 | 15 min×7 | 12weeks | 40.7 | Yes | Japan | BMI |
| Sources  **Table 1** *(continued)* | Study  design | Intervention type,  sample size, age(mean±SD/range) | Time and  frequency  (per week) | Intervention  time | Proportion of  female(%) | Whether  supervision | Region | Outcomes |
| Mark et al, 2007 | RCT | Walking(five)=42, 47.80±5.97  CG=20, 49.05±6.31 | 30 min×5 | 12weeks | 66.1 | No | UK | BMI, SBP, DBP,  TG, TC, HDL,  LDL |
| Timothy et al, 2021 | RCT | RE(high-in)=10, 22.90±2.90  CG=10, 21.20±2.80 | NR min×2-3 | 12weeks | 0 | Yes | USA | BMI, SBP, DBP |
| Wong et al, 2014 | RCT | Stretching exercise=14, 56.00±3.74  CG=14, 57.00±3.74 | 50 min×3 | 8weeks | 100.0 | Yes | USA | SBP, DBP |
| Wood et al, 1983 | RCT | Running=48, 45.30  CG=33, 46.20 | 25 min×3-4 | 1year | 0 | Yes | USA | TG, TC, HDL,  LDL |
| Woolf-May et al, 1999 | RCT | Brisk walking(long)=18, 50.10±6.30  CG=13, 54.70±7.00 | 20-40 min×3-5 | 18weeks | 73.8 | NR | UK | TG, TC, HDL,  LDL |
| Wooten et al, 2011 | RCT | RE=9, 64.40±0.70  CG=12, 67.00±0.60 | 10 min×3 | 12weeks | 100.0 | Yes | USA | TG, TC, HDL,  LDL |
| Zhang et al, 2014 | RCT | Walking with strides=78, 47.82±4.58  CG=79, 48.64±5.24 | 30 min×3-7 | 12weeks | 100.0 | Yes | China | BMI, TG, TC,  HDL, LDL, GLU |
| Krustrup et al, 2009 | RCT | Soccer=21, 37.00±2.00  Running=18, 37.00±1.00  CG=14, 33.00±2.00 | 60 min×2  60 min×2 | 16weeks  16weeks | 100.0 | Yes | Denmark | BMI, SBP, DBP,  TG, TC, HDL,  LDL, GLU |
| Beth et al, 2018  **Table 1** *(continued)* | RCT | AE=93, 45.10±14.00  Videogames=96, 45.70±15.00  CG=94, 47.80±11.30 | 50 min×3  50 min×3 | 12weeks  12weeks | 78.8 | Yes | USA | TG, TC, HDL,  LDL |
| Sources  **Table 1** *(continued)* | Study  design | Intervention type,  sample size, age(mean±SD/range) | Time and  frequency  (per week) | Intervention  time | Proportion of  female(%) | Whether  supervision | Region | Outcomes |
| Nantinee et al, 2012 | RCT | Swimming exercise=24, 58.00±2.00  CG=19, 61.00±2.00 | 15-45 min×3-4 | 12weeks | 74.4 | Yes | USA | BMI, SBP, DBP,  TG, TC, HDL,  LDL, GLU |
| Stanley et al, 2016 | RCT | Tai Chi=129, 45.90±5.20  Walking=121, 46.60±4.90  CG=124, 44.90±5.60 | 45 min×5  45 min×5 | 12weeks  12weeks | 61.5 | Yes | Hong Kong | BMI |
| Elaine et al, 2005 | RCT | Walking=19, 45.70±9.40  CG=11, 45.70±9.40 | 20 min×3 | 12weeks | 66.7 | Yes | UK | SBP, DBP, TG,  TC, HDL, LDL |
| Tully et al, 2005 | RCT | Walking=21, 55.52±3.99  CG=10, 57.75±4.46 | 30 min×5 | 12weeks | 58.1 | No | UK | BMI, SBP, DBP,  TG, TC, HDL,  LDL |
| Stuart et al, 2009 | RCT | Gradually increasing walking=24, 48.10±9.20  CG=24, 51.30±8.30 | 30 min×5 | 12weeks | 77.1 | No | UK | BMI, SBP, DBP,  GLU |
| Salvador et al, 2019 | RCT | Whole-body vibration exercise=13, 64.00±1.00  Low-intensity RE=12, 64.00±1.00  CG=8, 67.00±1.00 | 20-30 min×NR  20-35 min×NR | 12weeks  12weeks | 100.0 | Yes | USA | SBP, DBP |
| Timothy et al, 2007 | RCT | AE=103, 56.60±6.60  CG=102, 57.20±5.80 | NR min×3-4 | 6months | 100.0 | Yes | USA | SBP, DBP, TG,  HDL, LDL, GLU |
| Joseph et al, 2004  **Table 1** *(continued)* | RCT | Low-volume/MIE=41, 53.10±0.90  Low-volume/HIE=30, 52.60±1.30  High-volume/HIE=43, 51.40±0.90  CG=40, 51.40±1.20 | NR  NR  NR | 6months  6months  6months | 41.2 | Yes | USA | BMI, GLU |
| Sources  **Table 1** *(continued)* | Study  design | Intervention type,  sample size, age(mean±SD/range) | Time and  frequency  (per week) | Intervention  time | Proportion of  female(%) | Whether  supervision | Region | Outcomes |
| Gary et al, 2005 | RCT | MIE=14, 41.00±3.00  HIE=13, 41.00±4.00  CG=15, 41.00±4.00 | NR min×3  NR min×3 | 24weeks  24weeks | 0 | Yes | UK | TG, TC, HDL,  LDL |
| Fatouros et al, 2005 | RCT | Low-intensity exercise=14, 71.10±3.60  MIE=12, 69.70±3.80  HIE=14, 70.80±2.80  CG=10, 69.80±5.10 | 60 min×3  60 min×3  60 min×3 | 6months  6months  6months | 0 | Yes | Greece | BMI, GLU |
| Sonia et al, 2015 | RCT | Walking=22, 28.70±5.40  CG=22, 27.60±5.80 | 30 min×5 | 12.0months | 100.0 | Yes | USA | SBP, DBP, TG,  TC, HDL, LDL,  GLU |
| Danladi et al, 2009 | RCT | HIIE=20, 29.80±4.50  CG=16, 29.40±4.90 | 40 min×3 | 8weeks | 0 | Yes | Nigeria | TC, HDL |
| Fabrício et al, 2016 | RCT | AE=15, 61.00±6.30  Combined exercise=37, 61.00±6.30  CG=18, 61.00±6.30 | 52 min×7  57 min×7 | 16weeks  16weeks | 100.0 | NR | Brazil | BMI, TC, HDL,  LDL |
| Paolo et al, 2021 | RCT | RE=18, 68.70±4.70  CG=18, 69.00±4.20 | 45-60 min×3 | 12weeks | 100.0 | Yes | Brazil | TG, TC, HDL,  LDL, GLU |
| Liquan et al, 2019 | RCT | AE=13, 63.80±5.90  CG=15, 64.00±4.60 | 20-40 min×3 | 12weeks | 100.0 | Yes | China | SBP, DBP, TG,  TC, HDL,  LDL |
| Won et al, 2021  **Table 1** *(continued)* | RCT | RE=18, 68.20±1.60  CG=17, 68.20±1.40 | 60 min×3 | 12weeks | 100.0 | Yes | Korea | BMI, SBP, DBP |
| Sources  **Table 1** *(continued)* | Study  design | Intervention type,  sample size, age(mean±SD/range) | Time and  frequency  (per week) | Intervention  time | Proportion of  female(%) | Whether  supervision | Region | Outcomes |
| Jong et al, 2012 | RCT | AE=15, 53.46±2.40  CG=15, 53.46±2.4 | 60 min×3 | 16weeks | 100.0 | Yes | Korea | BMI, SBP, DBP,  TG, TC, HDL,  LDL, GLU |
| Emily et al, 2014 | RCT | HIE=15, 63.00±5.00  CG=15, 62.00±5.00 | 10~ min×2~ | 12weeks | 60.0 | No | Canada | BMI, SBP, DBP,  TG, TC, HDL,  LDL, GLU |
| Jeong-Ah et al, 2012 | RCT | Yoga=8, 54.75±2.76  CG=8, 54.25±2.91 | 60 min×3 | 16weeks | 100.0 | Yes | Korea | BMI, SBP, DBP,  TG, TC, HDL,  LDL, GLU |
| Cleiton et al, 2012 | RCT | RE=11, 49.27±4.81  EE=12, 49.25±5.42  Concurrent exercise=11, 48.54±5.35  CG=13, 49.10±5.78 | NR min×3  60 min×3  60 min×3 | 16weeks  16weeks  16weeks | 0 | No | Brazil | BMI, TG, TC,  HDL, LDL, GLU |
| Dale et al, 2009 | RCT | RE=12, 74.10±2.70  CG=12, 73.50±3.30 | 25 min×3 | 16weeks | 0 | Yes | Australia | SBP, DBP |
| Paul et al, 2015 | RCT | MIE=6, 21.00±2.00  CG=6, 21.00±2.00 | NR min×4 | 12weeks | 0 | No | UK | BMI, SBP, DBP,  TG, TC |
| Mehrzad et al, 2012 | RCT | EE=8, 41.18±6.10  CG=8, 41.18±6.10 | 45 min×4 | 12weeks | 0 | Yes | Iran | BMI, GLU |
| Amy et al, 2010  **Table 1** *(continued)* | RCT | Walking=14, 57.40±6.52  CG=15, 62.10±3.99 | NR | 12weeks | 75.9 | No | USA | TC, HDL |
| Sources  **Table 1** *(continued)* | Study  design | Intervention type,  sample size, age(mean±SD/range) | Time and  frequency  (per week) | Intervention  time | Proportion of  female(%) | Whether  supervision | Region | Outcomes |
| Yati et al, 2019 | RCT | Sprint interval exercise=20, 54.10±3.60  CG=20, 53.30±3.40 | 30 min×3 | 8weeks | 100.0 | Yes | Australia | BMI, SBP, DBP,  TG, TC, HDL,  LDL, GLU |
| Robert et al, 2020 | RCT | AE and RE=51, 50.10±9.60  CG=49, 53.70±8.00 | 20-30 min×2 | 5months | 0 | No | USA | SBP, DBP, TG,  TC, HDL, LDL,  GLU |
| Nelson et al, 1996 | RCT | Strength-exercise=20, 61.10±0.80  CG=19, 57.30±1.40 | NR min×2 | 52weeks | 100.0 | NR | USA | BMI |
| Lucas et al, 2017 | RCT | Functional exercise=28, 58.60±3.90  CG=22, 57.70±4.80 | 18-30 min×3 | 16weeks | 100.0 | Yes | Brazil | BMI, TC, HDL,  LDL, GLU |
| Niederseer et al, 2011 | RCT | Skiing=22, 66.60±2.10  CG=20, 67.30±4.40 | 210 min×3 | 12weeks | 47.6 | Yes | Austria | BMI, SBP, DBP,  TG, TC, HDL,  LDL |
| Mahmoud et al, 2016 | RCT | Nonlinear RE=12, 40.40±5.20  AIE=10, 39.60±3.70  CG=11, 38.90±5.90 | 55 min×3  21 min×3 | 12weeks  12weeks | 0 | Yes | Iran | TG, TC, HDL,  LDL, GLU |
| Yuichiro et al, 2015 | RCT | Bench step exercise=31, 70.40±5.80  CG=31, 69.70±6.60 | NR | 12weeks | 100.0 | Yes | Japan | BMI, SBP, DBP,  TG, TC, HDL,  LDL |
| Anne et al, 2007 | RCT | Moderate-to-vigorous AE=49, 54.40±7.10  CG=51, 53.70±5.60 | 60 min×6 | 12months | 49.5 | Yes | USA | BMI |
| Joseph et al, 2013  **Table 1** *(continued)* | RCT | AE=18, 22.60±3.20  CG=9, 21.80±2.60 | NR min×5 | 10months | 49.1 | Yes | USA | BMI |
| Sources  **Table 1** *(continued)* | Study  design | Intervention type,  sample size, age(mean±SD/range) | Time and  frequency  (per week) | Intervention  time | Proportion of  female(%) | Whether  supervision | Region | Outcomes |
| Okumiya et al, 1996 | RCT | AE=21, 78.70±4.60  CG=21, 78.90±4.70 | 60 min×2 | 6months | 57.1 | Yes | Japan | BMI, SBP, DBP |
| Fabio et al, 2008 | RCT | RE=21, 57.80±8.00  CG=22, 59.30±6.20 | 50-60 min×3 | 16weeks | 100.0 | Yes | Brazil | BMI |
| Stephen et al, 2017 | RCT | Badminton=13, 34.30±6.90,  Running=12, 34.30±6.90  CG=8, 34.30±6.90 | 60 min×3  60 min×3 | 8weeks  8weeks | 100.0 | Yes | UK | BMI, SBP, DBP,  TG, TC, HDL,  LDL |
| Probart et al, 1991 | RCT | AE=10, 72.00±0.60  CG=6, 72.00±0.68 | 20 min×3 | 6months | 100.0 | Yes | USA | BMI |
| Reichkendler et al, 2014 | RCT | AE=22, 28.00±1.00  CG=18, 31.00±1.00 | NR min× | 10weeks | 0 | No | Denmark | BMI, TG, TC,  HDL, LDL |
| Stein et al, 1990 | RCT | HIE=12, 44.00±8.00  CG=10, 44.00±8.00 | 30 min×3 | 12weeks | 0 | Yes | USA | TG, TC, HDL,  LDL |
| Pinelopi et al, 2018 | RCT | HIIE=13, 31.90±2.40  CG=8, 31.70±0.80 | 31 min×3-4 | 8weeks | 47.6 | NR | Cyprus | BMI, TG, TC,  HDL, LDL, GLU |
| Nobuo et al, 2004 | RCT | AE=18, 68.30±4.90  RE=17, 68.00±3.40 | 50 min×3 | 12weeks | 57.1 | Yes | Japan | TG, TC, HDL,  LDL |
| Luke et al, 2017 | RCT | HIIE=15, 44.00±7.00  Continuous exercise=15, 43.00±7.00  CG=15, 45.00±7.00 | 35 min×3  50 min×3 | 12weeks  12weeks | 100.0 | Yes | UK | BMI, SBP, DBP,  TG, TC, HDL,  LDL, GLU |
| Aline et al, 2015  **Table 1** *(continued)* | RCT | RE=14, 65.50±5.00  CG=14, 66.20±4.10 | 25-30 min×3 | 12weeks | 100.0 | Yes | Brazil | BMI |
| Sources  **Table 1** *(continued)* | Study  design | Intervention type,  sample size, age(mean±SD/range) | Time and  frequency  (per week) | Intervention time | Proportion of  female(%) | Whether  supervision | Region | Outcomes |
| Wook et al, 2017 | RCT | Treadmill exercise=12, 49.33±5.06  CG=12, 49.55±4.29 | 30 min×3 | 12weeks | 100.0 | Yes | Korea | BMI, SBP, DBP,  TG, TC, HDL,  LDL, GLU |
| Sun-Hwa et al, 2019 | RCT | AE=8, 56.62±4.02  RE=8, 54.62±4.02  CG=8, 55.14±3.27 | 50 min×4  50 min×4 | 8weeks  8weeks | 100.0 | Yes | Korea | BMI |
| Kathryn et al, 2007 | RCT | Strength exercise=70, 37.00±5.00  CG=63, 36.00±6.00 | NR min×4 | 2years | 100.0 | Yes | USA | BMI |
| Schuit et al, 1998 | RCT | Cycle ergometer Exercise=66, 68.20±5.30  All-round activity=33, 65.20±4.30  CG=82, 69.40±5.50 | 60 min×3  45 min×3 | 6months  6months | NR | Yes | Holland | TG, TC, HDL,  LDL |
| Dong et al, 2010 | RCT | AE=7, 55.00±4.80  RE=8, 54.00±3.60  CG=7, 58.00±4.20 | 30 min×4  45 min×3 | 12weeks  12weeks | 100.0 | Yes | Korea | BMI, SBP, DBP,  TG, HDL, GLU |
| Trapp et al, 2008 | RCT | HIIE=15, 22.40±0.70  Steady-state exercise=15, 21.00±0.80  CG=15, 22.00±0.10 | 35 min×3  40 min×3 | 15weeks  15weeks | 100.0 | Yes | Australia | GLU |
| James et al, 2018  **Table 1** *(continued)* | RCT | AE=21, 69.20±3.10  RE=21, 69.60±4.90  AE and RE=21, 69.20±2.70  CG=21, 69.0±3.30 | 40 min×3  40 min×3  40 min×3 | 12weeks  12weeks  12weeks | 46.4 | Yes | Ireland | SBP, DBP,  GLU |
| Sources  **Table 1** *(continued)* | Study  design | Intervention type,  sample size, age(mean±SD/range) | Time and  frequency  (per week) | Intervention  time | Proportion of  female(%) | Whether  supervision | Region | Outcomes |
| Nelson et al, 2013 | RCT | AE=15, 69.10±5.00  AE and RE=16, 69.10±5.00  CG=17, 69.10±5.00 | 55 min×3  NR min×3 | 9months  9months | 0 | Yes | Portugal | BMI, SBP, DBP |
| LeMura et al, 2000 | RCT | RE=11, 20.00±1.00  AE,10, 21.00±2.00  Combination exercise=12, 19.00±2.00  CG=12, 20.00±1.00 | NR min×3  50 min×3  NR min×2 | 16weeks  16weeks  16weeks | 100.0 | Yes | USA | BMI, TC, HDL,  LDL |
| Mayra et al, 1995 | RCT | Brisk walking=16, 30.10±5.30  CG=11, 31.50±6.10 | NR min×4 | 40weeks | 100.0 | Yes | USA | TG, TC, HDL,  LDL |
| Ataeinosrat et al,2022 | RCT | Low-intensity interval  resistance training=15, 27.50±9.40  moderate-intensity interval  resistance training=15, 27.50±9.40  high-intensity interval  resistance training=15, 27.50±9.40  CG=15, 27.50±9.40 | 70 min×3  70 min×3  70 min×3 | 12weeks  12weeks  12weeks | 0 | NR | Iran | BMI,GLU |
| Okamoto et al,2022 | RCT | Isometric handgrip training=11, 76.00±2.00  CG=11, 74.00±2.00 | NRmin×5 | 8weeks | 59.1 | NO | Japan | SPB,DPB |
| Agust´ın et al,2022  **Table 1** *(continued)* | RCT | Pilates=55, 70.71±7.80  CG=52, 67.60±9.77 | 60min×2 | 12weeks | 100 | YES | Spain | BMI |

**Table 1** *(continued)*

**Table 1** *(continued)*

**Table 1** *(continued)*

**Table 1** *(continued)*

| Sources | Study  design | Exposure type,  sample size, age(mean±SD/range) | Proportion of  female(%) | Whether  supervision | Follow-up time | Region | Outcomes |
| --- | --- | --- | --- | --- | --- | --- | --- |
| Charles et al, 2014 | LS | SB=15,832 SB REF=16,067  PA=15,832 PA REF=15,801  40.00-79.00 | 60.9 | NR | 6.4years | USA | CVM |
| Kelly et al, 2016 | LS | SB=872 SB REF=842  PA=830 PA REF=2,529  40.00~ | NR | NR | 6.7years | USA | CVM |
| Ing-Mari et al, 2019 | LS | SB=406 SB REF=407  PA=411 PA REF=404  18.00-75.00 | 58.7 | NR | 15years | Sweden | CVD |
| Christina et al, 2014 | LS | SB=9,327 REF=26,207  18.00-99.00 | 59.2 | NR | 5.4years | Denmark | CVD |
| Charles et al, 2012 | LS | SB=37,107 REF=31,548  50.00-71.00 | 46.0 | NR | 8.5years | USA | CVM |
| Rebecca et al, 2014 | LS | SB=20,088 REF=48,567  50.00-79.00 | 100.0 | NR | 12years | USA | CVM,CVD |
| Emmanuel et al, 2011 | LS | SB=1,300 REF=771  40.00-75.00 | 56.1 | NR | 4.3years | UK | CVD |
| Gerrie-Cor et al, 2013 | LS | SB=2,062 REF=1,860  51.00-54.00 | 100.0 | NR | 9.9years | Australia | CVD |
| John et al, 2019 | LS | SB=1,410 REF=1,410  63.00-97.00 | 100.0 | NR | 4.9years | USA | CVD |
| Sources  **Table 1** *(continued)* | Study  design | Exposure type,  sample size, age(mean±SD/range) | Proportion of  female(%) | Whether  supervision | Follow-up time | Region | Outcomes |
| Andrea et al, 2013 | LS | SB=17,374 REF=24,691  50.00-79.00 | 100.0 | NR | 12.2years | USA | CVD |
| Jeanette et al, 2019 | LS | SB=1,112 PA=1,174  39.00-64.00 | 61.8 | NR | 8.4years | USA | CVD |
| Tatiana et al, 2011 | LS | SB=1,833 PA=2,011  20.00-89.00 | 0 | NR | 21years | USA | CVM |
| Peter et al, 2008 | LS | SB=822 REF=3,022  18.00-90.00 | 54.4 | NR | 12years | Canada | CVD |
| Charles et al,2015 | LS | SB=31,918 REF=13,634  59.00-82.00 | 49.3 | NR | 6.8years | USA | CVM |
| Ing-Mari et al, 2017 | LS | SB=3,937 REF=4,125  18.00-75.00 | NR | NR | 15years | Sweden | CVD |
| Deborah et al, 2014 | LS | SB=12,453 REF=34,899  45.00-69.00 | 0 | NR | 7.8years | USA | CVD |
| Michael et al, 2020 | LS | SB=29,526 PA=27,804  50.00-79.00 | 100.0 | NR | 9years | USA | CVD |
| Barbara et al,2018 | LS | SB=294 REF=296  40.00-59.00 | 0 | NR | 4.9years | UK | CVD |
| Xuanwen et al, 2021 | LS | PA=8,823 REF=4,832  54.00-72.00 | 55.7 | NR | 5years | China | CVD |
| Rune et al, 2019  **Table 1** *(continued)* | LS | PA=2,582 REF=4,864  40.00-62.00 | 42.4 | NR | 23.3years | Norway | CVM |
| Sources  **Table 1** *(continued)* | Study  design | Exposure type,  sample size, age(mean±SD/range) | Proportion of  female(%) | Whether  supervision | Follow-up time | Region | Outcomes |
| Emmanuel et al, 2009 | LS | PA=4,847 REF=5,064  35.00-74.00 | 59.9 | NR | 8.4years | Scotland | CVD |
| Iffat et al, 2014 | LS | PA=6,766 REF=7,171  47.00-84.00 | 100.0 | NR | 13years | Sweden | CVD |
| Eric et al, 2015 | LS | PA=7,749 REF=13,268  45.00~ | 81.1 | NR | 16.4years | USA | CVM |
| Peter et al, 2017 | LS | PA=337 REF=1,958  22.00-93.00 | 51.8 | NR | 33years | USA | CVD |
| Sangeeta et al, 2017 | LS | PA=2,262 REF=1,877  39.00-79.00 | 52.2 | NR | 18years | USA | CVD |
| Zhao et al, 2020 | LS | PA=587 REF=20,832  62.00-77.00 | 52.7 | NR | 6.8years | China | CVD |
| Zhao et al, 2019 | LS | PA=2,500 REF=36,702  40.00-85.00 | 50.4 | NR | 9.0years | USA | CVM |
| Kristin et al, 2011 | LS | PA=1,147 REF=949  41.00-70.00 | 100.0 | NR | 12years | Norway | CVM |
| Andreas et al, 2021 | LS | PA=7,017 REF=6,340  20.00-100.00 | 43.2 | NR | 10years | Denmark | CVD |
| Kazumune et al, 2010 | LS | PA=10,638 REF=25,925  65.00-84.00 | NR | NR | 6years | Japan | CVM |
| Manami et al, 2008 | LS | PA=18,209 REF=27,368  45.00-74.00 | NR | NR | 5years | Japan | CVD |
| Gary et al, 2017  **Table 1** *(continued)* | LS | PA=7,079 REF=39,947  46.00-70.00 | 54.3 | NR | 8.8years | UK | CVM |
| Sources | Study  design | Exposure type,  sample size, age(mean±SD/range) | Proportion of  female(%) | Whether  supervision | Follow-up time | Region | Outcomes |
| Herve et al, 2008 | LS | PA=NR REF=NR  45.00-79.00 | NR | NR | 7years | UK | CVM |
| Christine et al, 2011 | LS | PA=1,600 REF=407  25.00-74.00 | 48.8 | NR | 17.8years | Germany | CVD |
| Santos et al, 2022 | LS | PA=150,906 REF=190,080  18.00-84.00 | 55.4 | NR | 10.4years | USA | CVM |
| Lee et al,2022 | LS | PA=NR REF=NR  30.00-55.00 | NR | NR | 30years | USA | CVM |

AE, Aerobics exercise; AIE, Aerobics interval exercise; BMI, Body mass index; CVD, Cardiovascular disease; CVM, Cardiovascular mortality; DBP, Diastolic blood Pressure; EE, Endurance exercise; EG, Experimental group; EPG, Exposure group; GLU, Glucose; HDL, High density lipoprotein; HIE, High intensity exercise HIIE, High intensity interval exercise; LS, Longitudinal studies; LDL, Low density lipoprotein; MIE, Moderate intensity exercise; NR, Not reported; PA, Physical activity; RCT, Randomized controlled trial; RE, Resistance exercise; REF, Reference; SB, Sedentary behavior; SBP, Systolic blood pressure; SD, standard deviation; TG, Triglyceride; TC, Total cholesterol; UK, United Kingdom; USA, United States of America;

**Table 1** *(continued)*
